# Synthesis of *P*‑Modified Chimeric Oligonucleotides via Chemoselective Condensation of Nucleoside 3′‑*H*‑Phosphonothioate

**DOI:** 10.1021/acs.joc.5c01498

**Published:** 2025-08-25

**Authors:** Harumi Okutsu, Yuhei Takahashi, Tomomi Shiraishi, Utako Kashio, Kazuki Sato, Takeshi Wada

**Affiliations:** Department of Medicinal and Life Sciences, Faculty of Pharmaceutical Sciences, 26413 Tokyo University of Science, Katsushika-ku, Tokyo 125-8585, Japan

## Abstract

Nucleoside 3′-*H*-phosphonothioate monoesters possess two nucleophilic atoms, i.e., oxygen and sulfur atoms; therefore, their chemoselectivity must be controlled during condensation with a 5′-hydroxy group for the synthesis of oligonucleotides. However, the selective activation of a sulfur atom to form *H*-phosphonate diesters has been scarcely investigated. In this study, we examined the chemoselective condensation of *H*-phosphonothioate monoesters and developed a reaction to form two types of internucleotidic linkages, i.e., *H*-phosphonate and *H*-phosphonothioate diesters, from a single monomer. Using this approach, we synthesized a chimeric oligonucleotide bearing both *H*-phosphonate and *H*-phosphonothioate diester linkages on a solid support. By performing a global conversion reaction, phosphorodithioate/phosphorothioate chimeric dodecamers and phosphorothioamidate/phosphoramidate chimeric pentamers were successfully synthesized using only *H*-phosphonothioate monomers. The method developed herein offers a versatile tool for the synthesis of a wide variety of *P*-modified chimeric oligonucleotides, thus overcoming the difficulties in synthesizing these oligonucleotides by using existing techniques.

## Graphical abstract

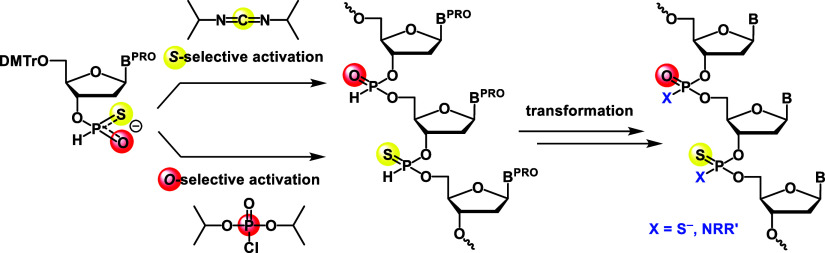

## Introduction

The synthesis of *P*-modified nucleotides and oligonucleotides has emerged as a promising strategy toward the development of pharmaceuticals with enhanced biological properties, such as antisense oligonucleotides (ASOs),^1^ siRNAs,^1^ and nucleotide analogs.^2^ Among these, phosphorothioate (PS)-modified oligodeoxynucleotides (ODNs) and oligoribonucleotides (ORNs), in which one of the nonbridging oxygen atoms in the phosphate backbone is replaced with a sulfur atom, are representative examples and have been approved as ASOs and siRNAs, respectively. PS-ODNs exhibit high nuclease resistance and favorable pharmacological profiles, conferring efficacy to oligonucleotide therapeutics.^1^ However, some PS-ODNs exhibit cytotoxicity.^3^ To develop potent ASOs with high safety and efficacy, many researchers have been enthusiastically investigating, and stereopure PS-ODNs offer favorable profiles.^4^ Thus, the synthesis of the stereopure PS-ODNs is an effective strategy, and some synthetic methods of stereopure PS-ODNs have been developed.^5^ On the other hand, the combination of PS modification with another type of *P*-modification having low cytotoxicity is also regarded as an effective strategy. To this end, various novel *P*-modified ODNs, such as mesylphosphoramidate,^6^ methoxypropylphosphonate,^7^ and phosphorylguanidine,^8^ were recently reported. Thus, the development of versatile synthetic methods for diverse *P*-modified ODNs is a critical issue for discovering promising *P*-modifications.

Although the phosphoramidite method is the most widely used for the synthesis of ODNs,^9^ the *H*-phosphonate method is an effective option for the synthesis of a wide variety of *P*-modified ODNs.^10,11^ *H*-Phosphonate diesters are precursors in the synthesis of various *P*-modified ODNs, such as a phosphodiesters,^12,13^ phosphorothioates,^14^ phosphoramidates,^15^ and alkylphosphonates.^16^ *H*-Phosphonate diesters are generally synthesized via the condensation of an *H*-phosphonate monoester with a 5′-hydroxy group using a condensing reagent. *H*-Phosphonothioate diesters, in which one of the nonbridging oxygen atoms of the *H*-phosphonate diester is replaced with a sulfur atom, are also versatile intermediates in the synthesis of various *P*-modified ODNs bearing sulfur atoms. Phosphorothioates,^17^ phosphorodithioates (PS_2_),^17^ phosphorothioamidates,^18^ arylphosphonothioates,^19,20^ and other derivatives can be synthesized via the transformation of an *H*-phosphonothioate diester. Notably, *H*-phosphonothioate diesters have enabled the synthesis of doubly *P*-modified ODNs that cannot be accessed via the phosphoramidite or *H*-phosphonate approach. Typically, an *H*-phosphonothioate diester is synthesized by condensing an *H*-phosphonothioate monoester with an appropriate alcohol. However, because *H*-phosphonothioate monoesters have oxygen and sulfur atoms as nucleophiles, it is necessary to control the chemoselectivity in the condensation reaction with 5′-hydroxy groups; as shown in Figure 1, *O*-selective activation would furnish an *H*-phosphonothioate diester, whereas *S*-selective activation would give an *H*-phosphonate diester.

**Figure 1:**
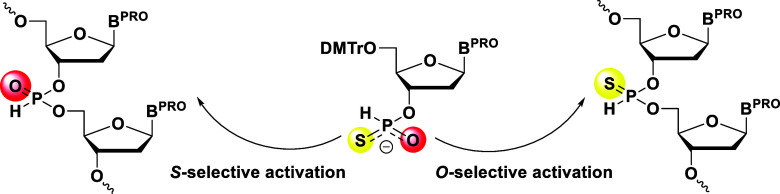
Chemoselective condensation of the *H*-phosphonothioate monoester.

Zain and Stawiński investigated the condensation reaction using a nucleoside 3′-*H*-phosphonothioate monoester with a variety of condensing reagents.^21^ As a result, diphenyl phosphorochloridate (DPCP) and diethyl phosphorochloridate (DECP) were found to be effective for the *O*-selective activation and formation of an *H*-phosphonothioate diester linkage in a chemoselective manner. In addition, our group synthesized *H*-phosphonothioate diester linkages from an *H*-phosphonothioate monoester using 3-nitro-1,2,4-triazol-1-yl-tris­(pyrrolidin-1-yl)­phosphonium hexafluorophosphate (PyNTP) as a condensing reagent.^22^

Conversely, the reaction conditions for the generation of *H*-phosphonate diester linkages via the *S*-selective activation of an *H*-phosphonothioate monoester have not yet been fully investigated. Zain and Stawiński reported the only example of the formation of an *H*-phosphonate diester from an *H*-phosphonothioate monoester.^21^ Contrary to their expectations, internucleotidic *H*-phosphonate diesters were generated using *N*,*N*′-diisopropylcarbodiimide (DIC) and pyridine hydrochloride (Py·HCl) in pyridine as a solvent in a ^31^P nuclear magnetic resonance (NMR) yield of 50% along with a small amount of *H*-phosphonothioate diesters (<5%), indicating a high chemoselectivity. However, the formation of the *H*-phosphonate monoester was significant as a byproduct (about same amount of the *H*-phosphonate diester); thus, the reaction conditions require optimization to minimize the formation of the *H*-phosphonate monoester to apply for the ODN synthesis.

In this study, we focused on developing a novel method for the synthesis of *H*-phosphonate diesters from *H*-phosphonothioate monoesters, aiming to enhance the utility of *H*-phosphonothioate monoesters as monomers of *P*-modified oligonucleotides. Apart from enabling the synthesis of two types of internucleotidic linkages from an *H*-phosphonothioate monomer, we envisioned that this strategy could be utilized to synthesize novel *P*-modified chimeric oligonucleotides by constructing *H*-phosphonate and *H*-phosphonothioate diester linkages from an *H*-phosphonothioate monoester and transforming internucleotidic linkages at the final stage of the synthesis (Figure 2). We demonstrated that this strategy was applicable to the synthesis of PS_2_/PS chimeric oligonucleotides via the sulfurization of both *H*-phosphonate and *H*-phosphonothioate diesters in the final step of the synthesis. In addition, by substitution of the sulfurization reaction with oxidative amination, a phosphorothioamidate (PSN)/phosphoramidate (PN) chimeric oligonucleotide was successfully synthesized.

**Figure 2:**
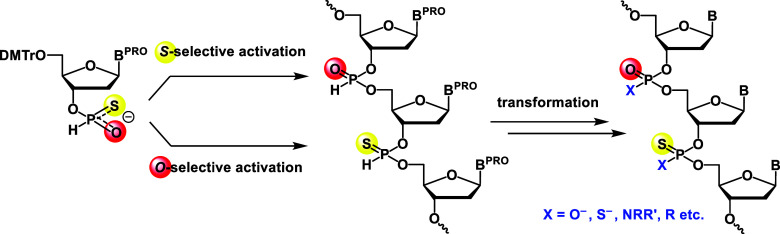
Synthesis of *P*-modified chimeric oligonucleotides from an *H*-phosphonothioate monoester.

## Result and Discussion

### Liquid-Phase Synthesis of an *H*-Phosphonate Diester from an *H*-Phosphonothioate Monoester

First, we adopted the reaction conditions reported by Zain and Stawiński^21^ with a minor modification (Scheme 1). Deoxycytidine 3′-*H*-phosphonothioate monoester **1c**, 1.2 equiv of compound **2** having a free 5′-hydroxy group, and 2 equiv of Py·HCl were dissolved in pyridine-*d*_5_ and thoroughly dried over molecular sieves 4A (MS 4A), followed by the addition of 3 equiv of DIC. After 15 min, the solution was analyzed by ^31^P NMR spectroscopy. The NMR yield was calculated as the integral ratio of the signals corresponding to the desired *H*-phosphonate diester **3** (d­(C_PO‑H_C), where PO-H denotes an *H*-phosphonate diester; δ 8–10) and all signals, and the chemoselectivity was estimated on the basis of the integral ratio of the desired *H*-phosphonate diester **3** to the undesired *H*-phosphonothioate diester **4** (d­(C_PS‑H_C), where PS-H denotes an *H*-phosphonothioate diester; δ 71–73).

**Scheme 1:**
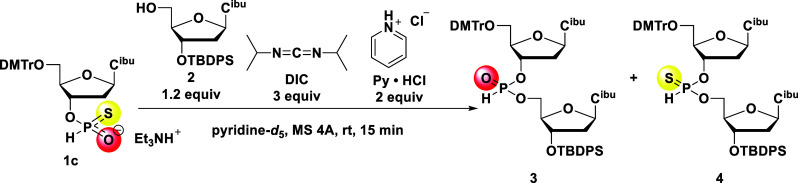
Zain’s Synthetic Conditions with a Minor Modification

As a result, we found that an internucleotidic *H*-phosphonate diester linkage **3** (δ 8.8, 9.8, ^1^ *J*_P–H_ = 714 Hz, ca. 76%) was formed in a chemoselective manner (**3**:**4** = 94:6) along with 5% of the *H*-phosphonothioate diester **4** (δ 73.0, 71.9, ^1^ *J*_P–H_ = 676.8 Hz) and ca. 18% of the 3′-*H*-phosphonate monoester (δ 3.8, ^1^ *J*_P–H_ = 618.4 Hz). This result was in good agreement with that reported by Zain and Stawiński.^21^ We attributed the formation of the *H*-phosphonate monoester to hydrolysis of the reaction intermediate. However, this hypothesis was ruled out, because the NMR yield of the *H*-phosphonate monoester remained at approximately 15% after carefully removing water from the reaction mixture. Since we found that using MeCN as a solvent suppressed the formation of the *H*-phosphonate monoester (see Supporting Information page S17), we proceeded to optimize the reaction conditions employing MeCN as the solvent (Table 1).

**Table 1:** Screening of Acidic Activators for the Formation of *H*-Phosphonate Diester **3** from the *H*-Phosphonothioate Monoester

entry	acidic activator	NMR yield of **3** (%)^a^	chemoselectivity PO-H/PS-H (3:4)^b^
1	Py·HCl	73	84:16
2	DCI	29	88:12
3	PhIMT	76	93:7
4	CMPT	71	92:8
5^c^	PhIMT	35	>99:1
6^c^	CMPT	41	>99:1

a Determined by the ^31^P NMR integral area ratio of d­(C_PO‑H_C) (**3**).

b Determined by the ^31^P NMR integral area ratios of d­(C_PO‑H_C) (**3**) and d­(C_PS‑H_C) (**4**).

c A preactivation procedure was conducted.

*H*-Phosphonothioate monoester **1c**, 1.2 equiv of compound **2**, and 2 equiv of an acidic activator were dissolved in CD_3_CN and dried over MS 3A. Subsequently, 2 equiv of DIC was added and the reaction mixture was analyzed by ^31^P NMR after 15 min. When Py·HCl was used as an acidic activator, the *H*-phosphonate monoester was not observed and the desired *H*-phosphonate diester was obtained in a 73% ^31^P NMR yield. However, the chemoselectivity deteriorated, resulting in a ratio of the *H*-phosphonate and *H*-phosphonothioate diester of 84:16 (Table 1, entry 1).

Therefore, we investigated several types of condensing reagents for the formation of the *H*-phosphonate diester (see details in the Supporting Information, Tables S1 and S2), finding that only using a carbodiimide-type condensing reagent with an acidic activator afforded the *H*-phosphonate diester in a chemoselective manner. We also found that the reaction did not proceed in the absence of an acidic activator, indicating that protonation of the carbodiimide is essential for this reaction.

Next, we screened various acidic activators for the DIC (Scheme 2).

**Scheme 2:**
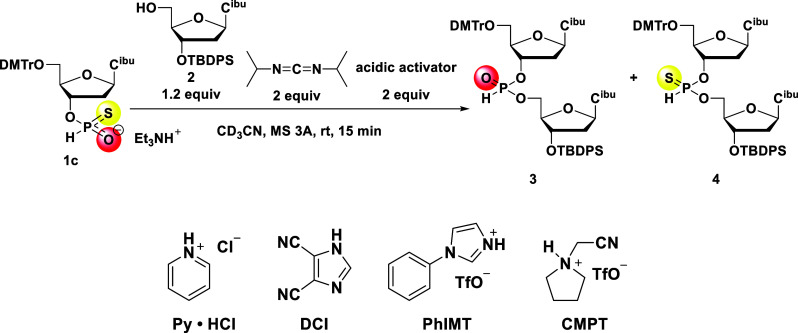
Screening of Acidic Activators for the Formation of *H*-Phosphonate Diester **3** from the *H*-Phosphonothioate Monoester

4,5-Dicyanoimidazole (DCI) gave a low NMR yield and similar chemoselectivity to that obtained with Py·HCl (Table 1, entry 2), whereas *N*-phenylimidazolium triflate (PhIMT)^23^ and (*N*-cynanomethyl)­pyrrolidinium triflate (CMPT)^24^ afforded the desired product with good chemoselectivity (**3**:**4** = 93:7 and 92:8, entries 3 and 4, respectively) and a moderate NMR yield (76% and 71%, entries 3 and 4, respectively). DCI and PhIMT are used as acidic activators in the phosphoramidite method. Meanwhile, CMPT is a non-nucleophilic acidic activator that is used for the stereocontrolled synthesis of *P*-modified oligonucleotides using the oxazaphospholidine method. Under the conditions described in entries 1–4, phosphite triester **10** was obtained as the main byproduct in ca. 10%–20% yield according to the ^31^P NMR. This byproduct was probably formed by the reaction of the activated monomer with 2 equiv of the 5′-hydroxy group. To improve chemoselectivity, we investigated various reaction conditions, finding that performing a preactivation procedure improved the chemoselectivity. Specifically, dissolving the *H*-phosphonothioate monomer and the acidic activator in dry CD_3_CN, followed by adding first DIC to initiate the reaction with the monomer and then compound **2** bearing a 5′-hydroxy group, resulted in the formation of the desired *H*-phosphonate diester **3** in a low NMR yield (35% and 41%, entries 5 and 6, respectively), along with many side reactions, including the formation of phosphite triester **10** (ca. 10%). Nevertheless, the absence of signals at δ 71–73 in the ^31^P NMR of the reaction mixture indicated that this procedure completely suppressed the formation of the *H*-phosphonothioate diester. When comparing the two conditions, the use of PhIMT (entry 5) afforded a slight amount of an unidentified byproduct, whereas CMPT (entry 6) improved the NMR yield. The ^31^P NMR spectra of these experiments are shown in the Supporting Information (Figures S23 and S24).

To elucidate the reaction mechanism under the preactivation conditions (entry 6), we monitored the reaction using ^31^P NMR spectroscopy (Scheme 3).

**Scheme 3:**
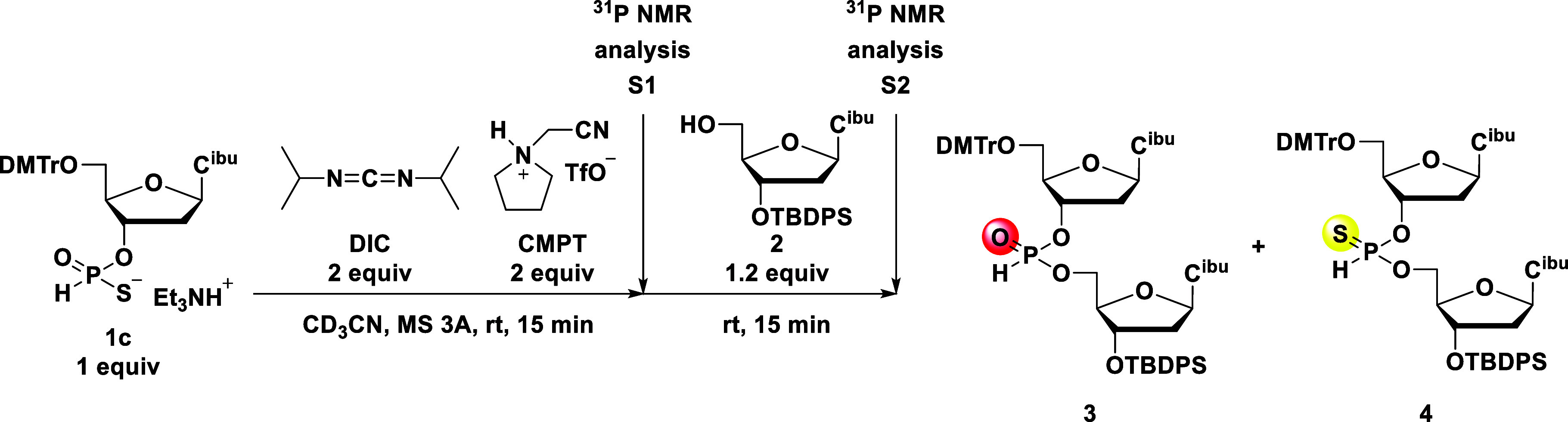
^31^P NMR Analysis of Using the Preactivation Procedure

First, *H*-phosphonothioate monomer **1c** and CMPT were dissolved in dry CD_3_CN and dried over MS 3A. Then, 2 equiv of DIC was added to preactivate the monomer. After 15 min, the reaction mixture was analyzed by ^31^P NMR to observe the reaction intermediates formed during the condensation (Figure S25, ^31^P NMR spectrum S1). Subsequently, the 5′-hydroxy group was added to monitor the formation of the products (Figure S25 and ^31^P NMR spectrum S2).

As a result, in the ^31^P NMR spectrum S1, broad signals were observed at δ 106–118 (ca. 34%) and at δ −3 to −1 (ca. 30%), which can be attributed to trimetaphosphite **7**,^25^ according to Garegg et al., and pyrophosphonate **8**,^25^ respectively. In addition, some of the signals at δ 106–118 and −3 to −1 might correspond to compound **9**. After the 5′-hydroxy group was added, these signals decreased and the signals of the desired *H*-phosphonate diester **3** and phosphite triester **10** appeared (42% and 10% yield in ^31^P NMR spectrum S2), respectively, whereas the signal of the undesired *H*-phosphonothioate diester **4** was not observed.

According to these results, the reaction intermediates responsible for the chemoselective formation of the *H*-phosphonate diester might be trimetaphosphite **7**, pyrophosphonate **8**, and compound **9** (Scheme 4). Since these compounds have no sulfur atoms, the desired *H*-phosphonate diester **3** was considered to be obtained with complete chemoselectivity. Conversely, under the conditions without the preactivation protocol, since the sulfur atom of an *H*-phosphonothioate monoester might be complete selectively activated by DIC (Scheme S4), the participation of the pyrophosphonothioate **6** as one of the reaction intermediates cannot be excluded. Owing to the presence of two types of phosphorus atoms in pyrophosphonothioate **6**, one bearing a P=O bond and the other a P=S bond, the reaction of this intermediate with a 5′-hydroxy group would afford *H*-phosphonate and *H*-phosphonothioate diesters (Scheme 4), deteriorating the chemoselectivity. Details of plausible reaction mechanisms and additional investigations are shown in the Supporting Information (Schemes S5 and S6).

**Scheme 4:**
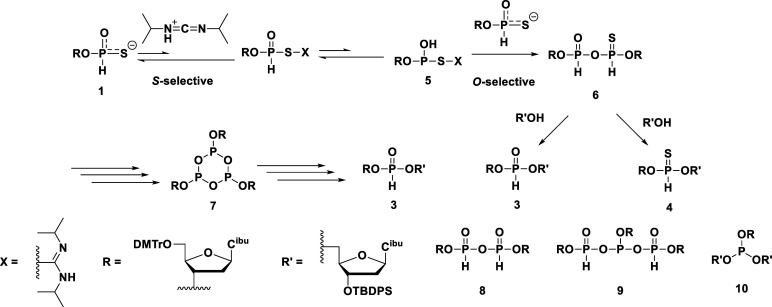
Plausible Mechanism for the Formation of the *H*-Phosphonate Diester

Although the low NMR yield obtained under the preactivation conditions (Table 1, entry 6) was at first disappointing, we envisioned that this method would be suitable for selectively generating the desired internucleotidic *H*-phosphonate diester linkage via solid-phase synthesis because excess monomer is typically used in the solid-phase synthesis for the reaction with the 5′-hydroxy group on a solid support to compensate for the low reaction efficacy of solution–solid reactions compared with solution–solution reactions. In this case, the monomer is activated in the supernatant and then the reaction intermediate reacts with the 5′-hydroxy group on the solid support to form internucleotidic linkages, in a similar manner to the preactivation protocol. In addition, in the solid-phase synthesis, owing to the immobilization of the nucleoside bearing a 5′-hydroxy group on the solid support, the increased distance between nucleosides compared to the liquid-phase synthesis would suppress the formation of phosphite triester **10**. Based on these hypotheses, we investigated the solid-phase synthesis of the internucleotidic linkage.

### Solid-Phase Synthesis of Dinucleoside Phosphorothioates via an *H*-Phosphonate Diester

To prove our hypothesis, we conducted the solid-phase synthesis of a phosphorothioate dimer, as shown in Scheme 5 and Table 2. To a mixture of deoxycytidine 3′-*H*-phosphonothioate derivative **1c** and a solid support bearing a free 5′-hydroxy group in a reaction vessel were successively added solutions of DIC and CMPT in MeCN to form an internucleotidic linkage. After 15 min, detritylation was performed with 3% DCA in CH_2_Cl_2_, followed by treatment with 0.1 M Et_3_N and 0.3 M S_8_ in CH_2_Cl_2_ to convert the *H*-phosphonate diester to its PS counterpart. Subsequently, ammonia treatment was conducted to deprotect the nucleobases and release the product from the solid support to obtain the target phosphorothioate dimer. The mixture was analyzed by reversed-phase HPLC (RP-HPLC), and the yield and chemoselectivity were determined by comparing the area ratios of desired dinucleoside phosphorothioate **11** and undesired phosphorodithioate **12**.

**Scheme 5:**
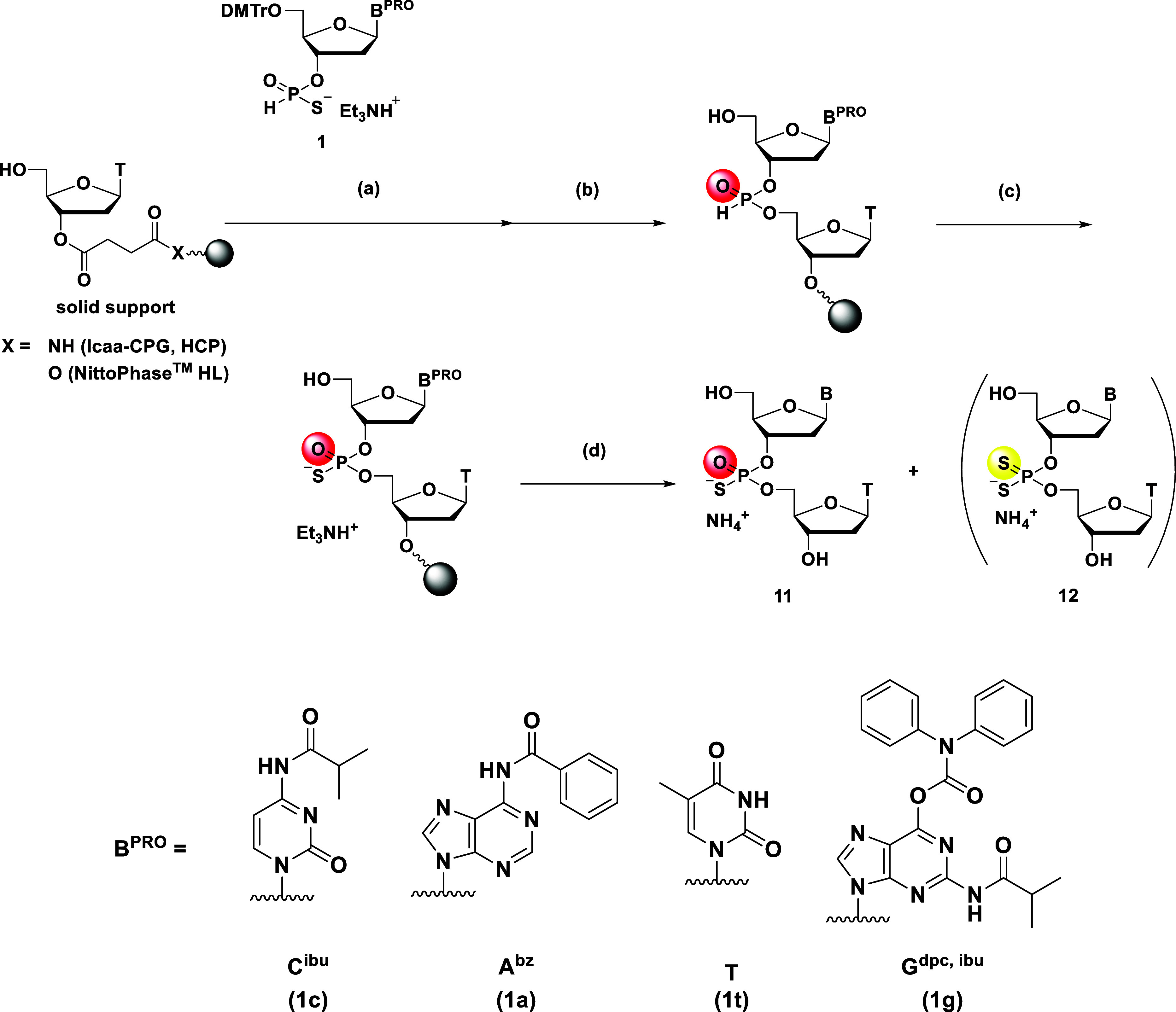
Solid-Phase Synthesis of the d­(N_PS_T) Dimer via an *H*-Phosphonate Diester

a 0.1 M *H*-phosphonothioate monomer **1**, 0.2 M DIC, and 0.2 M CMPT in MeCN, rt, 15 min.
b 3% DCA, CH_2_Cl_2_, rt, 4 × 10 s for entries 1 and 2 or 8 × 10 s for entries 3–8 (Table 2).
c 0.1 M Et_3_N, 0.3 M S_8_, CH_2_Cl_2_, rt, 90 min.
d Concentrated aqueous NH_3_–EtOH (3:1, v/v), 55 °C, 18 h.

**Table 2:** Solid-Phase Synthesis of the d­(N_PS_T) Dimers via an *H*-Phosphonate Diester

entry	product	solid support	HPLC yield of 11 (%)^a^	chemoselectivity PS/PS_2_ (11:12)^b^
1	dC_PS_T (**11ct**)	lcaa-CPG	81	93:7
2	dC_PS_T (**11ct**)	HCP	85	93:7
3	dC_PS_T (**11ct**)	NittoPhase HL	87	97:3
4^c^	dC_PS_T (**11ct**)	NittoPhase HL	96	>99:1
5^c^	dA_PS_T (**11at**)	NittoPhase HL	95	>99:1
6^c^	T_PS_T (**11tt**)	NittoPhase HL	97	>99:1
7^c^	dG_PS_T (**11gt**)	NittoPhase HL	89	>99:1
8^c^,^d^	dG_PS_T (**11gt**)	NittoPhase HL	90	>99:1

a Determined by the crude RP-HPLC area ratio of d­(N_PS_T) (**11**) and all products excluding bzNH_2_.

b Determined by the crude RP-HPLC area ratios d­(N_PS_T) (**11**):d­(N_PS2_T) (**12**).

c The preactivation procedure was conducted.

d The condensation reaction was performed for 20 min.

First, long chain alkylamino-controlled pore glass (lcaa-CPG) was utilized as a solid support (Table 2, entry 1), affording phosphorothioate **11**/phosphorodithioate **12** in a 93:7 ratio and an HPLC yield of the desired phosphorothioate **11** of 81%. Using highly cross-linked polystyrene (HCP)^26^ improved the HPLC yield to 85% (entry 2). Notably, the slightly swellable polystyrene-based support NittoPhase HL gave the highest chemoselectivity (**11**:**12** = 97:3) and HPLC yield (87%). As expected, the solid-phase synthesis afforded promising results compared with those of the liquid-phase synthesis.

In addition, the results obtained under the conditions in Table 2, entries 1–3, led us to conclude that NittoPhase HL is the most effective platform for the formation of an *H*-phosphonate diester. Furthermore, the condensation reaction on NittoPhase HL was conducted with monomer preactivation (entry 4). Briefly, solutions of DIC and CMPT in MeCN were sequentially added to the monomer in a round-bottom flask, and the reaction was conducted for 15 min. Then, the resulting reaction mixture was transferred to a vessel for solid-phase synthesis and allowed to react for an additional 15 min. Subsequently, the same procedures described previously were conducted, affording the desired phosphorothioate dimer with a chemoselectivity of >99:1 and an HPLC yield of 96%. Thus, the preactivation strategy proved to be also effective for the solid-phase synthesis. As shown in entries 5–8, we examined *H*-phosphonothioate monomers bearing various nucleobases. To prevent undesired side reactions on the *O* ^6^ position of guanine, a diphenylcarbamoyl (dpc) group^27^ was introduced to the deoxyguanosine 3′-*H*-phosphonothioate monomer in addition to the standard isobutyryl protection at the *N* ^2^ position. Although the synthesis using dA and T monomers was successful (entries 5 and 6), the HPLC yield of dG_PS_T was not satisfactory (89%; entry 7). Extending the condensation reaction time of the preactivated monomer and the 5′-hydroxy group on the solid support to 20 min slightly improved the HPLC yield to 90% (entry 8); thus, the conditions in entry 8 were selected as the optimal ones.

These findings indicated that the solid-phase synthesis provided a substantial enhancement in both yield and chemoselectivity, consistent with our initial hypothesis. In addition, the choice of the solid support considerably affected the outcome, with NittoPhase HL affording the best yield and chemoselectivity. Notably, the preactivation method in the solid-phase synthesis resulted in further gains in yield and chemoselectivity. The success in the solid-phase synthesis of dimers via an *H*-phosphonate diester prompted us to apply this method to the synthesis of chimeric oligonucleotides via *H*-phosphonate and *H*-phosphonothioate diester linkages.

### Solid-Phase Synthesis of Dinucleoside Phosphorodithioates via an *H*-Phosphonothioate Diester

Next, we examined the conditions for the *H*-phosphonothioate diester formation from *H*-phosphonothioate monoesters using NittoPhase HL. Zain and Stawiński successfully generated *H*-phosphonothioate diester linkages by selectively activating the oxygen atom using DPCP or DECP,^21^ and Kamaike et al. reported an efficient solid-phase synthesis of PS_2_-ODNs using *H*-phosphonothioate monomers and phosphorochloridate derivatives on a CPG support.^28^ Furthermore, we demonstrated the highly selective formation of *H*-phosphonothioate diester linkages using a phosphonium-type condensing reagent PyNTP on an HCP support.^22^ To investigate the optimal reaction conditions on NittoPhase HL, the synthesis of a dinucleoside phosphorodithioate was conducted as follows: Deoxyadenosine 3′-*H*-phosphonothioate derivative **1a** was reacted with the 5′-hydroxy group of thymidine loaded on NittoPhase HL in the presence of a condensing reagent and a base without preactivation. Then, the DMTr group on the 5′-end was removed, and the internucleotidic linkage was sulfurized by treatment with S_8_ and Et_3_N. After treatment with concentrated aqueous NH_3_–EtOH to cleave the linker and deprotect the nucleobases, the reaction mixture was analyzed by RP-HPLC (Scheme 6), and the HPLC yield and chemoselectivity were determined by comparing the area ratios of the desired dinucleoside phosphorodithioate **12** and the undesired phosphorothioate **11**.

**Scheme 6:**
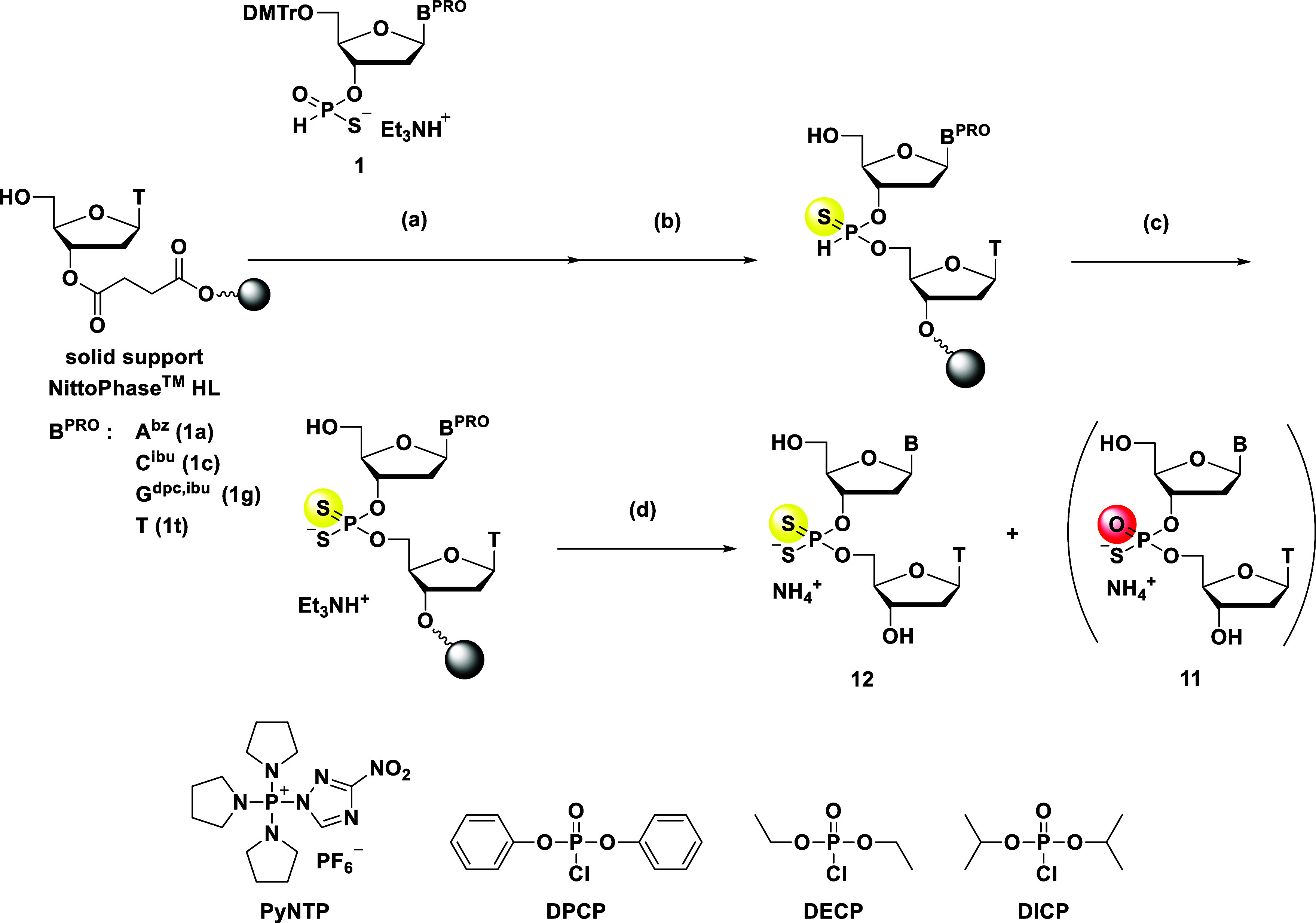
Solid-Phase Synthesis of the d­(N_PS2_T) Dimers via an *H*-Phosphonothioate Diester

a 0.1 M *H-*phosphonothioate monomer **1**, condensing reagent, base, MeCN, rt, time.
b 3% DCA, CH_2_Cl_2_, rt, 8 × 10 s.
c 0.1 M Et_3_N, 0.3 M S_8_, CH_2_Cl_2_, rt, 90 min.
d Concentrated aqueous NH_3_–EtOH (3:1, v/v), 55°C, 18 h.

The results are shown in Table 3.

**Table 3:** Solid-Phase Synthesis of the d­(N_PS2_T) Dimers via an *H*-Phosphonothioate Diester

entry	product	condensing reagent (M)	base (%, v/v)	time (min)	HPLC yield of 12 (%)^a^	chemoselectivity PS/PS_2_ (11:12)^b^
1	dA_PS2_T (**12at**)	PyNTP (0.25)	quinoline (20)	3	50	38:62
2	dA_PS2_T (**12at**)	DPCP (0.15)	pyridine (20)	3	93	6:94
3	dA_PS2_T (**12at**)	DECP (0.15)	pyridine (20)	3	97	2:98
4	dA_PS2_T (**12at**)	DICP (0.15)	pyridine (20)	5	96	1:99
5	dA_PS2_T (**12at**)	DICP (0.15)	pyridine (10)	5	97	1:>99
6	dC_PS2_T (**12ct**)	DICP (0.15)	pyridine (10)	5	98	1:>99
7	dG_PS2_T (**12gt**)	DICP (0.15)	pyridine (10)	5	93	1:>99
8	T_PS2_T (**12tt**)	DICP (0.15)	pyridine (10)	5	98	1:>99

a Determined by the crude RP-HPLC area ratio of dN_PS2_T (**12**) excluding bzNH_2_.

b Determined by the crude RP-HPLC area ratios dN_PS_T (**11**)/dN_PS2_T (**12**).

As an initial attempt, we used the conditions of our previous report,^22^ namely, PyNTP and quinoline as a condensing reagent and a base, respectively; however, these conditions afforded the desired phosphorodithioate dimer **12** in a low HPLC yield of 50% with poor chemoselectivity (**11**:**12** = 38:62; entry 1). A plausible reason for the low chemoselectivity is discussed in the Supporting Information (Scheme S7 and Figure S46). In contrast, employing DPCP and 20% v/v pyridine as described Kamaike et al.^28^ considerably improved both the HPLC yield (93%) and the chemoselectivity (**11**:**12** = 6:94). Further selectivity enhancement was observed with DECP (entry 3), leading to a high chemoselectivity (**11**:**12** = 2:98) and a concomitant increase in the HPLC yield to 97%. Notably, using diisopropyl phosphorochloridate (DICP) in conjunction with 20% v/v pyridine (entry 4) resulted in a dramatic improvement in chemoselectivity (**11**:**12** = 1:99) while maintaining a good HPLC yield (96%). Reducing the pyridine concentration to 10% v/v ultimately afforded the desired phosphorodithioate dimer **12** with a chemoselectivity of >99% and slightly improved HPLC yield of 97% (entry 5). These results suggested that DICP was the most effective condensing reagent, leading to both high efficiency and chemoselectivity, which can be attributed to the bulky and less electron-withdrawing nature of the isopropyl groups modulating the electrophilicity of the phosphorochloridate. Using the *H*-phosphonothioate monomers bearing different nucleobases for the dimer synthesis (entries 6–8) furnished the desired phosphorodithioate dimers in good yields and with excellent chemoselectivities. Thus, it can be concluded that these conditions are suitable for oligonucleotide synthesis.

### Solid-Phase Synthesis of PS_2_/PS Chimeric Oligonucleotides

With the optimized conditions for the formation of both *H*-phosphonate and *H*-phosphonothioate diesters from *H*-phosphonothioate monoesters in hand, we addressed the synthesis of chimeric oligonucleotides. First, we selected PS_2_/PS chimeric oligonucleotides as the synthetic target and conducted the synthesis of a d­(T_PS_C_PS2_A_PS_G_PS2_T) pentamer under the conditions shown in Scheme 7. Four cycles of condensation and detritylation were repeated to obtain an oligomer bearing both *H*-phosphonate and *H*-phosphonothioate diesters on a solid support. Subsequently, sulfurization of the internucleotidic linkages was performed, followed by nucleobase deprotection and release from the solid support via ammonia treatment. The resultant mixture was then analyzed by reversed-phase UPLC-MS (RP-UPLC-MS).

**Scheme 7:**
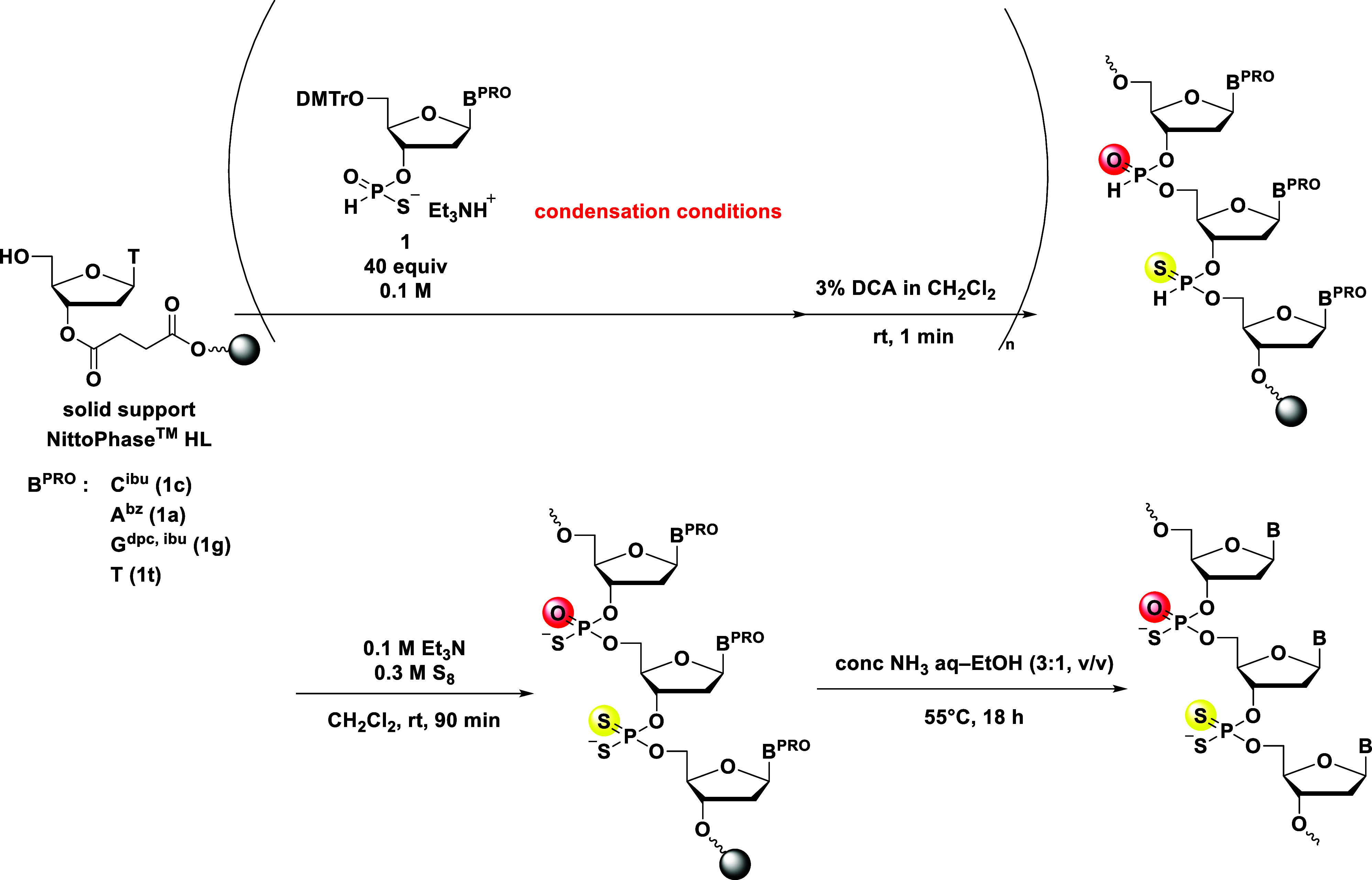
Solid-Phase Synthesis of PS_2_/PS Chimeric Oligonucleotides^a^

a Condensation conditions for the formation of the *H*-phosphonate diester: 0.2 M DIC and 0.2 M CMPT in dry MeCN, rt, 15 min (preactivation protocol); for the dG monomer, the condensation reaction was performed for 20 min. Condensation conditions for the formation of the *H*-phosphonothioate diester: 0.15 M DICP in 10% v/v pyridine and MeCN, rt, 5 min.

RP-UPLC-MS analysis confirmed the successful synthesis of the target PS_2_/PS chimeric pentamer d­(T_PS_C_PS2_A_PS_G_PS2_T) (**13**) in 76% UPLC yield (Figure 3). Moreover, the pentamer was isolated in 32% yield (the UPLC profile of purified **13** is shown in the Supporting Information, Figure S47).

**Figure 3:**
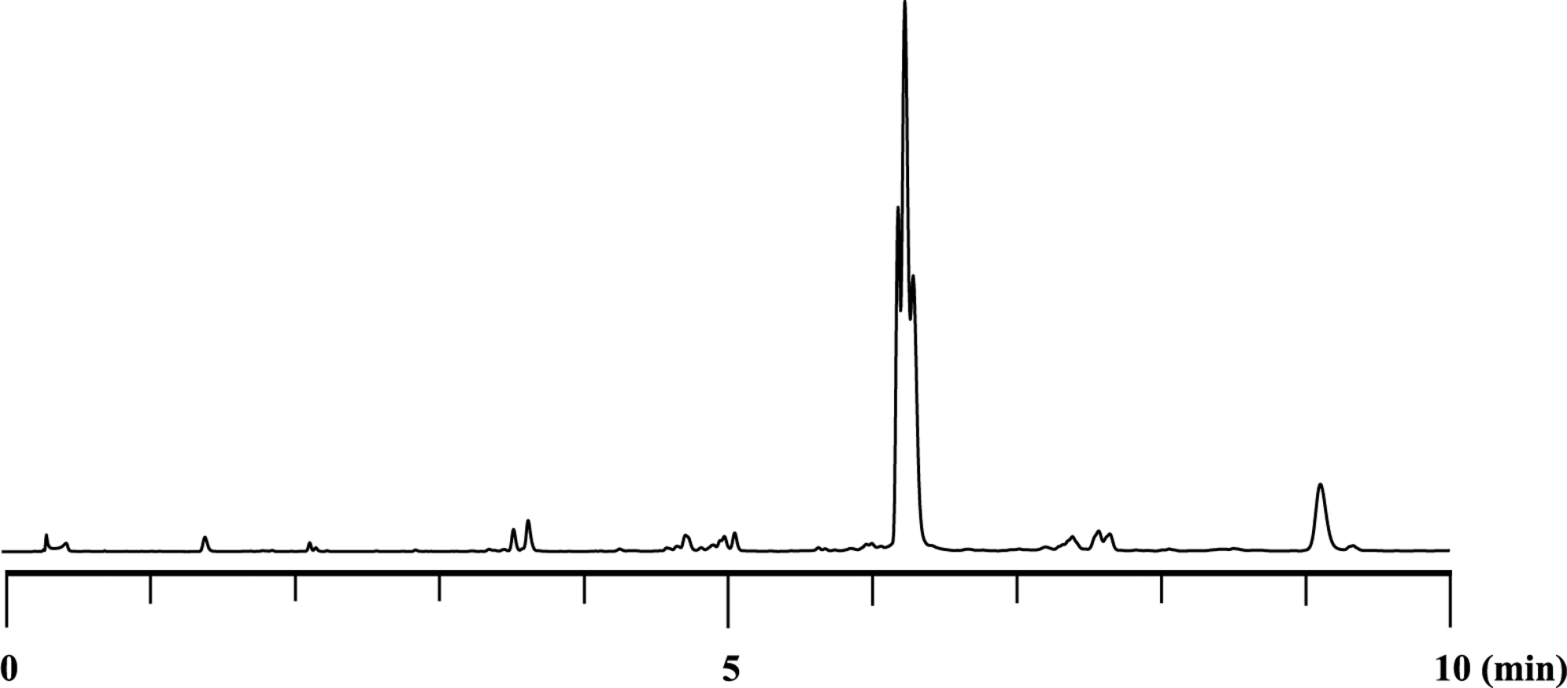
RP-UPLC profile of crude **13**. Diastereomers of compound **13** were eluted at 6.2–6.4 min.

This result demonstrated the compatibility of the sulfurization conditions for both the *H*-phosphonate and *H*-phosphonothioate diester linkages, which prompted us to extend this methodology to the synthesis of a dodecamer. After 11 iterative cycles of condensation and detritylation, followed by sulfurization of the internucleotidic linkages and subsequent ammonia treatment for nucleobase deprotection and release from the solid support, desired dodecamer d­(C_PS2_A_PS_G_PS2_T_PS_C_PS_A_PS2_G_PS_T_PS2_C_PS2_A_PS_G_PS2_T) (**14**) was obtained. The RP-UPLC-MS analysis of the crude mixture confirmed the successful synthesis of the PS_2_/PS chimeric dodecamer in 33% UPLC yield (Figure 4). Moreover, the dodecamer was isolated in 11% yield (the UPLC profile of purified **14** is shown in the Supporting Information, Figure S48). UPLC-MS analysis of the reaction mixture revealed that the major byproduct was an undecamer that lacked a dG_PS_ moiety. Considering that the HPLC yield of dG_PS_T was relatively low, efficiency for the formation of an *H*-phosphonate diester linkage using a deoxyguanosine monomer was also insufficient in the dodecamer synthesis. Additionally, a tridecamer which has an additional dA_PS_ moiety was observed, suggesting that partial detritylation proceeded during the condensation reactions using the dA monomer for the *H*-phosphonate diester formation. These issues should be addressed to improve the isolated yield of oligomers.

**Figure 4:**
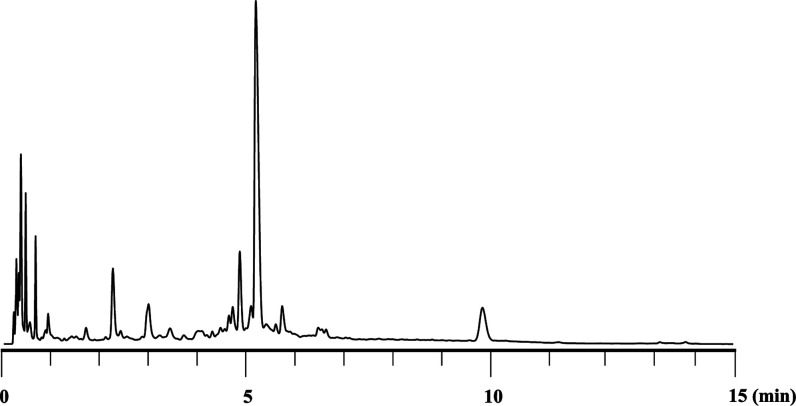
RP-UPLC profile of crude **14**. Diastereomers of compound **14** were eluted at 5.2–5.4 min.

In addition, since there is a possibility that each condensation reaction provided an undesired internucleotidic linkage, MS analysis of the full-length product was not enough to prove that the synthesized oligonucleotides had *P*-modifications at the intended positions. Thus, to confirm the structure of the pentamer and dodecamer, LC-MS/MS analysis was conducted. Collision-induced dissociation experiments were used to produce the fragment ions via cleavage of an internucleotidic linkage. The observed *m*/*z* values for the fragment ions were in good agreement with the theoretical values for the target sequences, unambiguously confirming the successful synthesis and isolation of the desired chimeric oligonucleotides. The complete MS/MS spectral data are in the Supporting Information (Figures S49–S57).

Recently, the PS_2_ modification in ASOs,^29^ siRNA,^30^ and nucleotide analogs^31^ has been intensively investigated. Because the PS_2_ modification eliminates the chirality at the phosphorus center, its combination with PS modification maintains the in vivo activity while reducing the number of stereoisomers. The synthesis of PS_2_/PS chimeric ODNs has relied on the use of two distinct types of monomers: general phosphoramidite monomers and phosphorothioamidite monomers. However, phosphorothioamidites are prone to oxidation, even when taking precautions to remove oxygen.^32^ In contrast, the present methodology represents the first example of the synthesis of PS_2_/PS chimeric ODNs employing a stable *H*-phosphonothioate monomer.

### Synthesis of PSN/PN Chimeric ODNs

Under the established conditions, we proceeded to synthesize PSN/PN chimeric ODNs via *H*-phosphonate and *H*-phosphonothioate diester linkages by using an oxidative amination reaction. Employing morpholine as the amine, we optimized the conditions for the oxidative amination by synthesizing dimers. Full experimental details can be found in the Supporting Information (Scheme S8 and Table S3).

After performing four cycles of condensation and detritylation on NittoPhase HL, the internucleotidic linkages were subjected to an oxidative amination reaction using a mixture of CCl_4_–MeCN–morpholine (4.5:4.5:1, v/v/v). Subsequent ammonia treatment for nucleobase deprotection and release from the solid support gave the desired PSN/PN chimeric pentamer, d­(T_PN_C_PSN_A_PN_G_PSN_T) (**15**) (Scheme 8). The RP-UPLC-MS analysis confirmed the successful synthesis of the PSN/PN chimeric pentamer (42% UPLC yield), which was isolated in a 16% yield (the UPLC profiles of crude and purified **15** are shown in the Supporting Information, Figures S64 and S65). Subsequently, LC-MS/MS analysis indicated the desired *P*-modifications were successfully introduced (the complete MS/MS spectral data are available in the Supporting Information, Figures S66 and S67).

**Scheme 8:**
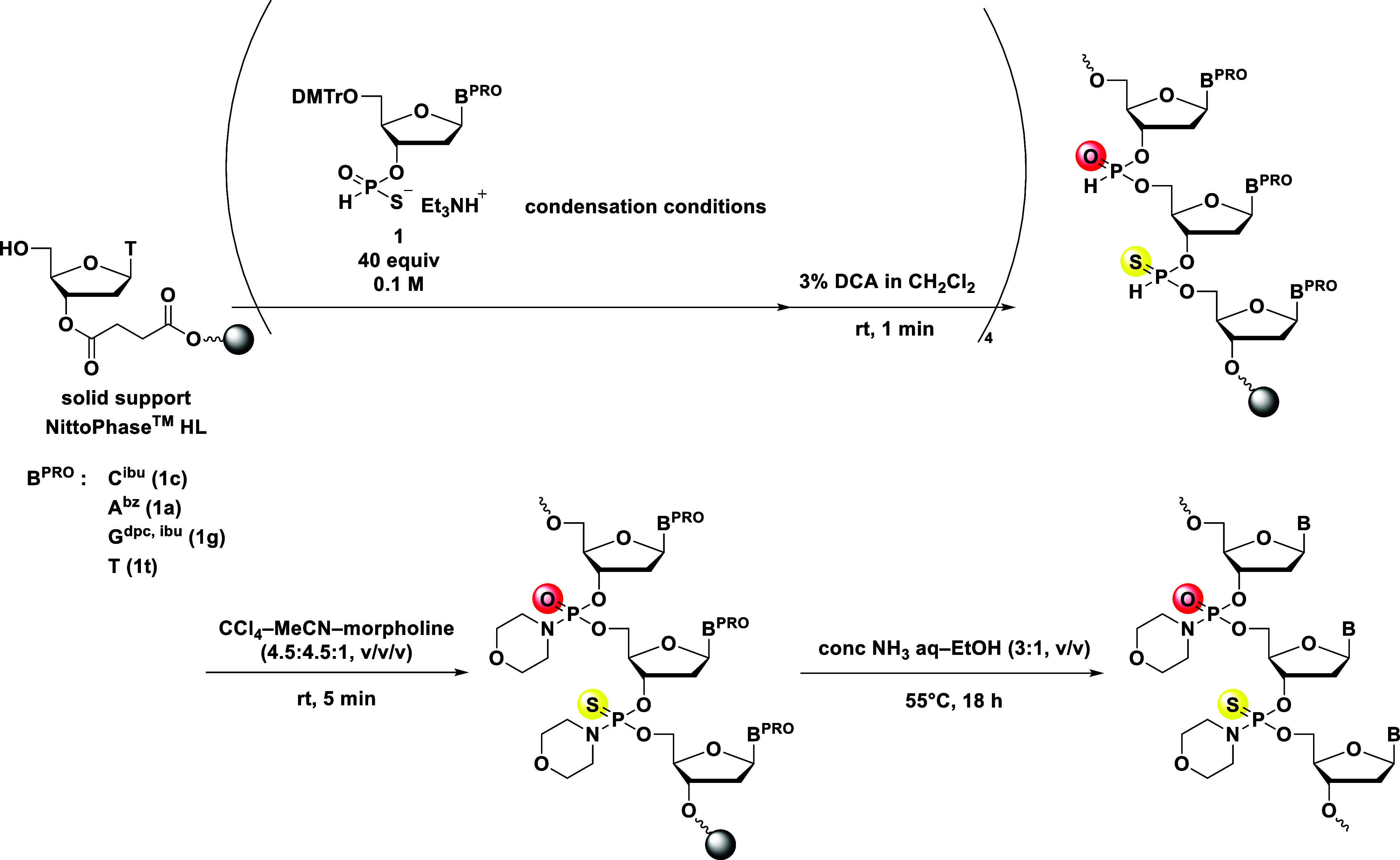
Solid-Phase Synthesis of the PSN/PN Chimeric Pentamer (d­(T_PN_C_PSN_A_PN_G_PSN_T)) (**15**)­^a^

a Condensation conditions for the formation of the *H*-phosphonate diester: 0.2 M DIC and 0.2 M CMPT in dry MeCN, rt, 15 min (pre-activation protocol); for the dG monomer, the condensation reaction was performed for 20 min. Condensation conditions for the formation of the *H*-phosphonothioate diester: 0.15 M DICP in 10% v/v pyridine and MeCN, rt, 5 min.

These results demonstrate that replacing the sulfurization in the internucleotidic linkage transformation with oxidative amination is a viable strategy for accessing PSN/PN chimeric ODNs. This methodology enabled the synthesis of a new type of chimeric *P*-modified ODNs, which was previously synthetically challenging.

## Conclusion

In this study, we investigated the conditions for the chemoselective condensation of nucleoside 3′-*H*-phosphonothioate monoesters, particularly focusing on the formation of *H*-phosphonate diester linkages during liquid-phase synthesis. Consequently, we found that using DIC, a carbodiimide condensing reagent, in conjunction with CMPT, an acidic activator whose conjugate base lacks nucleophilicity, enabled the highly selective formation of *H*-phosphonate diester linkages. Furthermore, the preactivation of the monomer with the condensing reagent followed by the reaction with a 5′-hydroxy group resulted in the successful formation of the desired *H-*phosphonate diester linkages with >99% chemoselectivity. Although the NMR yield of the liquid-phase synthesis was moderate, this method afforded dimers with high HPLC yields and excellent chemoselectivities in the solid-phase synthesis, achieving >99% chemoselectivity for the desired *H*-phosphonothioate diester linkages. On the basis of these findings, we investigated the synthesis of *P*-modified chimeric ODNs including double *P*-modifications. After the synthesis of an oligomer bearing both *H*-phosphonate and *H*-phosphonothioate diesters using nucleoside 3′-*H*-phosphonothioate monomers, sulfurization of the internucleotidic linkages afforded PS_2_/PS chimeric ODNs. Importantly, we further expanded the scope of this methodology by demonstrating that the oxidative amination reaction can be employed in place of sulfurization for the efficient synthesis of unprecedented PSN/PN chimeric ODN derivatives. This novel approach provides a versatile platform for the efficient synthesis of a wide range of *P*-modified chimeric and doubly *P*-modified chimeric oligonucleotides with potential applications in diverse fields such as chemical biology and therapeutics.

## Experimental Section

### General Information

All reactions reported herein were performed under an argon atmosphere. Organic solvents were dried by using appropriate methods.

Compounds **S1a**, **S1c**, and **S1t** were of commercial grade (ChemGenes Corporation).

^1^H NMR spectra were recorded in CDCl_3_ and CD_3_CN using tetramethylsilane (δ 0.00) as an internal standard at 400 MHz (JEOL JNM 400) or 500 MHz (Bruker Avance NEO 500). ^13^C NMR spectra were recorded in CD_3_CN using CD_3_CN (δ 1.32) as an internal standard at 126 MHz on a 500 MHz Bruker Avance NEO 500. ^31^P NMR spectra were recorded in CD_3_CN and Pyridine-*d*_5_ using 85% H_3_PO_4_ (δ 0.0) as an external standard at 161 MHz (on a 400 MHz JEOL JNM 400), 243 MHz (on a 600 MHz Bruker Avance 600), or 202 MHz (on a 500 MHz Bruker Avance NEO 500). gCOSY, gHSQC, and gHMBC spectra were recorded on a 500 MHz Bruker Avance NEO 500.

Structural Assignments Were Made with Additional Information from gCOSY, gHSQC, and gHMBC Experiments

Purification by silica gel column chromatography was performed by automated flash chromatography using a Yamazen UNIVERSAL Premium column (30 μm, 60 Å) on an automated flash chromatography system W-prep 2XY. Thin-layer chromatography (TLC) was performed on TLC plates of Silica gel 60 F_254_ (Merck, No. 5715).

The synthesized dimers were analyzed by RP-HPLC (JASCO chromNAV system equipped with a JASCO PU-2080i plus pump and a JASCO UV-2075 plus as a UV/vis detector) using a C_18_ column (Delta pak 5 μm C_18_ column, 100 Å, 3.9 × 150 mm, Waters). The synthesized pentamers and dodecamers were analyzed and purified by UPLC (ACQUITY Premier, Waters) using a C_18_ column (ACQUITY Premier BEH C_18_ 1.7 μm, 2.1 × 50 mm, 130 Å, Waters).

Isolated yields of oligomers were calculated by a UV absorbance of UV spectrum (260 nm) using a UV spectrometer (SHIMADZU, UV-1900i). Molar extinction coefficients (ε) at 260 nm were estimated as 48,100 M^–1^ cm^–1^ for **13** and **15** and 118,100 M^–1^ cm^–1^ for **14**.

The LC-MS/MS analysis was conducted with CONFIRM Sequence App 1.4.0.13 and SELECT SERIES Cyclic IMS (Waters). Precursor and fragment ion mass tolerance was ±10 ppm.

### General procedure for the investigation of the acidic activator for the formation of an *H*-phosphonate diester from an *H*-phosphonothioate monomer via the liquid-phase synthesis. Synthesis without the Preactivation Protocol (Table 1, Entries 1–4)

5′-*O*-(Dimethoxytrityl)-*N* ^4^-isobutyryldeoxycytidine-3′-*H*-phosphonothioate monoester **1c** (triethylammonium salt, 39.2 mg, 50 μmol), 3′-*O*-(*tert*-butyldiphenylsilyl)-*N* ^4^-isobutyryldeoxycytidine (**2**, 32.1 mg, 60 μmol), and an acidic activator (pyridine hydrochloride 11.6 mg, 100 μmol in entry 1; 4,5-dicyanoimidazole 11.8 mg 100 μmol in entry 2; *N*-phenylimidazolium triflate 29.4 mg, 100 μmol in entry 3; CMPT 26.0 mg, 100 μmol in entry 4) in CD_3_CN (1 mL) were dried over MS 3A for at least 60 min. Then, *N*,*N*′-diisopropylcarbodiimide (DIC) (15.5 μL, 100 μmol) was added. After 15 min, the solution was analyzed by ^31^P NMR spectroscopy.

### Synthesis with the Preactivation Protocol (Table 1, Entry 5)

5′-*O*-(Dimethoxytrityl)-*N* ^4^-isobutyryldeoxycytidine-3′-*H*-phosphonothioate monoester **1c** (triethylammonium salt, 39.2 mg, 50 μmol) and *N*-phenylimidazolium triflate (29.4 mg, 100 μmol) in dry CD_3_CN (600 μL) were dried over MS 3A for 60 min in a reaction vessel. Then, DIC (15.5 μL, 100 μmol) was added to the reaction vessel. After 15 min, the solution was analyzed by ^31^P NMR spectroscopy. Subsequently, a solution of 3′-*O*-(*tert-*butyldiphenylsilyl)-*N* ^4^-isobutyryldeoxycytidine (**2**, 32.1 mg, 60 μmol) in CD_3_CN (100 μL) was added to the NMR sample tube, and the reaction mixture was shaken using vortex stirring. After 15 min, the solution was analyzed by ^31^P NMR spectroscopy.

### Synthesis with the Preactivation Protocol (Table 1, Entry 6)

5′-*O*-(Dimethoxytrityl)-*N* ^4^-isobutyryldeoxycytidine-3′-*H*-phosphonothioate monoester **1c** (triethylammonium salt, 39.2 mg, 50 μmol) in dry CD_3_CN (400 μL) was dried over MS 3A for 60 min in a reaction vessel. In another round-bottom flask, CMPT (52.0 mg, 200 μmol) was dissolved in CD_3_CN (200 μL) and dried over MS 3A. Then, DIC (15.5 μL, 100 μmol) and a solution of CMPT in CD_3_CN (100 μL) in the round-bottom flask were added successively to the reaction vessel. After 15 min, the solution was analyzed by ^31^P NMR spectroscopy (^31^P NMR spectrum S1). Subsequently, a solution of 3′-*O*-(*tert-*butyldiphenylsilyl)-*N* ^4^-isobutyrylcytidine (**2**, 32.1 mg, 60 μmol) in CD_3_CN (100 μL) was added to the NMR sample tube and the reaction mixture was shaken using vortex stirring. After 15 min, the solution was analyzed by ^31^P NMR spectroscopy (^31^P NMR spectrum S2).

### General Procedure for the Solid-Phase Synthesis

A 5′-*O*-DMTr-thymidine loaded on a solid support via a succinyl linker (0.5 μmol) in a reaction vessel was treated with 3% DCA in dry CH_2_Cl_2_ (8 × 10 s, each 1 mL) and washed with dry CH_2_Cl_2_ (8 × 1 mL) and dry MeCN (8 × 1 mL). Subsequently, the solid support was dried in vacuo for 10 min. To the reaction vessel, the deoxynucleoside 3′-*H*-phosphonothioate monomer **1c** (40 μmol, 31.2 mg), **1a** (40 μmol, 34.4 mg), **1t** (40 μmol, 29.0 mg), or **1g** (40 μmol, 40.6 mg) was added. Then, condensation reaction was conducted. The solid support was washed with dry MeCN (8 × 1 mL) and dry CH_2_Cl_2_ (8 × 1 mL), and the detritylation reaction was conducted by treating with 3% DCA in dry CH_2_Cl_2_ (8 × 10 s, each 1 mL). The solid support was then washed with dry CH_2_Cl_2_ (8 × 1 mL) and dried in vacuo for 5 min. The *H*-phosphonate and/or *H*-phosphonothioate diester internucleotidic linkage was sulfurized via treatment with a solution of Et_3_N (0.1 M, 50 μmol, 6.90 μL) and S_8_ (0.3 M, 150 μmol, 4.90 mg) in dry CH_2_Cl_2_ (500 μL) for 90 min. The solid support was washed with dry CH_2_Cl_2_ (8 × 1 mL), dry MeCN (4 × 1 mL), and EtOH (4 × 1 mL) and then treated with concentrated aqueous NH_3_–EtOH (3:1, v/v; 5 mL) at 55 °C for 18 h, filtered, and washed with EtOH (3 × 1 mL). The filtrate and washing were combined and concentrated under reduced pressure. The residue was analyzed by RP-HPLC with a linear gradient of 0%–30% MeCN for 30 min in 0.1 M triethylammonium acetate buffer (pH 7.0) at 30 °C and a flow rate of 0.5 mL/min using a C_18_ column (5 μm, 100 Å, 3.9 × 150 mm).

### General Procedure for the Investigation for the Formation of an *H*-Phosphonate Diester from an *H*-Phosphonothioate Monomer

#### Solid-Phase Synthesis of Dinucleoside Phosphorothioates (Table 2, Entries 1–3)

A 5′-*O*-DMTr-thymidine loaded on lcaa-CPG (entry 1), HCP (entry 2), or NittoPhase HL (entry 3) via a succinyl linker (0.5 μmol) in a reaction vessel was detritylated following the general procedure described above and washed with dry CH_2_Cl_2_ (4 × 1 mL for entry 1 and 2; 8 × 1 mL for entry 3) and dry MeCN (4 × 1 mL for entry 1 and 2; 8 × 1 mL for entry 3). Subsequently, the solid support was dried in vacuo for 10 min. To the reaction vessel, deoxycytidine 3′-*H*-phosphonothioate monomer **1c** (20 μmol, 15.6 mg) was added. Then, solutions of DIC (40 μmol, 6.19 μL) in dry MeCN (100 μL) and CMPT (40 μmol, 10.4 mg) in dry MeCN (100 μL) were successively added under Ar, and the reaction vessel was stirred slowly for 15 min. Then, detritylation, sulfurization, deprotection on nucleobases, and release from the solid support were conducted following the general procedure described above.

#### Solid-Phase Synthesis of Dinucleoside Phosphorothioates (Table 2, Entries 4–8)

A 5′-*O*-DMTr-thymidine loaded on NittoPhase HL via a succinyl linker (0.5 μmol) in a reaction vessel was detritylated and washed following the general procedure described above. Subsequently, the sample was dried in vacuo for 10 min. To the round-bottom flask, deoxynucleoside 3′-*H*-phosphonothioate monomer **1c** (40 μmol, 31.2 mg in entry 4), **1a** (40 μmol, 34.4 mg in entry 5), **1t** (40 μmol, 29.0 mg in entry 6), or **1g** (40 μmol, 40.6 mg in entries 7 and 8) was added. Then, solutions of DIC (80 μmol, 12.4 μL) in dry MeCN (200 μL) and CMPT (80 μmol, 20.8 mg) in dry MeCN (200 μL) were successively added under Ar, and the round-bottom flask was stirred slowly for 15 min. After that, the solution (200 μL) in the round-bottom flask was added to the reaction vessel and the reaction vessel was stirred slowly for 15 min. Then, detritylation, sulfurization, deprotection on nucleobases, and release from the solid support were conducted following the general procedure described above.

HRMS (ESI/Q-TOF) *m*/*z*: [M – H]^−^ calcd for C_19_H_25_N_5_O_10_PS^–^ (**11ct**), 546.1065; found, 546.1063. [M – H]^−^ calcd for C_20_H_25_N_7_O_9_PS^–^ (**11at**), 570.1178; found, 570.1178. [M – H]^−^ calcd for C_20_H_25_N_7_O_10_PS^–^ (**11gt**), 586.1127; found, 586.1125. [M – H]^−^ calcd for C_20_H_26_N_4_O_11_PS^–^ (**11tt**), 561.1062; found, 561.1060.

### General Procedure for the Investigation for the Formation of an *H*-Phosphonothioate Diester from an *H*-Phosphonothioate Monomer

#### Solid-Phase Synthesis of Dinucleoside Phosphorodithioates (Table 3, Entry 1)

A 5′-*O*-DMTr-thymidine loaded on NittoPhaese HL via a succinyl linker (0.5 μmol) in a reaction vessel was detritylated and washed following the general procedure described above. Subsequently, it was dried in vacuo for 10 min. To the reaction vessel were added deoxyadenosine 3′-*H*-phosphonothioate monomer **1a** (20 μmol, 17.2 mg) and a condensing reagent, 3-nitro-1,2,4-triazol-1-yl-tris­(pyrrolidin-1-yl)­phosphonium hexafluorophosphate (PyNTP) (50 μmol, 25.0 mg). Then, a solution of 20% v/v quinoline in dry MeCN (200 μL) was added under Ar, and the reaction vessel was stirred slowly for 3 min. Then, detritylation, sulfurization, deprotection on nucleobases, and release from the solid support were conducted following the general procedure described above.

#### Solid-Phase Synthesis of Dinucleoside Phosphorodithioates (Table 3, Entries 2–8)

A 5′-*O*-DMTr-thymidine loaded on NittoPhase HL via a succinyl linker (0.5 μmol) in a reaction vessel was detritylated and washed following the general procedure described above. Subsequently, it was dried in vacuo for 10 min. To the reaction vessel, deoxynucleoside 3′-*H*-phosphonothioate monomer **1a** (20 μmol, 17.2 mg in entries 2–5), **1c** (20 μmol, 15.6 mg in entry 6), **1g** (20 μmol, 20.3 mg in entry 7), or **1t** (20 μmol, 14.5 mg in entry 8) was added. Then, 20% v/v pyridine in dry MeCN (200 μL) in entries 2–4 or 10% v/v pyridine in dry MeCN (200 μL) in entries 5–8 and DPCP (30 μmol, 6.20 μL) in entry 2, DECP (30 μmol, 4.30 μL) in entry 3, or DICP (30 μmol, 5.40 μL) in entries 4–8 were successively added under Ar, and the reaction vessel was stirred slowly for 3 min in entries 2–4 or 5 min in entries 5–8. Then, detritylation, sulfurization, deprotection on nucleobases, and release from the solid support were conducted following the general procedure described above.

HRMS (ESI/Q-TOF) *m*/*z*: [M – H]^−^ calcd for C_19_H_25_N_5_O_9_PS_2_^–^ (**12ct**), 562.0837; found, 562.0835. [M – H]^−^ calcd for C_20_H_25_N_7_O_8_PS_2_^–^ (**12at**), 586.0949; found, 586.0945. [M – H]^−^ calcd for C_20_H_25_N_7_O_9_PS_2_^–^ (**12gt**), 602.0898; found, 602.0896. [M – H]^−^ calcd for C_20_H_26_N_4_O_10_PS_2_^–^ (**12tt**), 577.0833; found, 577.0834.

### Synthesis of the PS_2_/PS Chimeric Pentamer and Dodecamer ODNs

A 5′-*O*-DMTr-thymidine loaded on NittoPhase HL via a succinyl linker (0.5 μmol) in a reaction vessel was detritylated and washed following the procedure described above. Subsequently, it was dried in vacuo for 10 min. Chain elongations were conducted by repeating steps (a) or (b) and (c) 4 times or 11 times.

(a) Formation of the *H*-phosphonate diester step: To introduce an *H*-phosphonate diester linkage, to the round-bottom flask, the deoxynucleoside 3′-*H*-phosphonothioate monomer **1c** (40 μmol, 31.2 mg), **1a** (40 μmol, 34.4 mg), **1g** (40 μmol, 40.6 mg), or **1t** (40 μmol, 29.0 mg) was added. Then, solutions of DIC (80 μmol, 12.4 μL) in dry MeCN (200 μL) and CMPT (80 μmol, 20.8 mg) in dry MeCN (200 μL) were successively added under Ar, and the round-bottom flask was stirred slowly for 15 min. Subsequently, the solution (200 μL) in the round-bottom flask was added to the reaction vessel, and the reaction vessel was stirred slowly for 15 min. The solid support was washed with dry MeCN (8 × 1 mL) and dry CH_2_Cl_2_ (8 × 1 mL).
(b) Formation of *H*-phosphonothioate diester step: To introduce an *H*-phosphonothioate diester linkage, to the reaction vessel, the deoxynucleoside 3′-*H*-phosphonothioate monomer **1c** (20 μmol, 15.6 mg), **1a** (20 μmol, 17.2 mg), **1g** (20 μmol, 20.3 mg), or **1t** (20 μmol, 14.5 mg) was added. Then, 10% v/v pyridine in dry MeCN (200 μL) and DICP (30 μmol, 5.4 μL) were successively added under Ar, and the reaction vessel was stirred slowly for 5 min. The solid support was washed with dry MeCN (8 × 1 mL) and dry CH_2_Cl_2_ (8 × 1 mL).
(c) Detritylation step: To the reaction vessel, 3% DCA in dry CH_2_Cl_2_ (8 × 10 s, each 1 mL) was added and the solid support was washed with dry CH_2_Cl_2_ (8 × 1 mL) and dry MeCN (8 × 1 mL). After that, it was dried in vacuo for 10 min. In the case of the last step, after treatment with 3% DCA in dry CH_2_Cl_2_ (8 × 10 s, each 1 mL), the reaction vessel was washed with dry CH_2_Cl_2_ (8 × 1 mL) and dried in vacuo for 5 min.

After the designed length was achieved, detritylation, sulfurization, deprotection on nucleobases, and release from the solid support were conducted following the general procedure described above. The residue was analyzed and then purified by RP-UPLC with a linear gradient of 1%–35% solution B in a buffer containing solution A at 45 °C for 10 min at a flow rate of 0.6 mL/min using a C_18_ Column ACQUITY Premier 1.7 μm (130 Å, 2.1 × 50 mm, Waters) for the analysis of the pentamer. RP-UPLC was performed with a linear gradient of 5%–25% solution D in a buffer containing solution C at 60 °C for 15 min at a flow rate of 0.6 mL/min using a C_18_ Column ACQUITY Premier 1.7 μm (130 Å, 2.1 × 50 mm, Waters) for the analysis of the dodecamer. The purification was conducted with three-fiftieths of the crude mixture.

Solution A: 50 mM hexafluoroisopropanol (HFIP), 5 mM hexylamine (HA) aq. Solution B: Solution A–MeCN (1:1, v/v). Solution C: 100 mM HFIP and 8 mM Et_3_N aq. Solution D: Solution C–MeCN (1:1, v/v).

Isolated yields 32% (d­(T_PS_C_PS2_A_PS_G_PS2_T) (**13**)); 11% (d­(C_PS2_A_PS_G_PS2_T_PS_C_PS_A_PS2_G_PS_T_PS2_C_PS2_A_PS_G_PS2_T) (**14**)) HRMS (ESI/Q-TOF) *m*/*z*: [M – 2H]^2–^ calcd for C_49_H_61_N_17_O_23_P_4_S_6_^2–^ (**13**), 785.5706; found, 785.5664. [M–4H]^4–^ calcd for C_117_H_144_N_45_O_53_P_11_S_17_^4–^ (**14**), 978.0594; found, 978.0570.

### Synthesis of the PSN/PN Chimeric Pentamer ODN

A 5′-*O*-DMTr-thymidine loaded on NittoPhase HL via a succinyl linker (0.5 μmol) in a reaction vessel was detritylated and washed following the general procedure described above. After that, the sample was dried in vacuo for 10 min. Chain elongations were conducted by repeating steps (a) or (b) and (c) 4 times, as described in the synthesis of PS_2_/PS chimeric ODNs.

After the designed length was achieved, the resultant internucleotidic linkages were oxidatively aminated with a solution of CCl_4_–MeCN–morpholine (4.5:4.5:1, v/v/v) (500 μL) for 5 min. The solid support was washed with dry MeCN (8 × 1 mL), dry CH_2_Cl_2_ (8 × 1 mL), and EtOH (4 × 1 mL). The solid support was treated with concentrated aqueous NH_3_–EtOH (3:1, v/v; 5 mL) at 55 °C for 18 h, filtered, and washed with EtOH (3 × 1 mL). The filtrate and washing were combined and concentrated under reduced pressure. The residue was analyzed and then purified by RP-UPLC with a linear gradient of 20%–70% solution F in a buffer containing solution E at 45 °C for 10 min at a flow rate of 0.6 mL/min using a C_18_ Column ACQUITY Premier 1.7 μm (130 Å, 2.1 × 50 mm, Waters). The purification was conducted with three-fiftieths of the crude mixture.

Solution E: 50 mM HFIP and 5 mM Et_3_N aq. Solution F: solution C–MeCN (1:1, v/v).

Isolated yields 16% (d­(T_PN_C_PSN_A_PN_G_PSN_T) (**15**)) HRMS (ESI/Q-TOF) *m*/*z*: [M + H]^+^ calcd for C_65_H_92_N_21_O_27_P_4_S_2_^+^ (**15**), 1786.4858; found, 1786.4720.

## Supplementary Material



## Data Availability

The data underlying this study are available in the published article and its online Supporting Information.

## Notes

Experimental details, HPLC profiles, and copies of ^1^H, ^13^C, ^31^P NMR, COSY, HSQC, and HMBC spectra of a new compound (PDF)
