# Supplementary material for: Synthesis of P‑Modified Chimeric Oligonucleotides via Chemoselective Condensation of Nucleoside 3′‑H‑Phosphonothioate
Source: J Org Chem. 2025 Aug 25;90(35):12346–58. doi: 10.1021/acs.joc.5c01498 (PMC12418309; doi:10.1021/acs.joc.5c01498)
Supplement: Supplementary file 1 [file jo5c01498_si_001.pdf]

Synthesis of *P*-modified chimeric oligonucleotides via chemoselective condensation of nucleoside 3′-*H*-phosphonothioate

Harumi Okutsu, Yuhei Takahashi, Tomomi Shiraishi, Utako Kashio, Kazuki Sato and Takeshi Wada*

Department of Medicinal and Life Sciences, Faculty of Pharmaceutical Sciences, Tokyo University of Science, 6-3-1 Niijuku, Katsushika-ku, Tokyo 125-8585, Japan.

Supporting Information

Table of contents

General information S3

Synthesis of the *H*-phosphonothioate monomers S4

Investigation of condensing reagents for the formation *H*-phosphonate diester from *H*-phosphonothioate monomer S17

Screening of carbodiimide derivatives S22

$^{31}P\{^1H\}$ NMR spectra of Scheme 2 .... S25

$^{31}P\{^1H\}$ NMR spectra of Scheme 3 .... S28

Evaluation of *S*-selectivity by using *N,N'*-diisopropylcarbodiimide (DIC) S30

Plausible mechanism for the formation of an *H*-phosphonate diester S35

RP-HPLC profiles of $N_{PS}T$ dimer S37

RP-HPLC profiles of $N_{PS2}T$ dimer S41

Desulfurization by PyNTP in the solid-phase synthesis S45

RP-HPLC profiles of purified PS_2/PS chimeric pentamer (13) S47

RP-HPLC profiles of purified PS_2/PS chimeric dodecamer (14) S47

Analysis of PS_2/PS chimeric pentamer and dodecamer by LC-MS/MS S48

Investigation of synthesis of PSN and PN dimers S56

RP-UPLC profiles of crude PSN/PN chimeric pentamer (15) S60

RP-HPLC profiles of purified PSN/PN chimeric pentamer (15) S61

Analysis of PSN/PN chimeric pentamer by LC-MS/MS S61

Reference S64

General information

All reactions reported herein were performed under an argon atmosphere. Organic solvents were dried using appropriate methods.

Compound **S1a**, **S1c**, **S1t** were commercial grade (Chem Genes corporation).

^{1}H NMR spectra were recorded in $CDCl_3$ and CD_3CN using tetramethylsilane (δ 0.00) as an internal standard at 400 MHz (JEOL JNM 400) or 500 MHz (Bruker Avance NEO 500). ^{13}C NMR spectra were recorded in CD_3CN using CD_3CN (δ 1.32) as an internal standard at 126 MHz on a 500 MHz Bruker Avance NEO 500. ^{31}P NMR spectra were recorded in CD_3CN and Pyridine-*d_5* using 85% H_3PO_4 (δ 0.0) as an external standard at 161 MHz (on a 400 MHz JEOL JNM 400), 243 MHz (on a 600 MHz Bruker Avance 600), or 202 MHz (on a 500 MHz Bruker Avance NEO 500). gCOSY, gHSQC, and gHMBC spectra were recorded on a 500 MHz Bruker Avance NEO 500.

Structural assignments were made with additional information from gCOSY, gHSQC, and gHMBC experiments

Purification by silica gel column chromatography was performed by automated flash chromatography using a Yamazen UNIVERSAL Premium column (30 µm, 60 Å) on an automated flash chromatography system W-prep 2XY. Thin-layer chromatography (TLC) was performed on TLC plates Silica gel 60 F_{254} (Merck, No. 5715).

The synthesized dimers were analyzed by RP-HPLC (JASCO chromNAV system equipped with a JASCO PU-2080i plus pump and a JASCO UV-2075 plus as a UV/VIS detector) using a C_{18} column (Delta pak 5 µm C_{18} column, 100 Å, 3.9 mm × 150 mm, Waters). The synthesized pentamers and dodecamers were analyzed and purified by LC-MS (ACQUITY Premier, Waters) using a C_{18} column (ACQUITY™ Premier BEH C_{18} 1.7 µm, 2.1 × 50 mm, 130 Å, Waters).

Isolated yields of oligomers were calculated by UV absorbance UV spectrum (260 nm) using a UV spectrometer (SHIMADZU, UV-1900i). Molar extinction coefficients (ε) at 260 nm were estimated as 48100 $M^{-1}cm^{-1}$ for **13** and **15**, and 118100 $M^{-1}cm^{-1}$ for **14**.

The LC-MS/MS analysis was conducted by CONFIRM Sequence App 1.4.0.13 and SELECT SERIES Cyclic IMS (Waters). Precursor and fragment ions mass tolerance is ±10 ppm.

Synthesis of the *H*-phosphonothioate monomers

Scheme S1

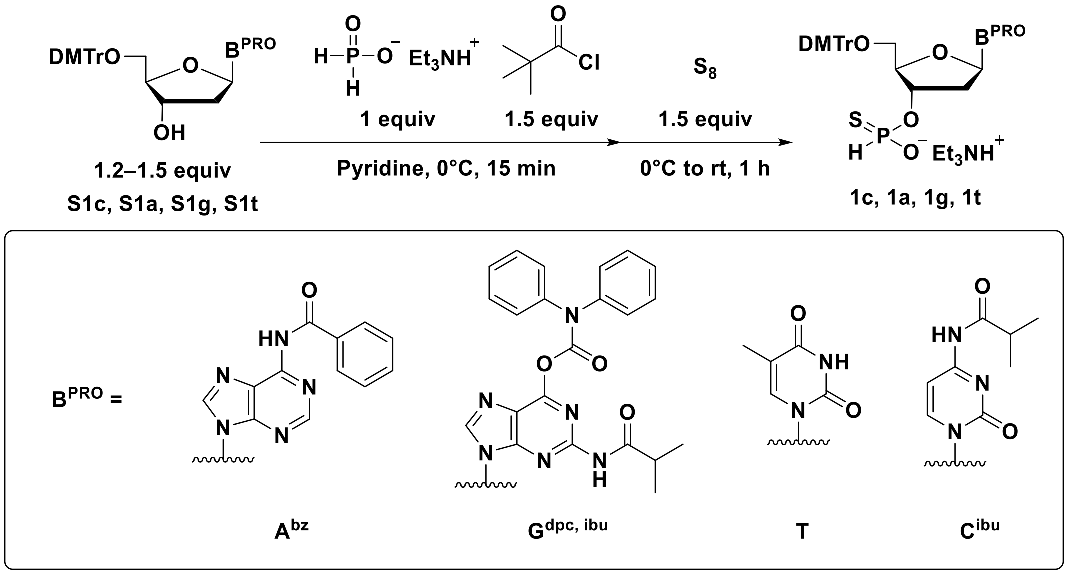

Nucleoside **S1a** (1.97 g, 3.0 mmol, 1.5 equiv), **S1t** (1.66 g, 3.0 mmol, 1.5 equiv), **S1c** (2.70 g, 4.5 mmol, 1.5 equiv), or **S1g** (2.00 g, 2.4 mmol, 1.2 equiv) in a round-bottom flask was dried by repeated coevaporation with dry pyridine. In a different flask, triethylamine (ca. 1 mL for **1t**, **1c**, and **1g**; ca. 0.5 mL for **1a**) was added to 50% phosphinic acid aqueous solution (0.22 mL, 2 mmol, 1.0 equiv for **1a**, **1t**, or **1g**; 0.33 mL, 3.0 mmol, 1.0 equiv for **1c**) and the solution was dried by coevaporation with dry pyridine. Thereafter, the nucleoside and triethylammonium phosphinate were dissolved in the same solvent (15 mL for **1a**, **1t**, or **1g**; 22.5 mL for **1c**). Pivaloyl chloride (0.37 mL, 3 mmol, 1.5 equiv for **1a**, **1t**, or **1g**; 0.56 mL, 4.6 mmol, 1.5 equiv for **1c**) was added to the solution at 0°C while stirring for 15 min. Subsequently, elemental sulfur (96 mg, 3 mmol, 1.5 equiv for **1a**, **1t**, or **1g**; 144.0 mg, 4.5 mmol, 1.5 equiv for **1c**) was added and the solution was warmed to rt. Stirring was continued for designated time (1 h for **1a**, **1c**, and **1g**; 30 min for **1t**), and then the mixture was diluted with CH_2Cl_2 (50 mL) and washed with 1.0 M triethylammonium bicarbonate (TEAB) buffers (3 × 25 mL). The aqueous layers were combined and back-extracted with CH_2Cl_2 (1 × 20 mL). The combined organic layers were dried over Na_2SO_4, filtered, and concentrated under reduced pressure. The residue was coevaporated with toluene and purified twice by silica gel column chromatography using EtOAc−MeOH and CH_2Cl_2−MeOH with Et_3N (1% for the first time, 0% for the second time) as the eluent. The fractions containing **1a**, **1t**, **1c**, or **1g** were collected and concentrated under reduced pressure to yield **1a**, **1t**, **1c**, or **1g**.

Triethylammonium 5′-*O*-dimethoxytrityl-*N*^{6}-benzoydeoxyladenosine 3′-*H*-phosphonothioate as a mixture of (*Sp*) and (*Rp*)-diastereomer (1a)

The crude mixture containing **1a** was purified twice by automated flash chromatography on a Yamazen UNIVERSAL premium column (40 g of neutral silica gel, 3 × 20 cm) using EtOAc−MeOH (100:0−95:5, v/v) and then CH_2Cl_2−MeOH (92:8–80:20, v/v) with Et_3N (1% for the first time, 0% for the second time) as the eluent. Then, the fractions containing **1a** were collected and concentrated under reduced pressure. **1a** was

obtained as colorless foam (1.23 g, 1.49 mmol, 75% yield). The ^{1}H NMR spectrum corresponded to the literature data[1] (**Figure S2**).

Triethylammonium 5′-*O*-dimethoxytrityl-thymidine 3′-*H*-phosphonothioate as a mixture of (*Sp*) and (*Rp*)-diastereomer (1t)

The crude mixture containing **1t** was purified twice by automated flash chromatography on a Yamazen UNIVERSAL premium column (40 g of neutral silica gel, 3 × 20 cm) using EtOAc−MeOH (100:0−95:5, v/v) and then CH_2Cl_2−MeOH (92:8–80:20, v/v) with Et_3N (1% for the first time, 0% for the second time) as the eluent. Then, the fractions containing **1t** were collected and concentrated under reduced pressure and coevaporated with $CHCl_3$. **1t** was obtained as colorless foam (0.90 g, 1.25 mmol, 63% yield). The ^{1}H NMR spectrum corresponded to the literature data[1] (**Figure S3**).

Triethylammonium 5′-*O*-dimethoxytrityl-*N*^{4}-isobutyryldeoxycytidine 3′-*H*-phosphonothioate as a mixture of (*Sp*) and (*Rp*)-diastereomer (1c)

The crude mixture containing **1c** was purified twice by automated flash chromatography on a Yamazen UNIVERSAL premium column (40 g of neutral silica gel, 3 × 20 cm) using EtOAc−MeOH (100:0−95:5, v/v) and then CH_2Cl_2−MeOH (92:8–80:20, v/v) with Et_3N (1% for the first time, 0% for the second time) as the eluent. Then, the fractions containing **1c** were collected and concentrated under reduced pressure. **1c** was obtained as colorless foam (2.24 g, 2.88 mmol, 96% yield). The ^{1}H NMR spectrum corresponded to the literature data[2] (**Figure S4**).

Triethylammonium 5′-*O*-dimethoxytrityl-*N*^{2}-isobutyryl-*O*^{6}-diphenylcarbamoyldeoxyguanosine 3′-*H*-phosphonothioate as a mixture of (*Sp*) and (*Rp*)-diastereomer (1g)

The crude mixture containing **1g** was purified twice by automated flash chromatography on a Yamazen UNIVERSAL premium column (40 g of neutral silica gel, 3 × 20 cm) using EtOAc−MeOH (100:0−95:5, v/v) and then CH_2Cl_2−MeOH (92:8–80:20, v/v) with Et_3N (1% for the first time, 0% for the second time) as the eluent. Then, the fractions containing **1g** were collected and concentrated under reduced pressure and coevaporated with $CHCl_3$. Compound **1g** was obtained as brown foam (1.02 g, 1.01 mmol, 51% yield). **^{1}H NMR** (CD_3CN, 400 MHz) δ 10.9 (br, 1H, $Et_3N^+\underline{H}$), 8.94−8.80 (m, 1H, N$\underline{H}$CO), 8.18 (s, 0.5H, H-8, one of diastereomers), 8.17 (s, 0.5H, H-8, one of diastereomers), 7.90 (d, *J* = 573 Hz, 0.5H, P-$\underline{H}$ one of diastereomers), 7.86 (d, *J* = 572 Hz, 0.5 H, P-H one of diastereomers), 7.55–7.44 (m, 3.5H, Ar), 7.39 (t, *J* = 7.6 Hz, 4H, Ar), 7.34–7.26 (m, 4H, Ar), 7.23–7.17 (m, 4H, Ar), 7.16−7.07 (m, 3H, Ar), 6.75−6.67 (m, 4H, Ar), 6.36–6.29 (m, 1H, H-1′), 5.46−5.33 (m, 1H, H-3′), 4.32–4.27 (m, 0.5H, H-4′, one of diastereomers), 4.23–4.20 (m, 0.5H, H-4′, one of diastereomers), 3.68, 3.68, 3.67, 3.67 (4×s, 6H, -OC$\underline{H}_3$), 3.41 (dd, *J* = 6.0, 10.4 Hz, 0.5H, H-5′, one of diastereomers), 3.35 (dd, *J* = 5.7, 10.5 Hz, 0.5H, H-5′, one of diastereomers), 3.23 (dd, *J* = 2.3, 10.5 Hz, 1H, H-5″), 3.19–3.10 (m, 1H, H-2′), 3.01 (q, *J* = 7.2 Hz, 6H, $(CH_3C\underline{H}_2)_3N$), 2.83−2.73 (m, 1H, isobutyryl-C$\underline{H}$(CH_3)$_2$), 2.65–2.52 (m, 1H, H-2″), 1.20 (t, *J* = 7.3 Hz, 9H, $(C\underline{H}_3CH_2)_3N$), 1.15–1.10 (m, 6H, isobutyryl-CH(C$\underline{H}_3$)$_2$); **^{13}C{^{1}H} NMR** (CD_3CN, 126 MHz) δ 176.0 (isobutyryl-$\underline{C}$O-), 159.5 (Ar), 156.7, 155.4 (C-4), 153.1, 153.1,

151.6, 145.9 (Ar), 144.9 (C-8), 143.0 (Ar), 137.1 (Ar), 137.0 (Ar), 136.9 (Ar), 136.7 (Ar), 136.7 (Ar), 131.0 (Ar), 131.0 (Ar), 130.9 (Ar), 130.3 (Ar), 130.2 (Ar), 129.0 (Ar), 129.0 (Ar), 128.6 (Ar), 128.3 (Ar), 127.7 (Ar), 122.1 (C-5, one of diastereomers), 122.0 (C-5, one of diastereomers), 113.9 (Ar), 86.9 (-$\underline{C}Ar_3$), 86.6 (d, $^3J_{C\text{-}P}$ = 6.0 Hz, C-4′, one of diastereomers), 86.1 (d, $^3J_{C\text{-}P}$ = 5.9 Hz, C-4′, one of diastereomers), 85.9 (C-1′, one of diastereomers), 85.7 (C-1′, one of diastereomers), 75.3 (C-3′, one of diastereomers), 74.7 (d, $^2J_{C\text{-}P}$ = 3.9 Hz, C-3′, one of diastereomers), 64.7 (C-5′, one of diastereomers), 64.5 (C-5′, one of diastereomers), 55.8 (-O$\underline{C}H_3$), 55.8 (-O$\underline{C}H_3$), 46.7 (($CH_3\underline{C}H_2)_3N$), 38.5 (C-2′, one of diastereomers), 38.1 (C-2′, one of diastereomers), 36.3 (isobutyryl-CO$\underline{C}$H-), 19.7 (-CH($\underline{C}H_3)_2$), 19.6 (-CH($\underline{C}H_3)_2$), 9.03 (($\underline{C}H_3CH_2)_3N$); **^{31}P{^{1}H} NMR** (CD_3CN, 162 MHz) δ 53.8 ($^1J_{P\text{-}H}$ = 573 Hz), 53.6 ($^1J_{P\text{-}H}$ = 572 Hz); **IR** (neat, cm^{-1}) 2970, 2316, 1739, 1586, 1506, 1446, 1382, 1301, 1248, 1174, 1030, 933, 827, 788, 755, 626, 583, 434, 422; **HRMS (ESI/Q-TOF)** *m/z*: [M−TEA− H]$^-$ Calcd for $C_{48}H_{46}N_6O_9PS^-$, 913.2790; Found 913.2770.

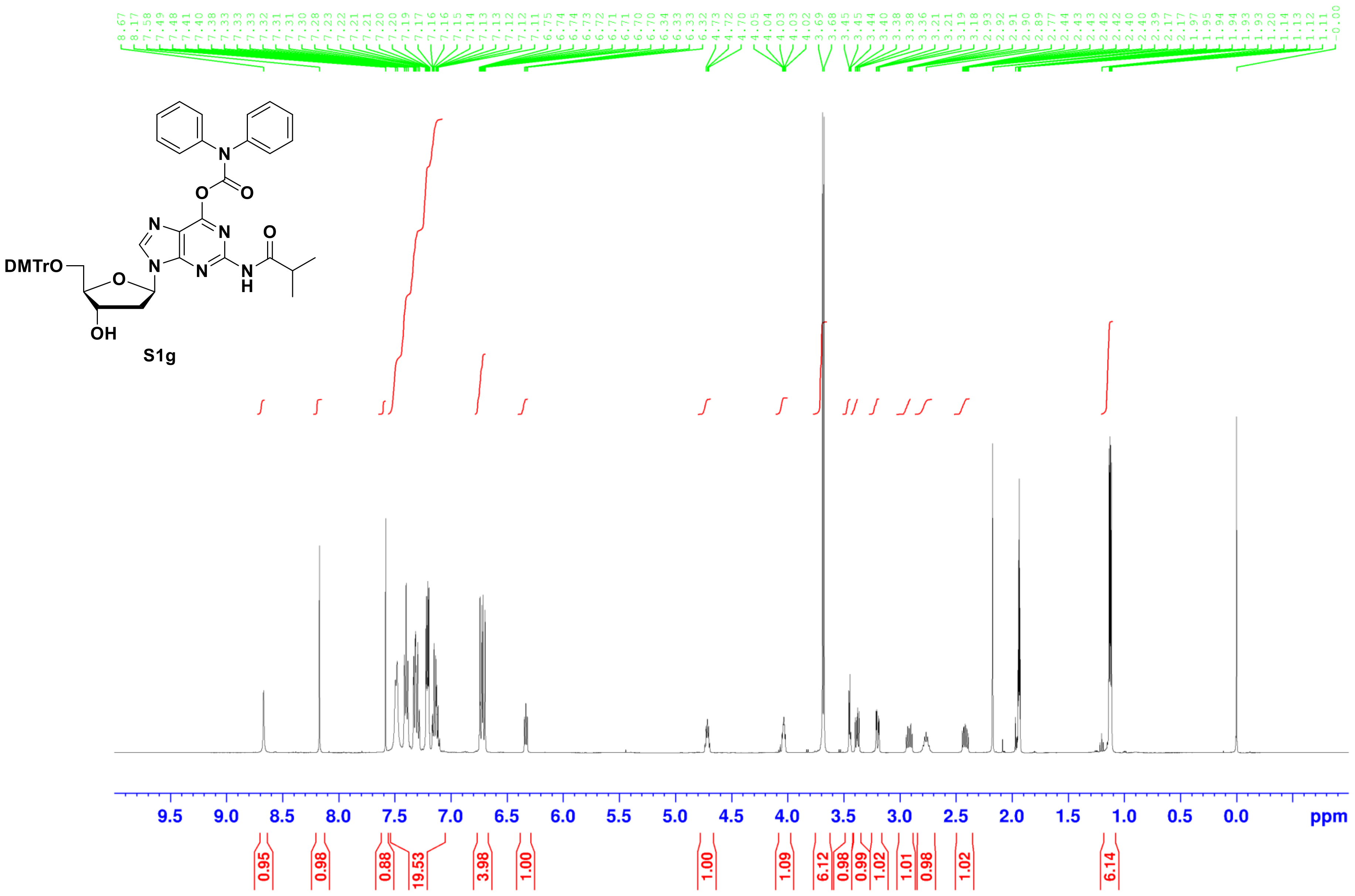

Figure S1 ^{1}H NMR spectrum of **S1g** (500 MHz, in CD_3CN)

NMR profiles of *H*-phosphonothioate monomer

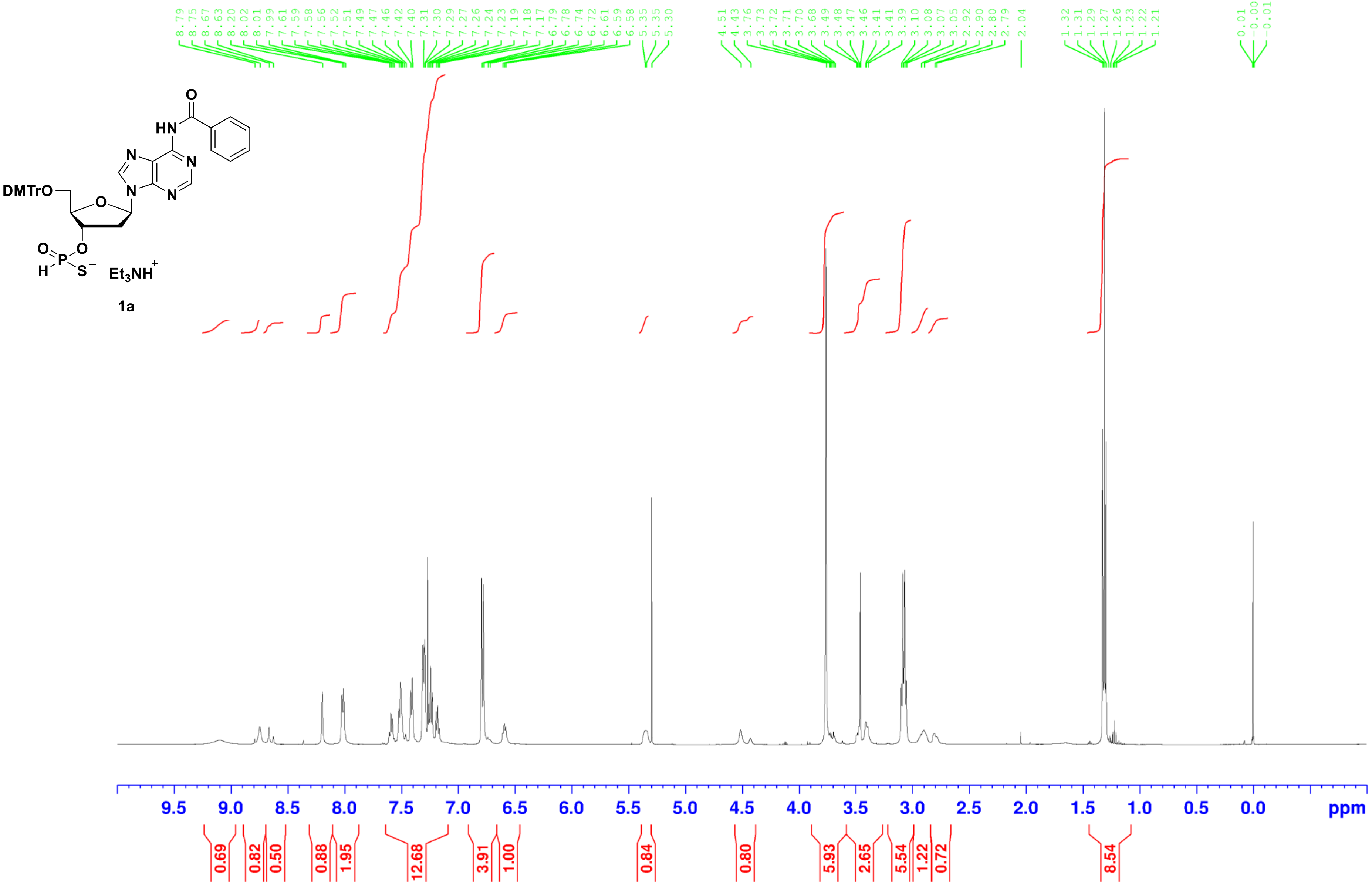

Figure S2 ^{1}H NMR spectrum of **1a** (500 MHz, in $CDCl_3$)

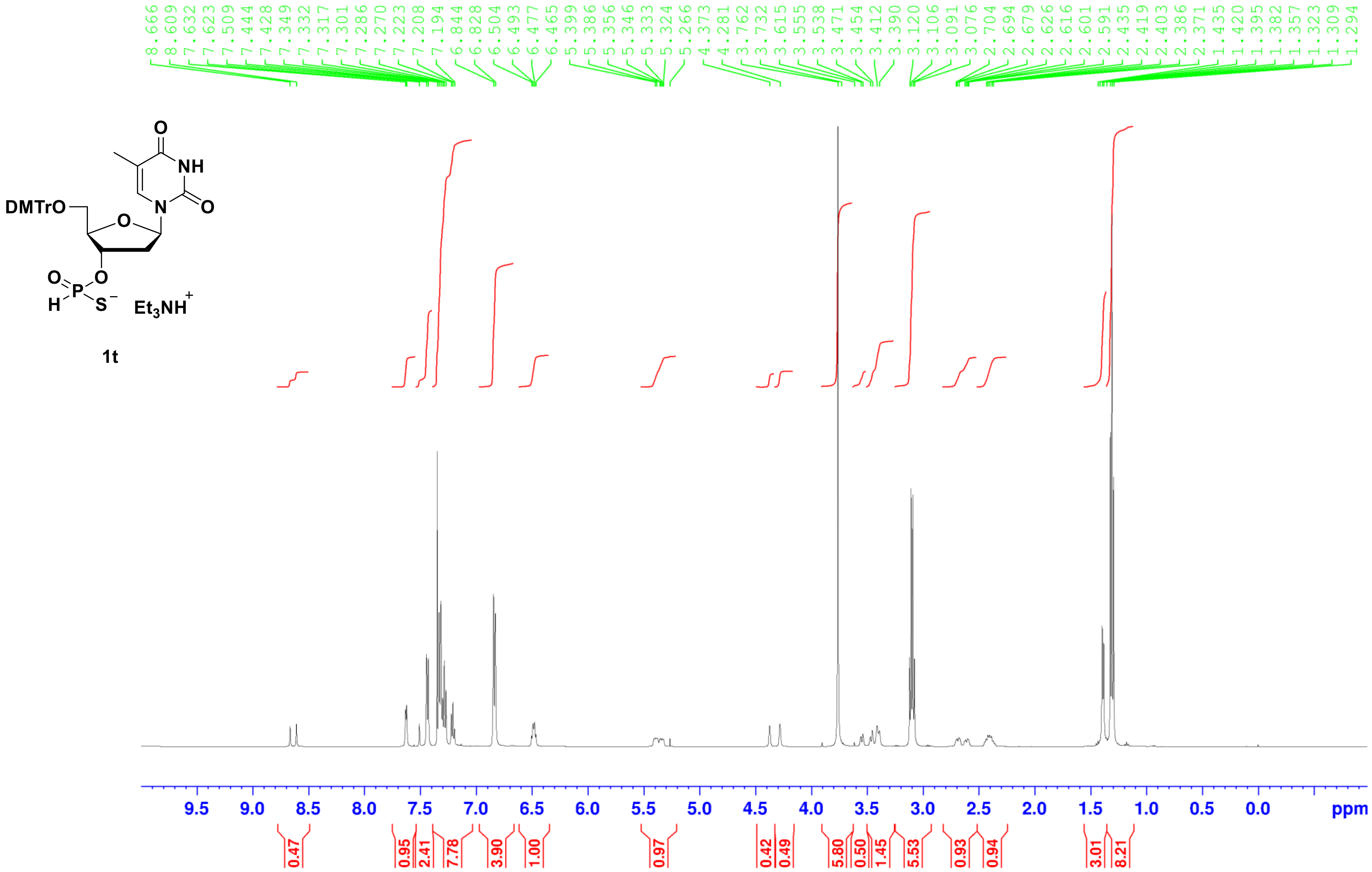

Figure S3 ^{1}H NMR spectrum of **1t** (500 MHz, in $CDCl_3$)

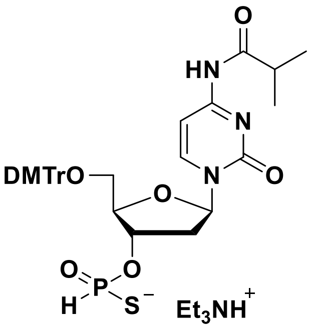

1c

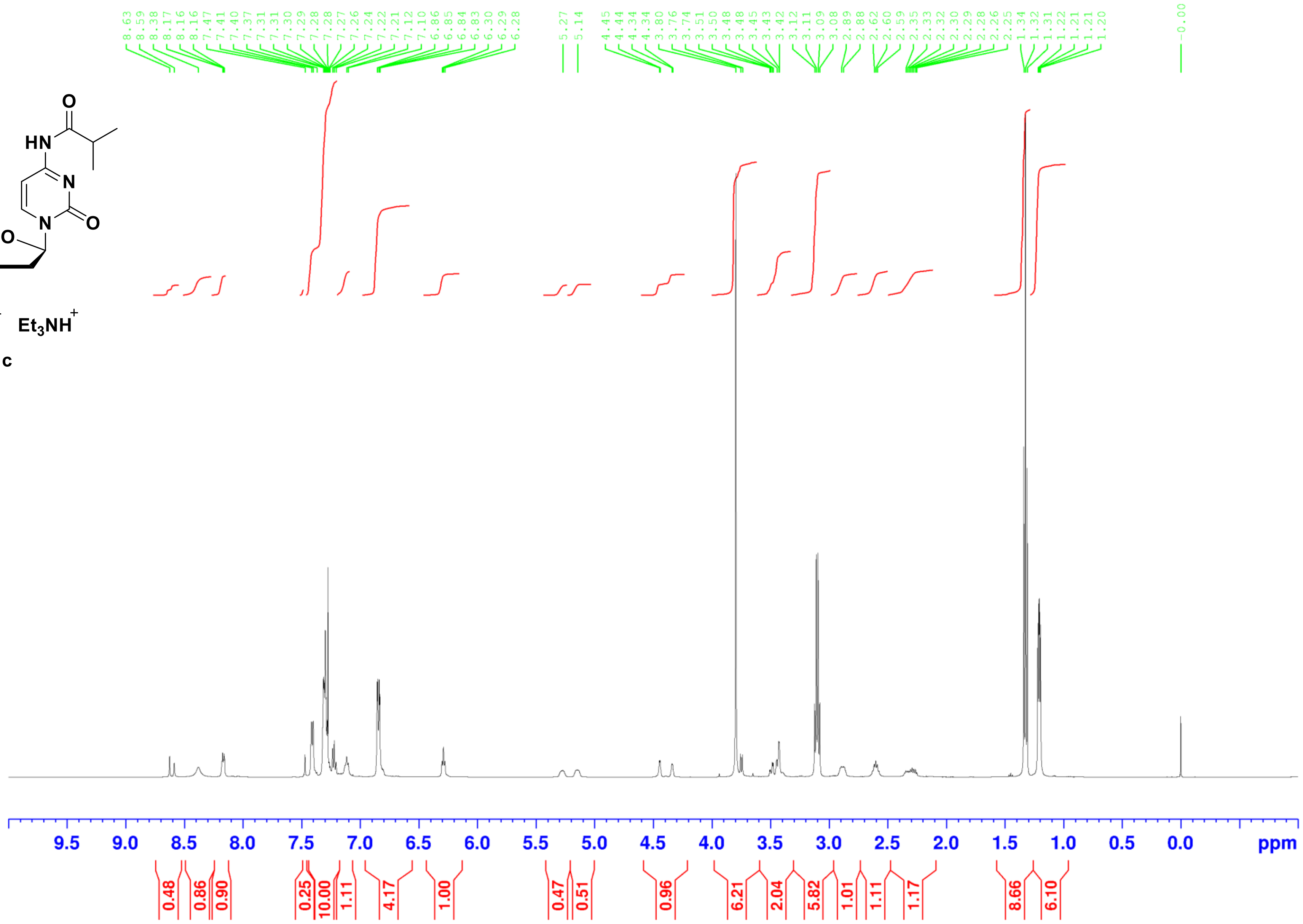

Figure S4 ^{1}H NMR spectrum of **1c** (500 MHz, in $CDCl_3$)

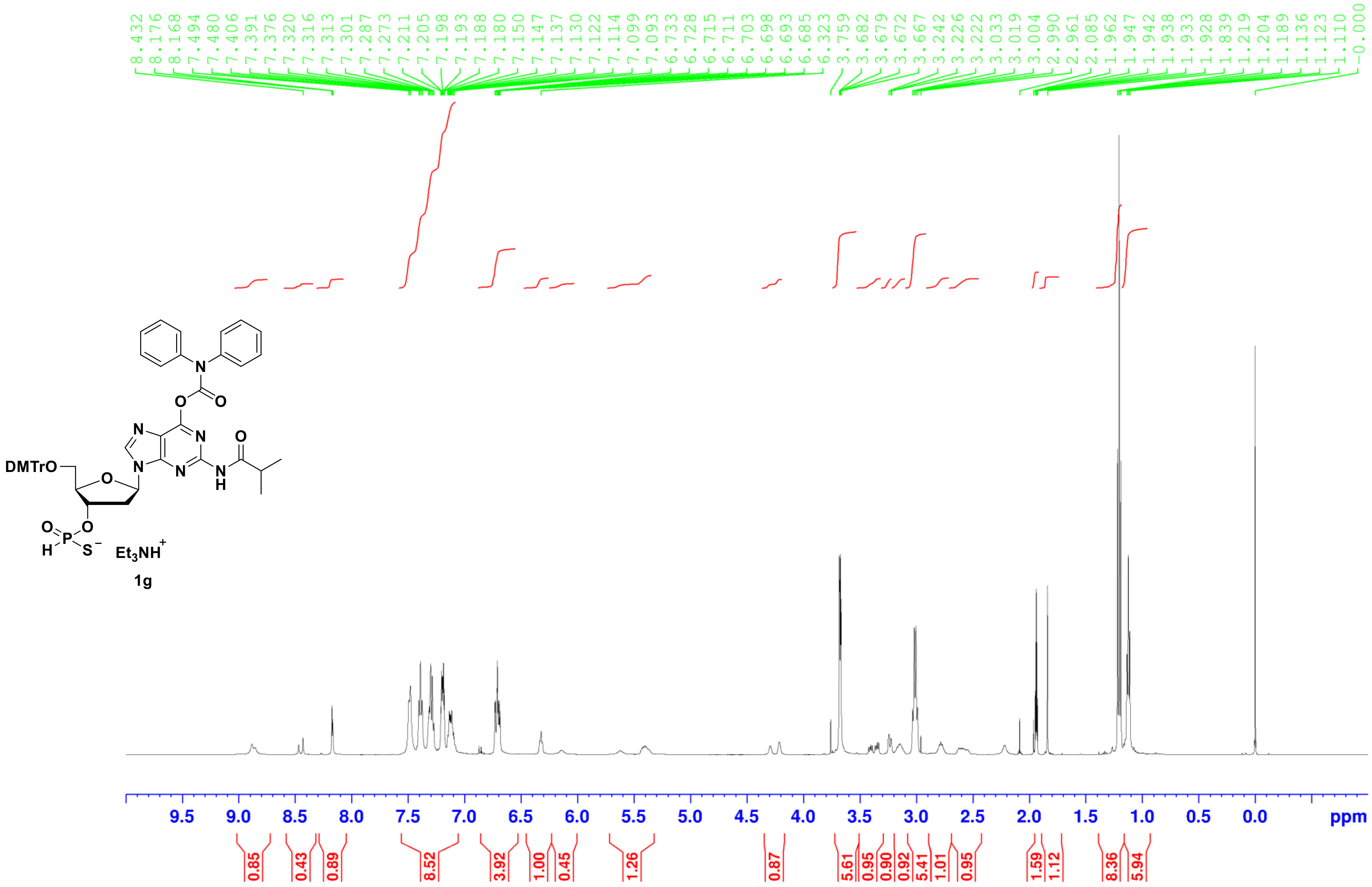

Figure S5 ^{1}H NMR spectrum of **1g** (500 MHz, in CD_3CN)

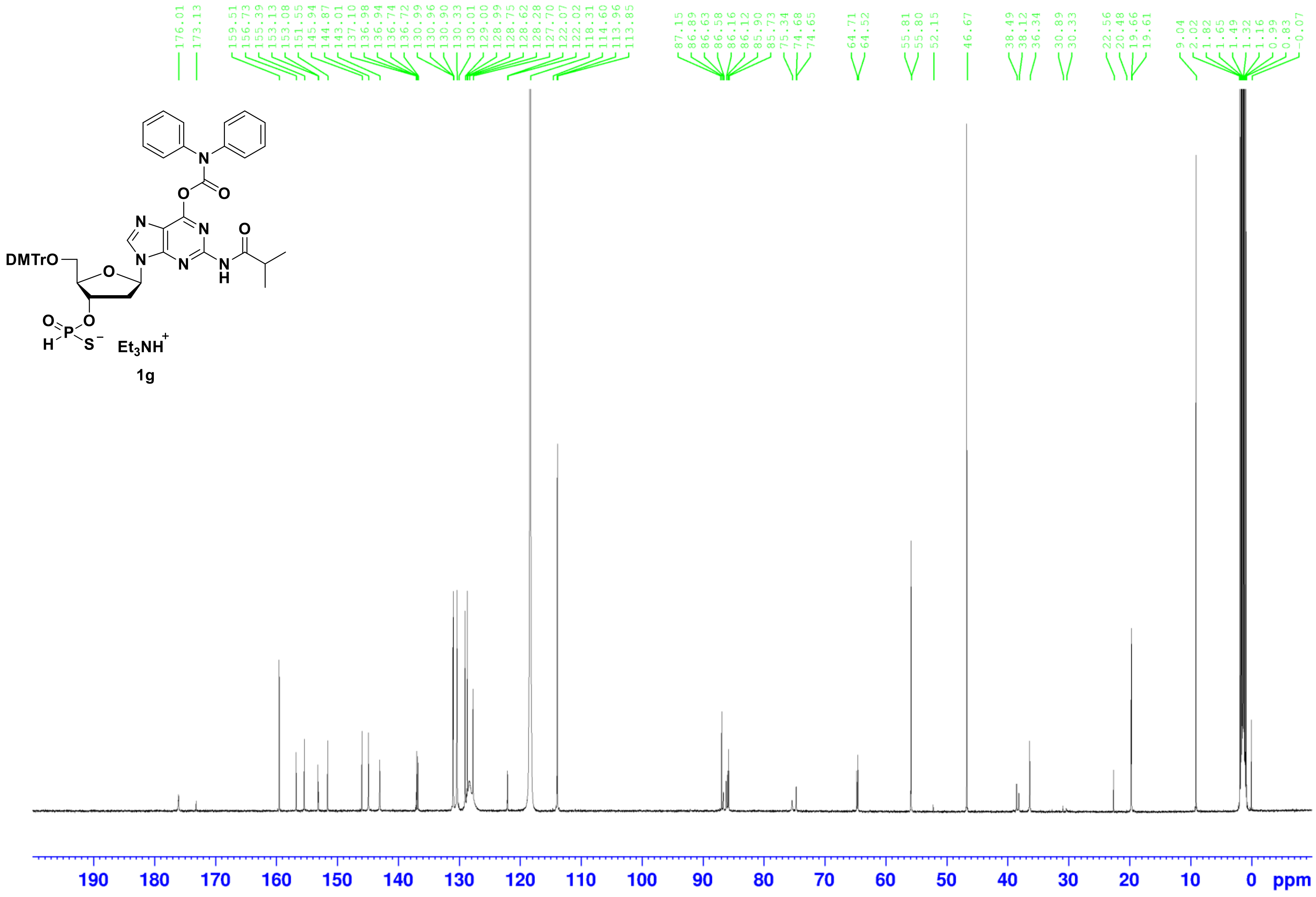

Figure S6 $^{13}C\{^{1}H\}$ NMR spectrum of **1g** (126 MHz, in CD_3CN)

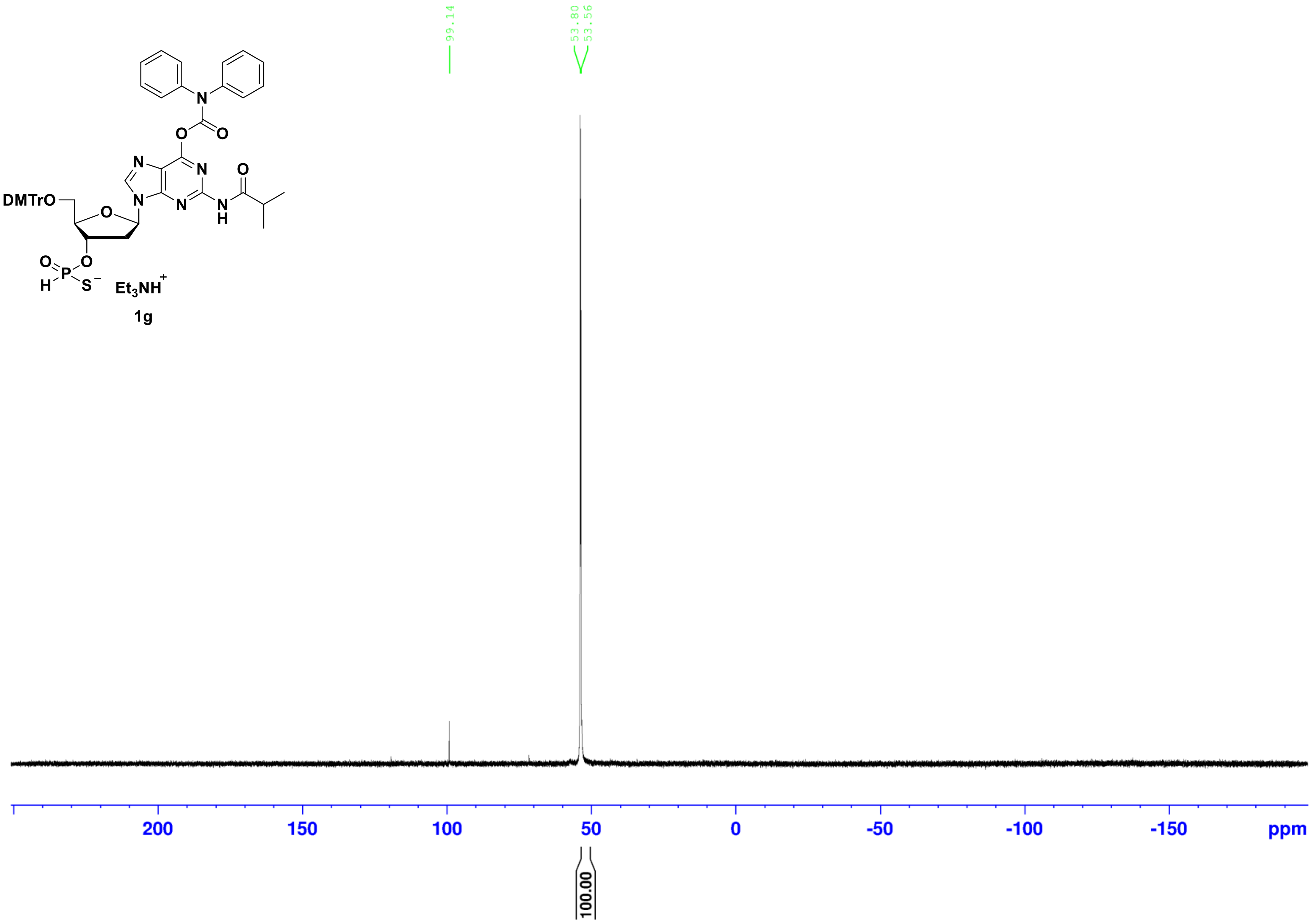

Figure S7 ^{31}P{^{1}H} NMR spectrum of **1g** (202 MHz, in CD_3CN)

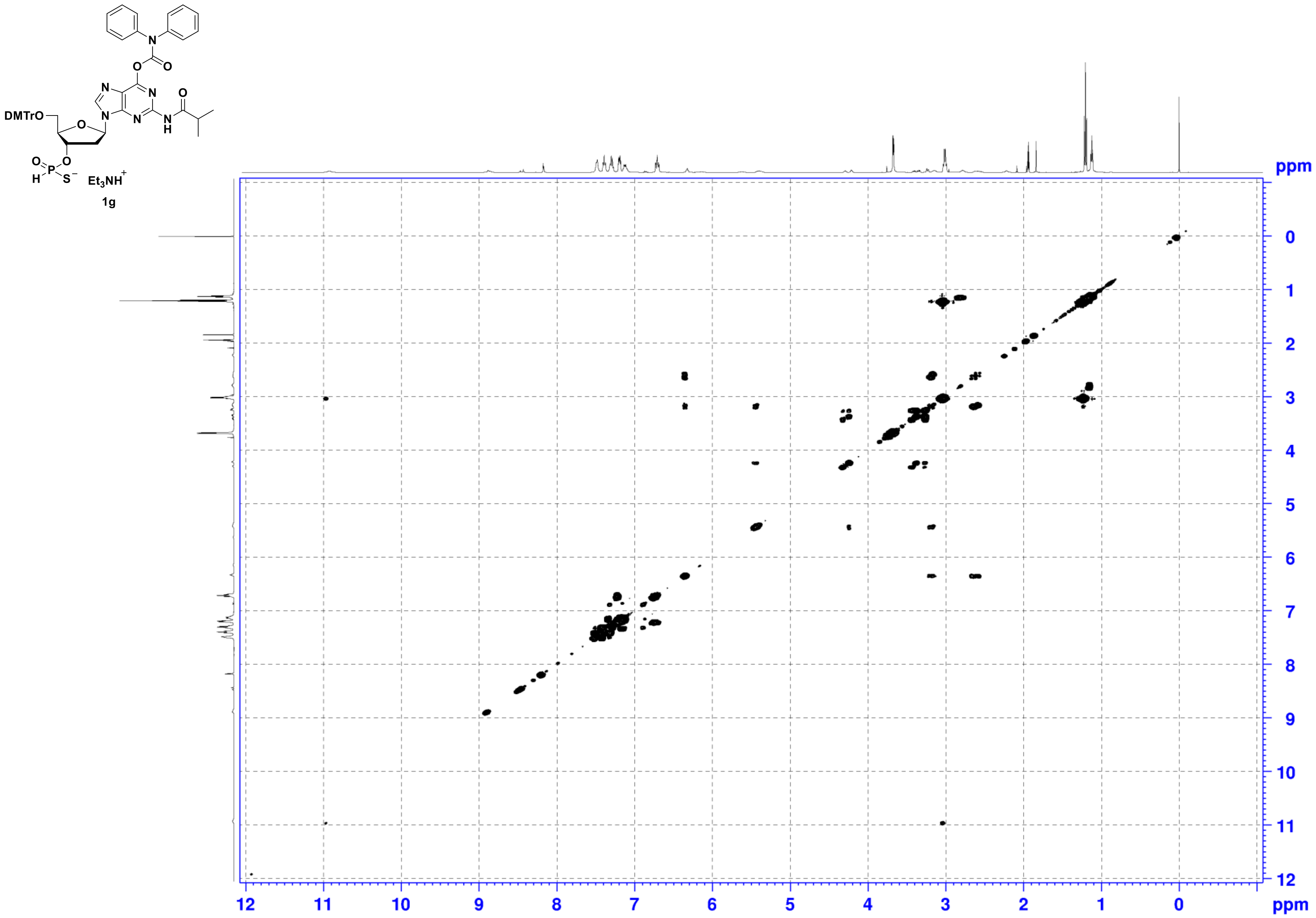

Figure S8 gCOSY spectrum of **1g** (500 MHz, in CD_3CN)

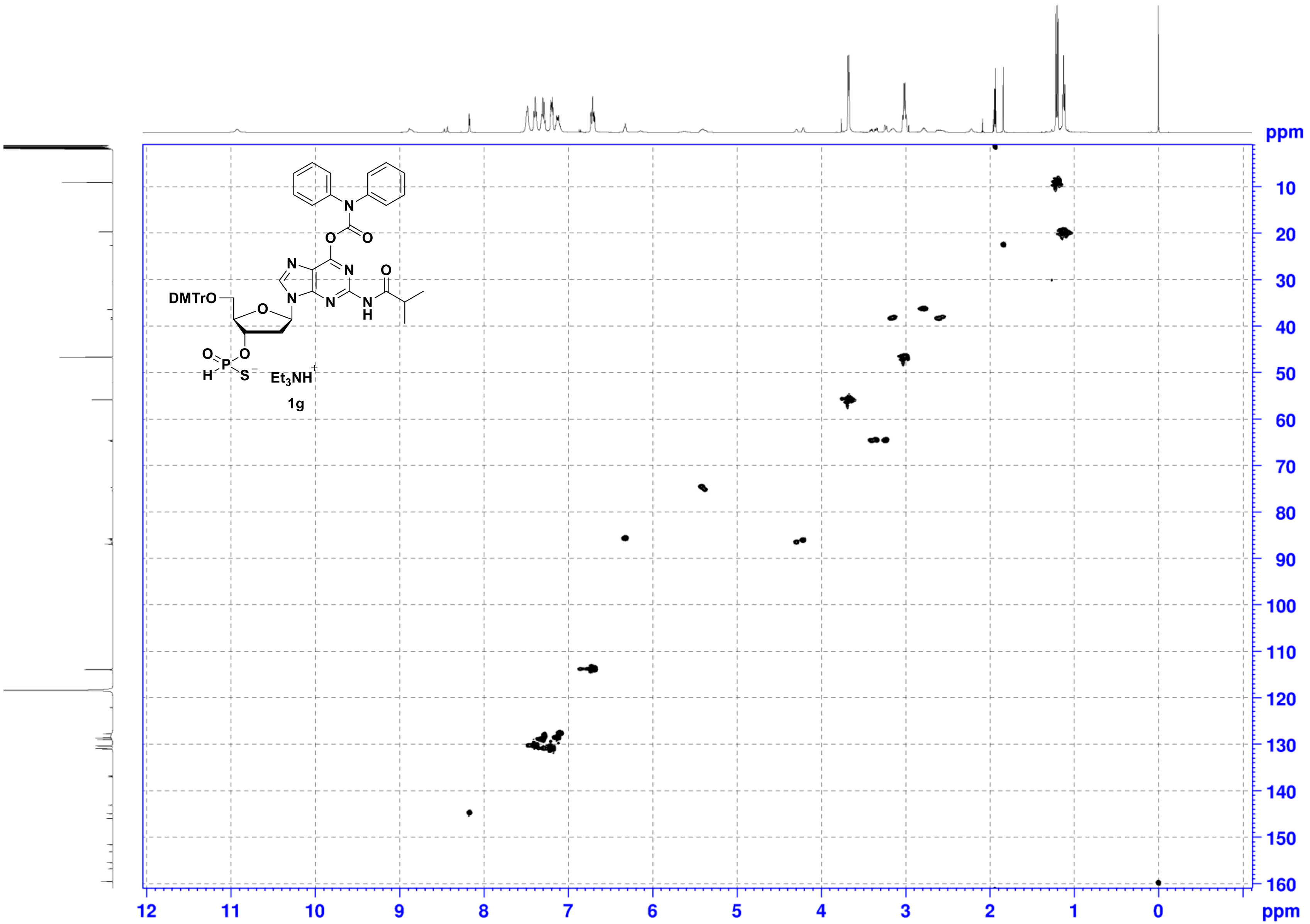

Figure S9 gHSQC spectrum of **1g** (500 MHz, in CD_3CN)

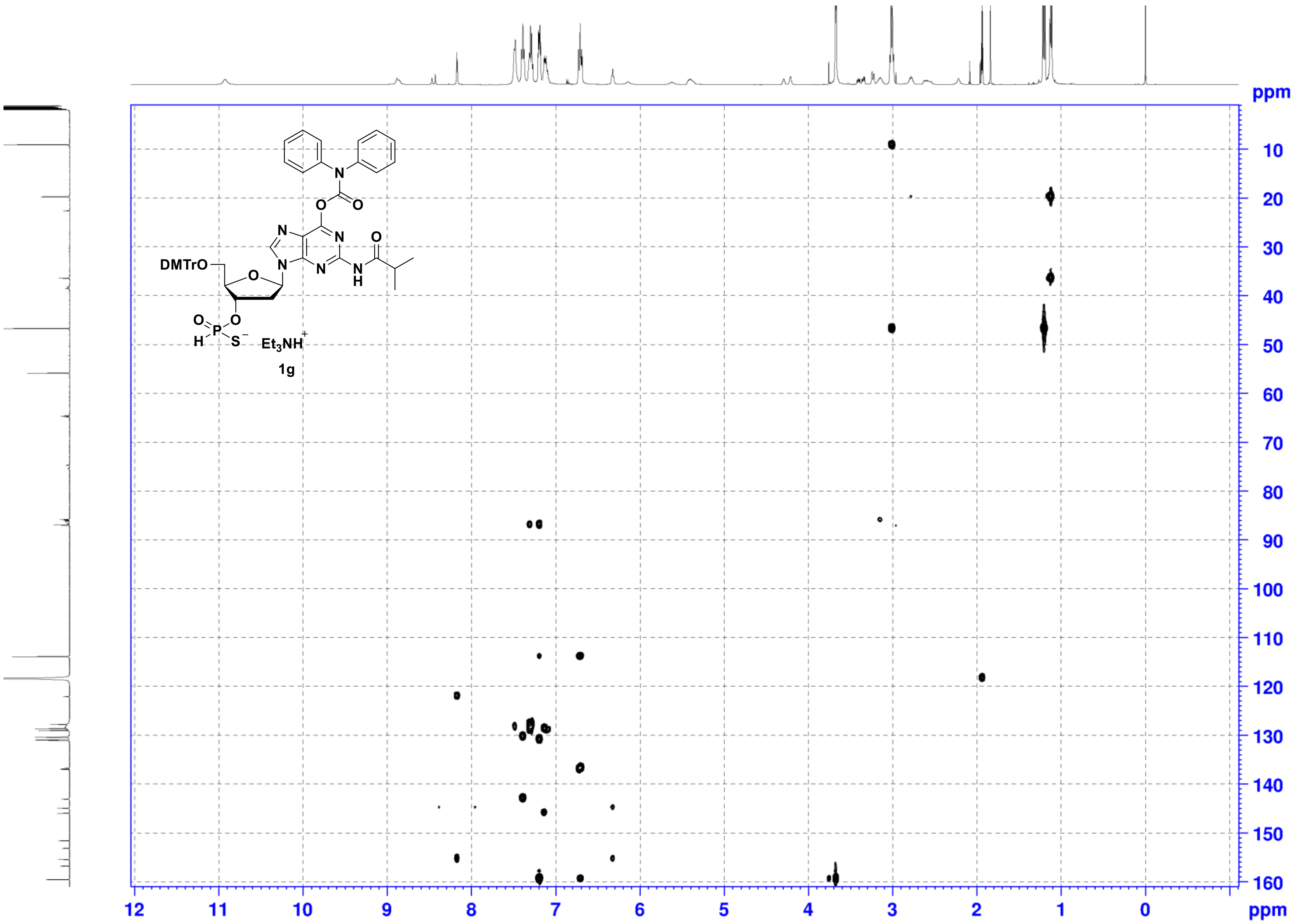

Figure S10 gHMBC spectrum of **1g** (500 MHz, in CD_3CN)

Investigation of condensing reagents for the formation *H*-phosphonate diester from *H*-phosphonothioate monomer

Scheme S2

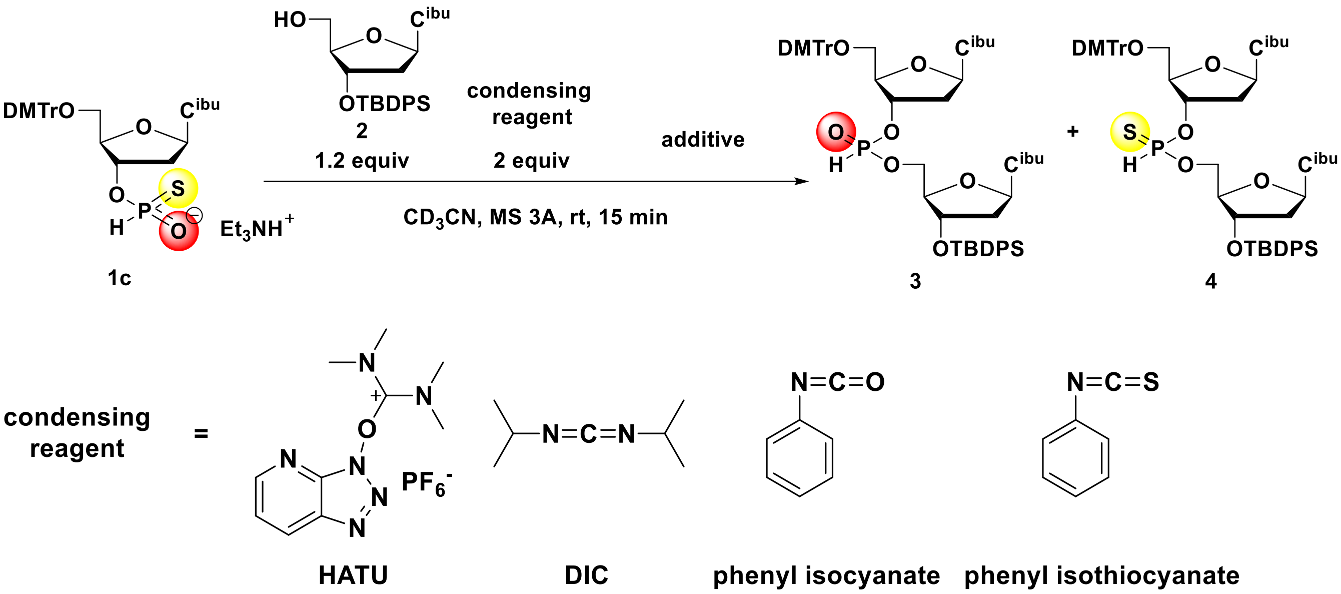

A condensing reagent for *S*-selective activation was investigated as follows: Deoxycytidine 3′-*H*-phosphonothioate monoester **1c**, 1.2 equiv of a nucleoside **2** bearing a free 5′-hydroxy group, and an additive reagent were dissolved in dry CD_3CN, and thoroughly dried over MS 3A, followed by addition of 2 equiv of various condensing reagents (**Scheme S2**). After 15 min, the reaction mixture was analyzed by ^{31}P NMR spectroscopy. The NMR yield was calculated as the integral ratio of the signals corresponding to the desired *H*-phosphonate diester **3** (δ 8–10) and all signals, and the chemoselectivity was estimated on the basis of the integral ratio of the desired *H*-phosphonate diester **3** to the undesired *H*-phosphonothioate diester **4** (δ 71–73).

Table S1

Entry	Condensing reagent	Additive	NMR yield of **3** (%) [a]	Chemoselectivity PO-H:PS-H (**3**:**4**) [b]
1	HATU	2,6-lutidine [c]	3	54:46
2	Phenyl isocyanate	Py·HCl [d]	-	1:>99
3	Phenyl isothiocyanate	Py·HCl [d]	NR	-
4 [e]	DIC	-	-	-
5 [e]	DIC	Py·HCl [d]	76	94:6
6	DIC	Py·HCl [d]	73	84:16

[a] Determined by the ^{31}P NMR integral ratio of d($C_{PO\text{-}H}C$) (**3**).

[b] Determined by the ^{31}P NMR integral ratios of d($C_{PO\text{-}H}C$) (**3**):d($C_{PS\text{-}H}C$) (**4**).

[c] 5 equiv of base were used.

[d] 2 equiv of Py·HCl were used

[e] Pyridine-d_5, MS 4A and 3 equiv of DIC were used as solvent and dehydrating reagent.

Based on HSAB principle, sulfur atoms are softer nucleophile than oxygen atoms. Thus, condensing reagents which are soft electrophiles were chosen as candidates: HATU, used in Entry 1, is considered to be a soft electrophile because the positive charge at the electrophilic center is delocalized by resonance and it has a large LUMO. Other condensing reagents used in Entries 2–6 were also considered to be soft electrophiles because they have cumulated double bonds at the electrophilic centers and a tendency for π electrons to be readily dispersed.

In **Table S1**, Entry 1, HATU and 2,6-lutidine were used as a condensing reagent and a base, and the desired *H*-phosphonate diester **3** was formed only in 3% ^{31}P NMR yield with almost no chemoselectivity (**3**:**4** = 54:46). In addition, ca. 37% of oxidized products were generated as a byproduct. In Entry 2, phenyl isocyanate and Py·HCl were chosen as a condensing reagent and an acidic activator, and signals corresponded to the desired *H*-phosphonate diester was not observed by ^{31}P NMR and undesired *H*-phosphonothioate diester was formed in 26% ^{31}P NMR yield. Thus, using isocyanate derivatives exclusively afforded an *H*-phosphonothioate diester. In Entry 3, we envisioned that the electrophilic center of isothiocyanate is softer than that of isocyanate derivatives and selected phenyl isothiocyanate and Py·HCl as a condensing reagent and an acidic activator. However, these conditions resulted in no reaction. In Entries 4–6, DIC was selected as a condensing reagent. In Entry 4, DIC was used in pyridine-d_5 in the absence of an acidic activator, resulting in no reaction. Next, Py·HCl was used as an acidic activator in Entry 5, and these conditions afforded an internucleotidic *H*-phosphonate diester linkage **3** (δ 8.8, 9.8, $^{1}J_{\text{P-H}}$ = 714 Hz, ca. 76%) in a chemoselective manner (**3**:**4** = 94:6) along with 5% of the *H*-phosphonothioate diester **4** (δ 73.0, 71.9, $^{1}J_{\text{P-H}}$ = 676.8 Hz) and ca. 18% of the 3′-*H*-phosphonate monoester (δ 3.8, $^{1}J_{\text{P-H}}$ = 618.4 Hz). Next, we examined solvent, and CD_3CN was used in place of pyridine-d_5. As a result, the desired *H*-phosphonate diester **3** was formed in 73% NMR yield with moderate chemoselectivity (**3**:**4** = 84:16). Notably, the formation of *H*-phosphonate monoester (δ 3–5) was not detected. According to these results, we concluded that the use of carbodiimide-type condensing reagents in the presence of an acidic activator in CD_3CN was suitable for the formation of the *H*-phosphonate diester from an *H*-phosphonothioate monomer.

The ^{31}P{^{1}H} NMR spectra of these reaction mixture are shown below (**Figures S11–S16**).

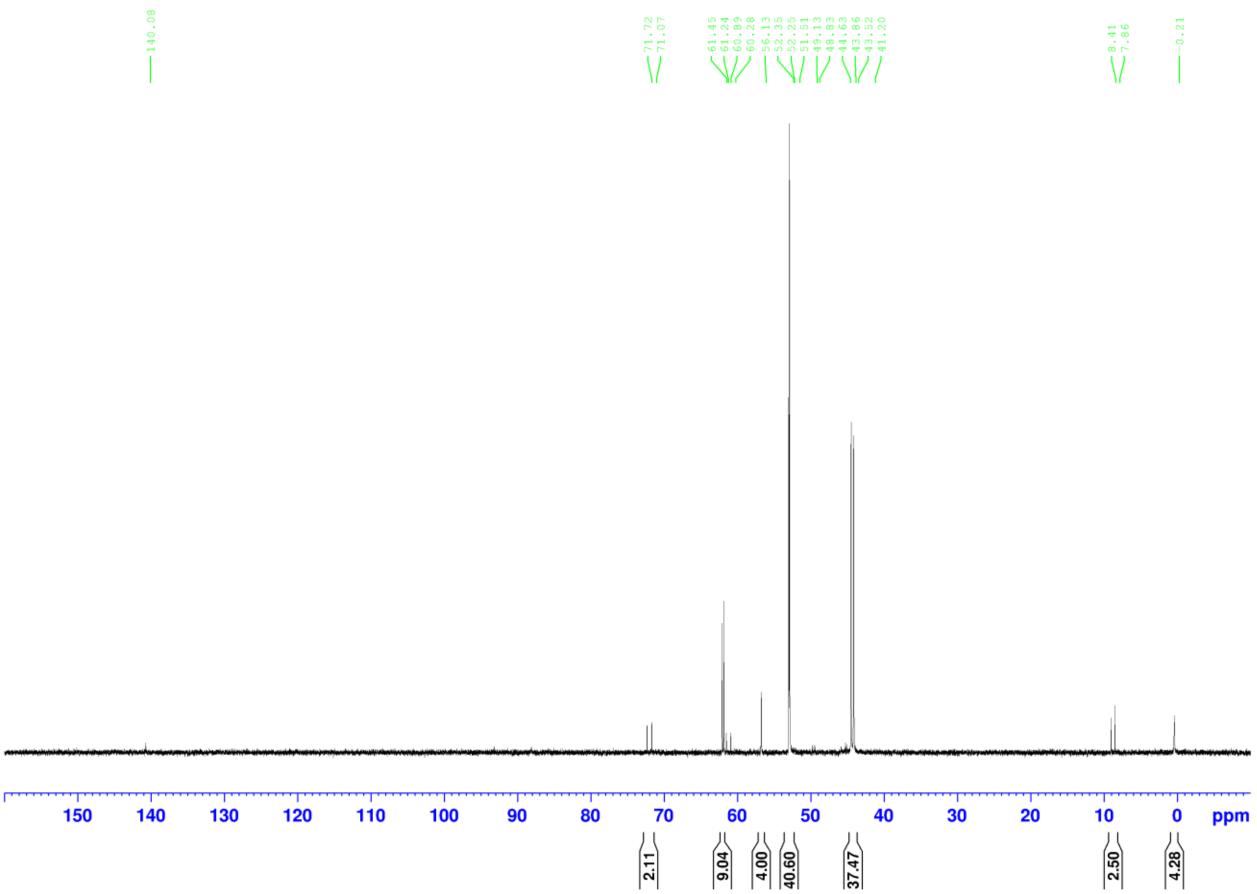

Figure S11 $^{31}P\{^{1}H\}$ NMR spectrum of Entry 1 (202 MHz, in CD_3CN)

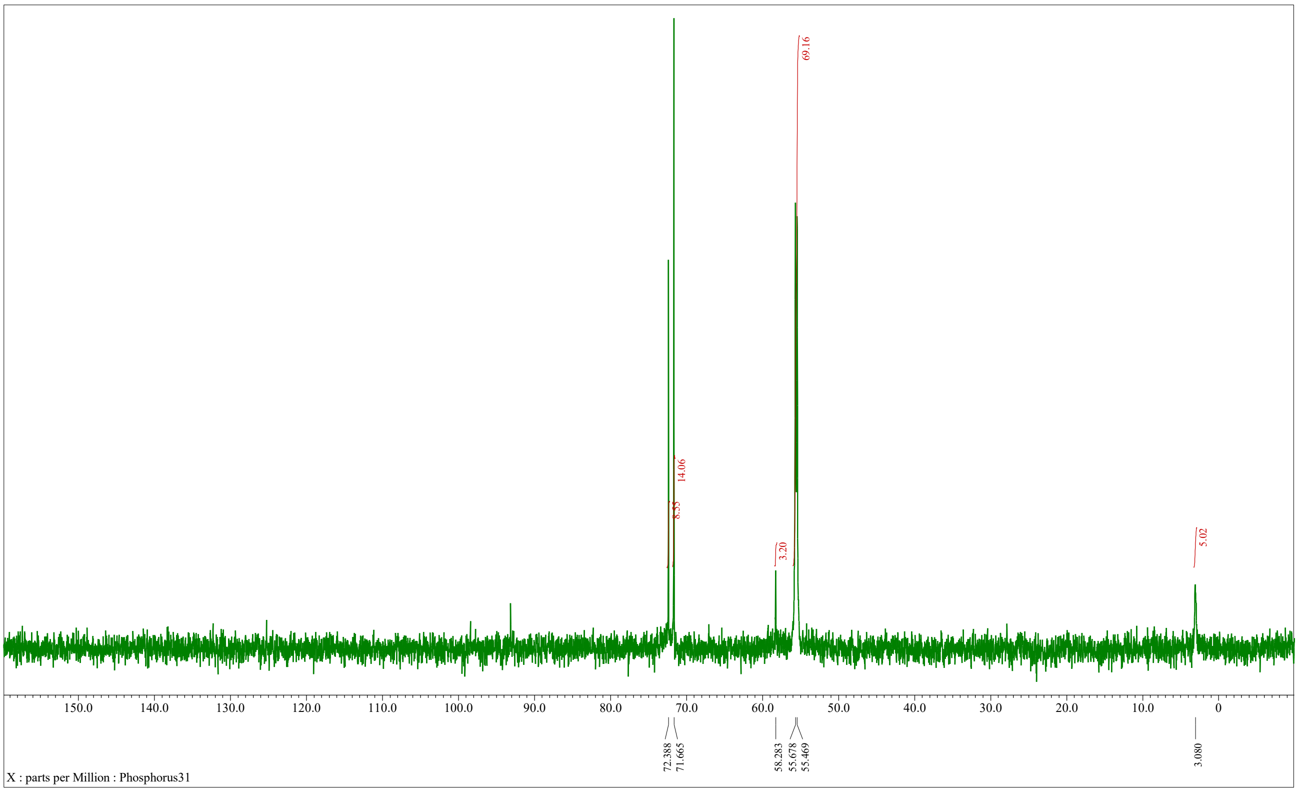

Figure S12 $^{31}P\{^{1}H\}$ NMR spectrum of Entry 2 (161 MHz, in CD_3CN)

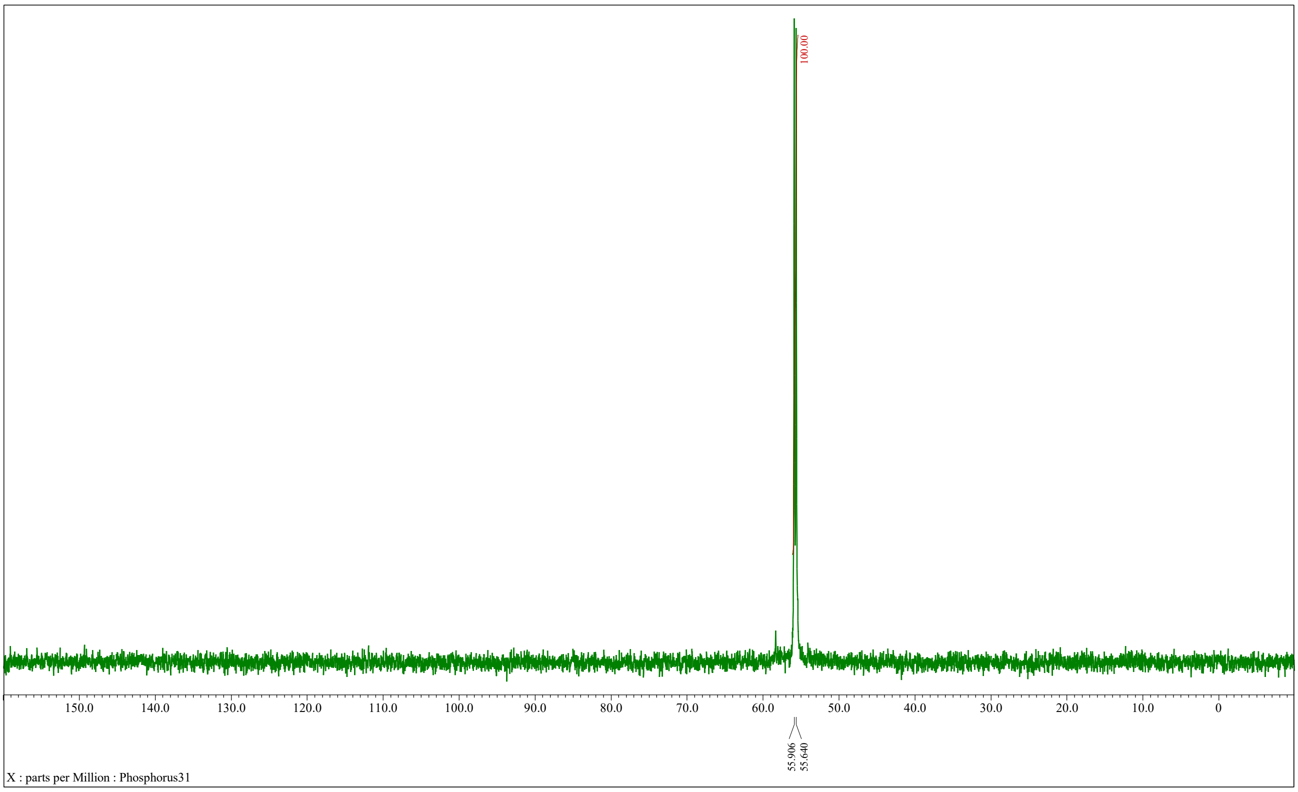

Figure S13 $^{31}P\{^{1}H\}$ NMR spectrum of Entry 3 (161 MHz, in CD_3CN)

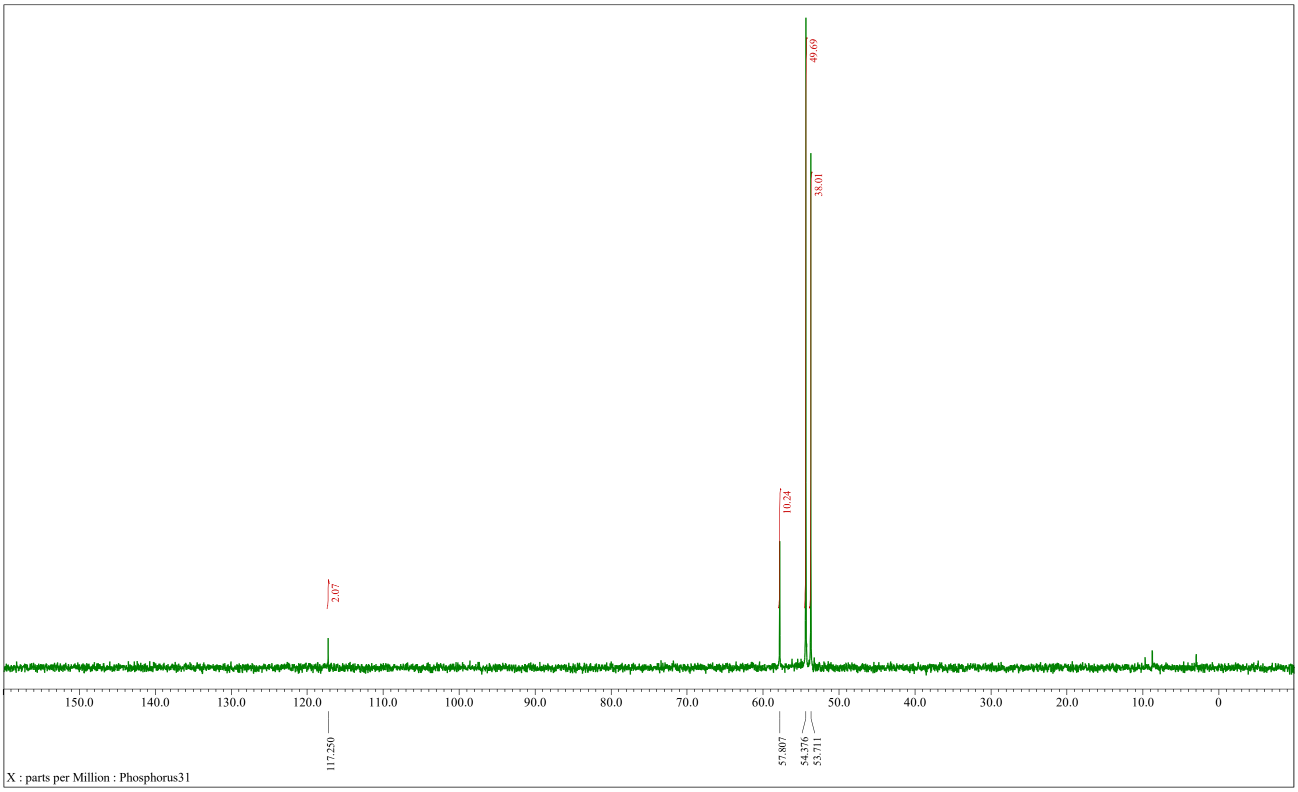

Figure S14 $^{31}P\{^{1}H\}$ NMR spectrum of Entry 4 (161 MHz, in pyridine-d_5)

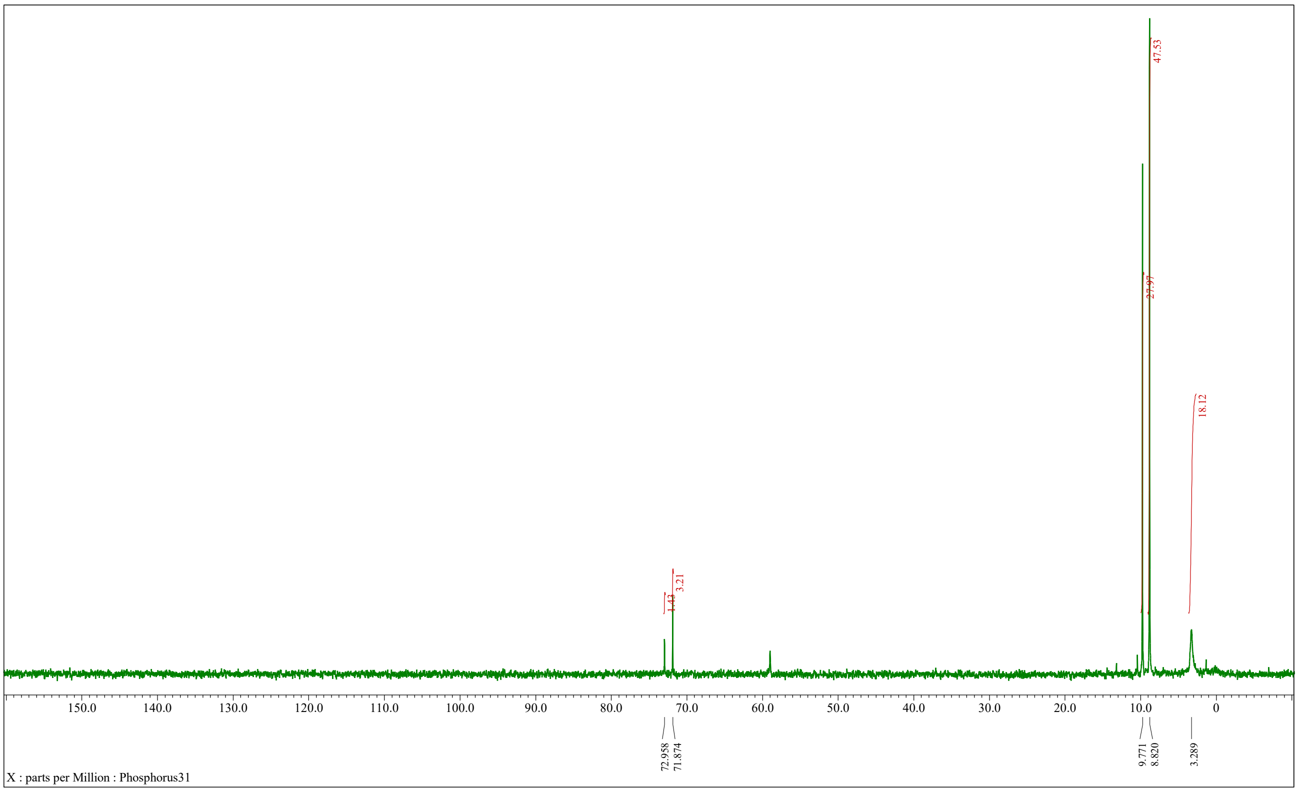

Figure S15 $^{31}P\{^1H\}$ NMR spectrum of Entry 5 (161 MHz, in pyridine-d_5)

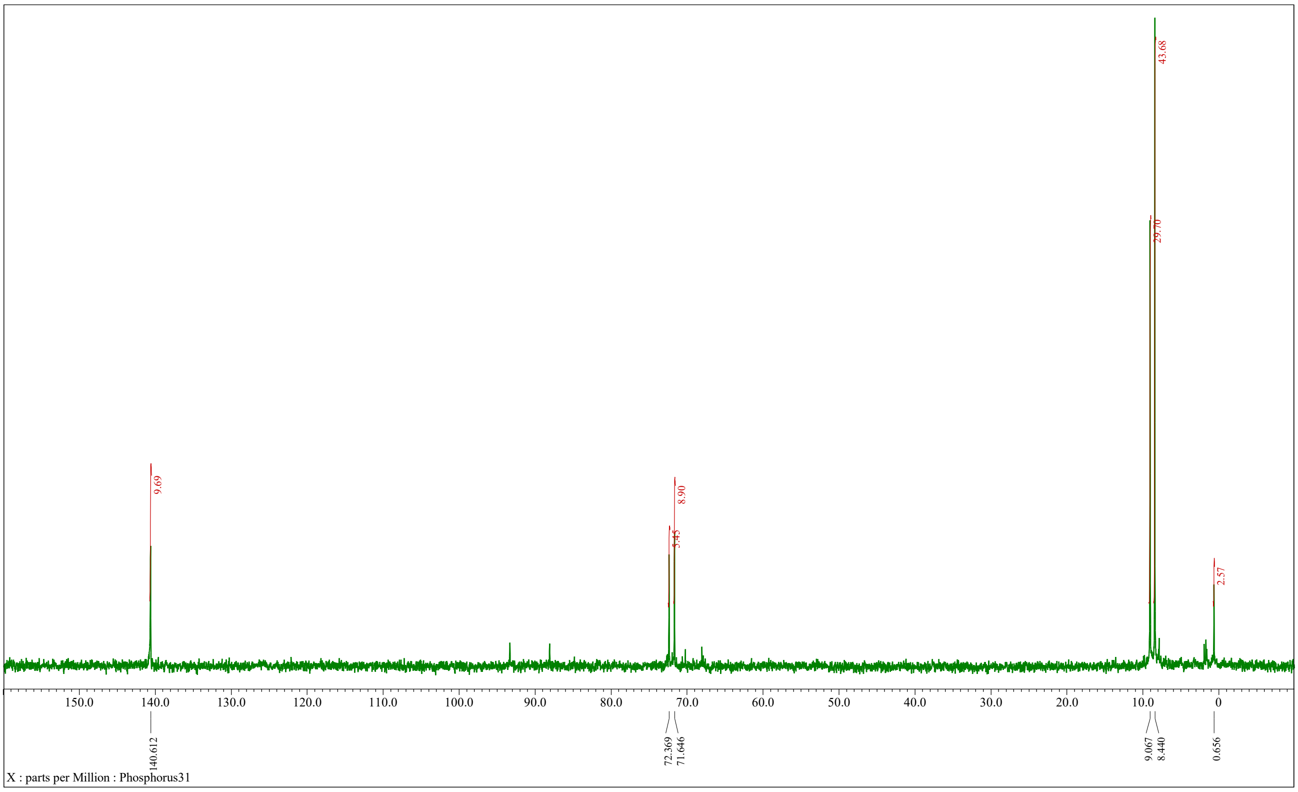

Figure S16 $^{31}P\{^1H\}$ NMR spectrum of Entry 6 (161 MHz, in CD_3CN)

Screening of carbodiimide derivatives

Scheme S3

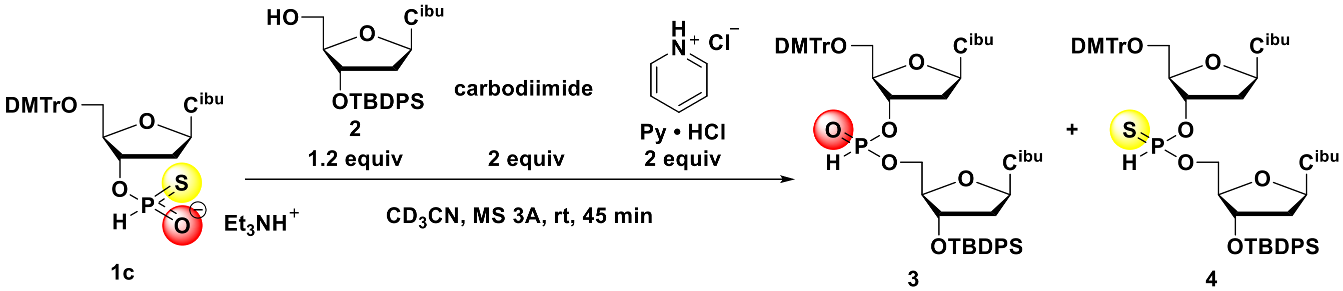

Table S2

Entry	Carbodiimide	NMR yield of **3** (%) [a]	Chemoselectivity PO-H:PS-H (**3**:**4**) [b]
1 [c]	*N,N′*-diisopropyl (DIC)	73	84:16
2	*N,N′*-di-*tert*-butyl (DTBC)	-	1:>99
3	*N,N′*-dicyclohexyl (DCC)	62	77:23
4	1-(3-dimethylaminopropyl) -3-ethyl hydrochloride (EDC·HCl)	70	85:15

[a] Determined by the ^{31}P NMR integral ratio of d($C_{PO-H}C$) (**3**).

[b] Determined by the ^{31}P NMR integral ratios of d($C_{PO-H}C$) (**3**):d($C_{PS-H}C$) (**4**).

[c] The ^{31}P NMR analysis was conducted after 15 min from initiate reaction.

The optimal carbodiimide derivative was investigated as follows: Deoxycytidine 3′-*H*-phosphonothioate monoester derivative **1c**, 1.2 equiv of a nucleoside **2** bearing a free 5′-hydroxy group, and Py·HCl were dissolved in dry CD_3CN, and thoroughly dried over MS 3A followed by addition of 2 equiv of carbodiimide derivatives (**Table S2**). After 15 min (in Entry 1) or 45 min (in Entries 2–4), the reaction was analyzed by ^{31}P NMR spectroscopy. The NMR yield was calculated as the integral ratio of the signals corresponding to the desired *H*-phosphonate diester **3** (δ 8–10) and all signals, and the chemoselectivity was estimated on the basis of the integral ratio of the desired *H*-phosphonate diester **3** to the undesired *H*-phosphonothioate diester **4** (δ 71–73).

In Entry 2, *N,N′*-di-*tert*-butylcarbodiimide (DTBC) was used and the desired *H*-phosphonate diester **3** was not obtained along with 5% of the undesired *H*-phosphonothioate diester **4** (**3**:**4** = 1:>99) and ca. 93% of the *H*-phosphonothioate monomer **1c**. *N,N′*-Dicyclohexylcarbodiimide (DCC) gave a similar result to DIC, and the desired *H*-phosphonate diester **3** was formed with 62% NMR yield with moderate chemoselectivity (Entry 3, **3**:**4** = 77:23). Using 1-(3-dimethylaminopropyl)-3-ethylcarbodiimide hydrochloride (EDC·HCl) afforded the desired *H*-phosphonate diester **3** with 70%NMR yield and moderate chemoselectivity (Entry 4, **3**:**4** = 85:15). However, the minor signals except for the desired *H*-phosphonate diester **3** were observed at δ 7–10 in the ^{31}P NMR spectrum. We attributed it to the elimination of DMTr group in the presence of Py·HCl and

EDC·HCl.

The $^{31}P\{^{1}H\}$ NMR spectra of these reaction mixture are shown below (**Figures S16–S19**).

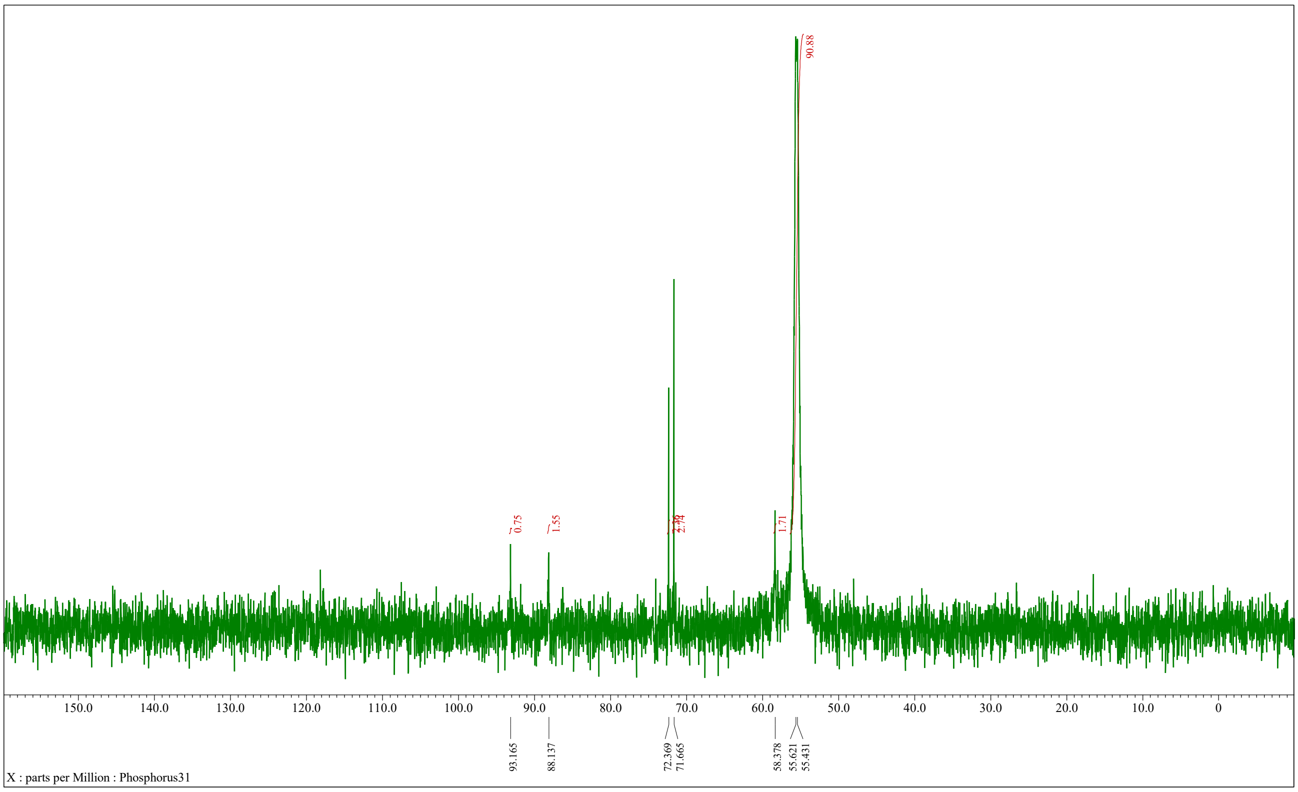

Figure S17 $^{31}P\{^{1}H\}$ NMR spectrum of Entry 2 (161 MHz, in CD_3CN)

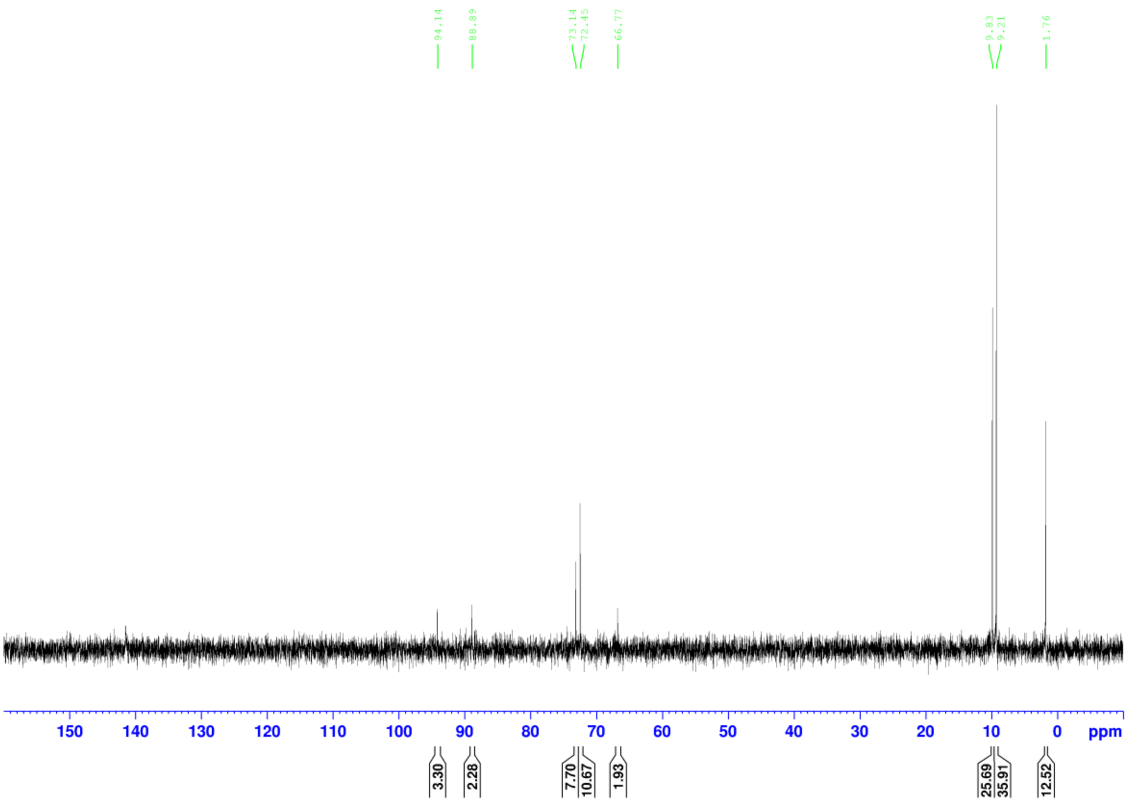

Figure S18 $^{31}P\{^1H\}$ NMR spectrum of Entry 3 (243 MHz, in CD_3CN)

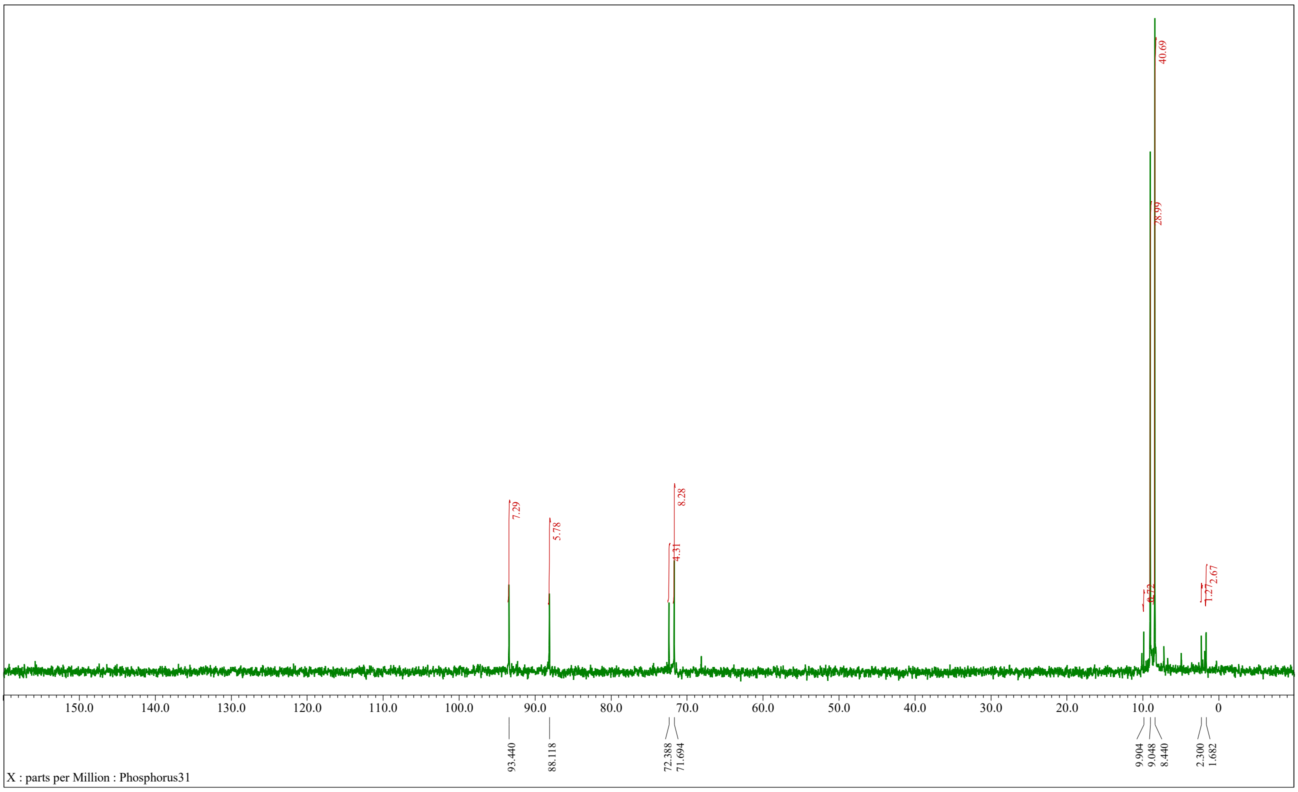

Figure S19 $^{31}P\{^1H\}$ NMR spectrum of Entry 4 (161 MHz, in CD_3CN)

$^{31}P\{^1H\}$ NMR spectra of Scheme 2

Scheme 2

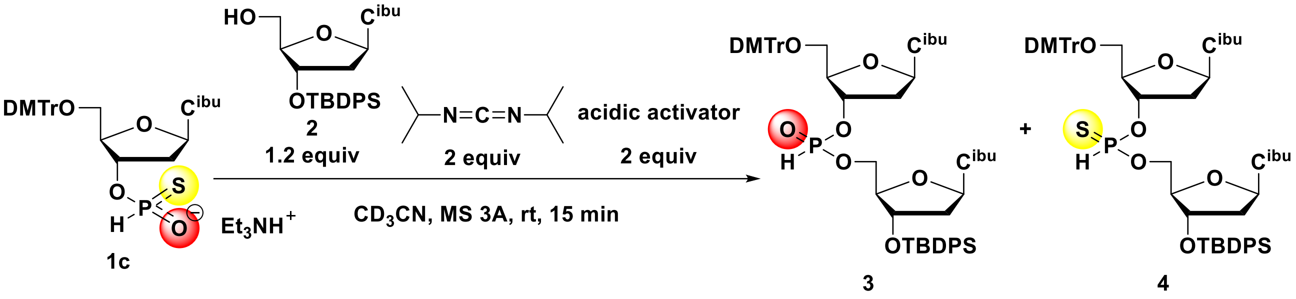

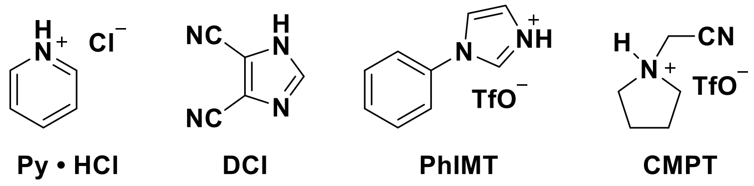

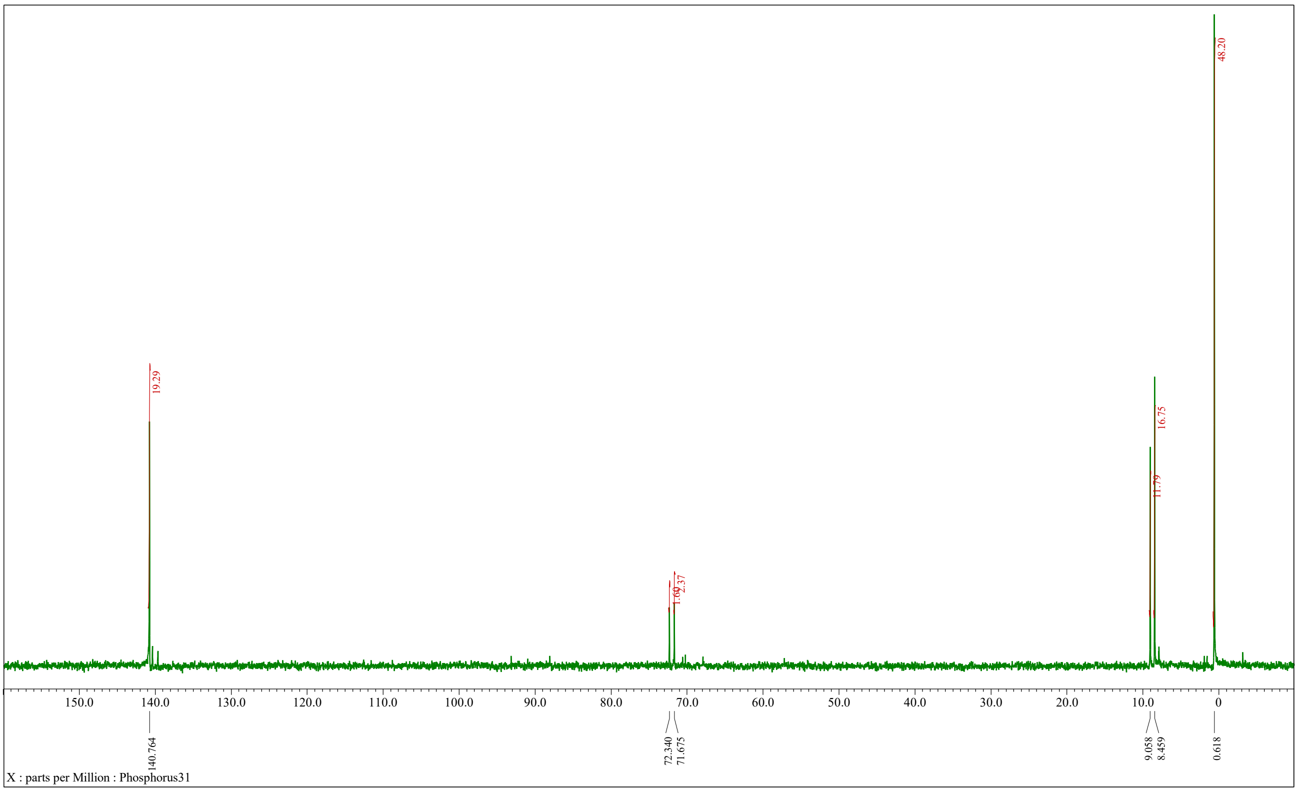

Figure S20 $^{31}P\{^1H\}$ NMR spectrum of Entry 2 (161 MHz, in CD_3CN)

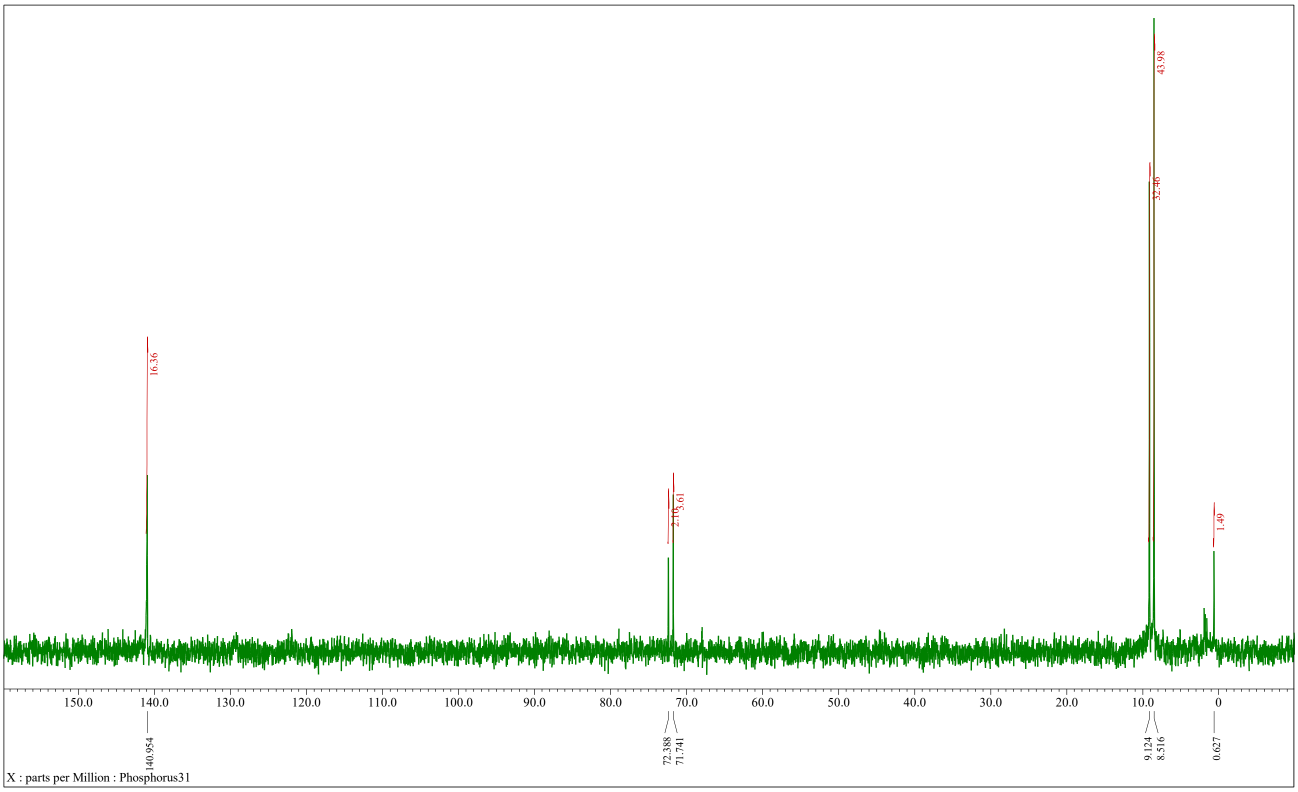

Figure S21 $^{31}P\{^{1}H\}$ NMR spectrum of Entry 3 (161 MHz, in CD_3CN)

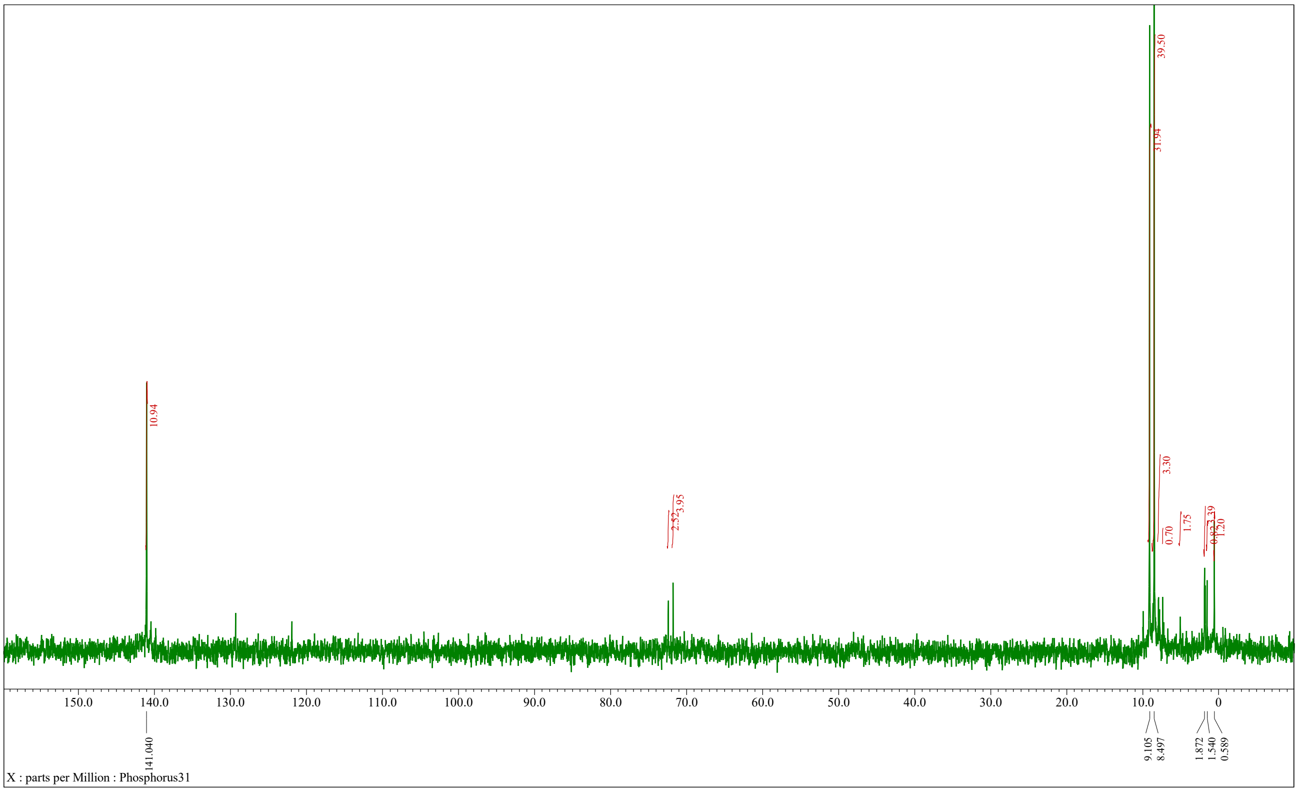

Figure S22 $^{31}P\{^{1}H\}$ NMR spectrum of Entry 4 (161 MHz, in CD_3CN)

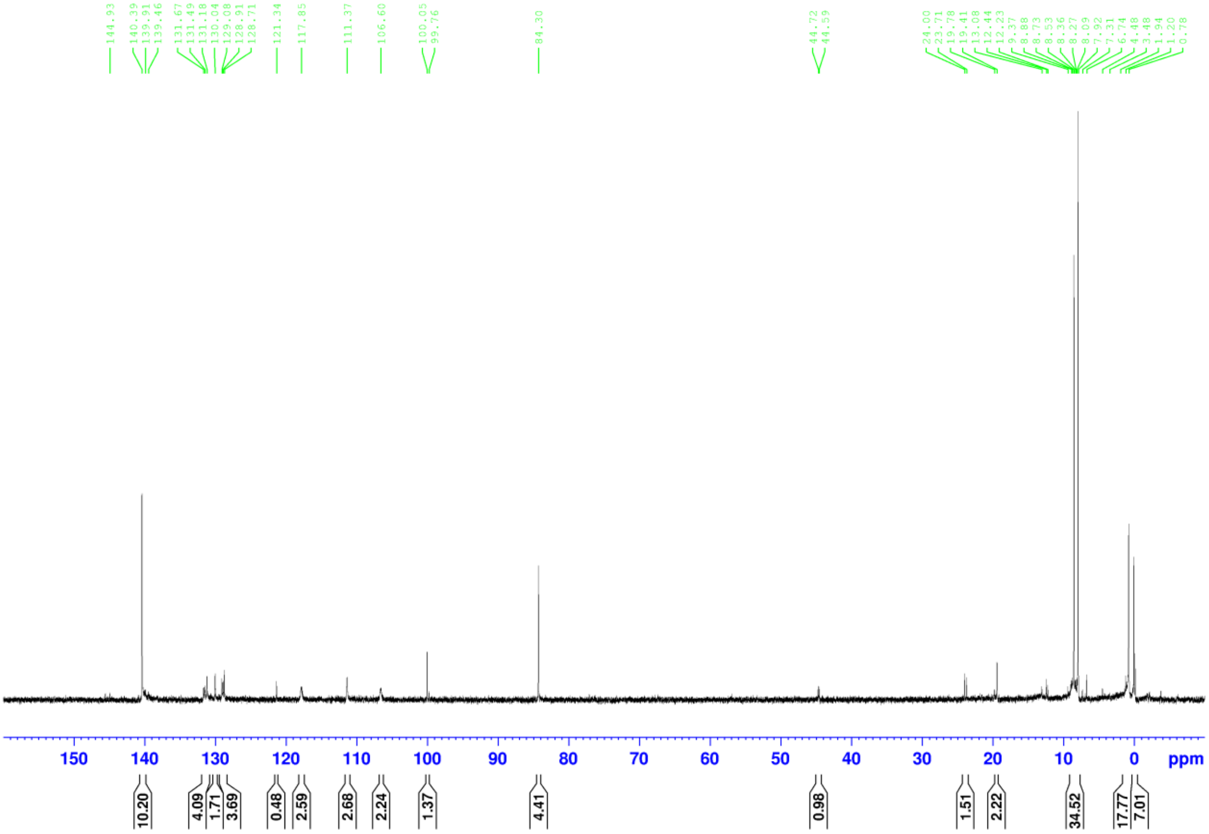

Figure S23 $^{31}P\{^{1}H\}$ NMR spectrum of Entry 5 (202 MHz, in CD_3CN)

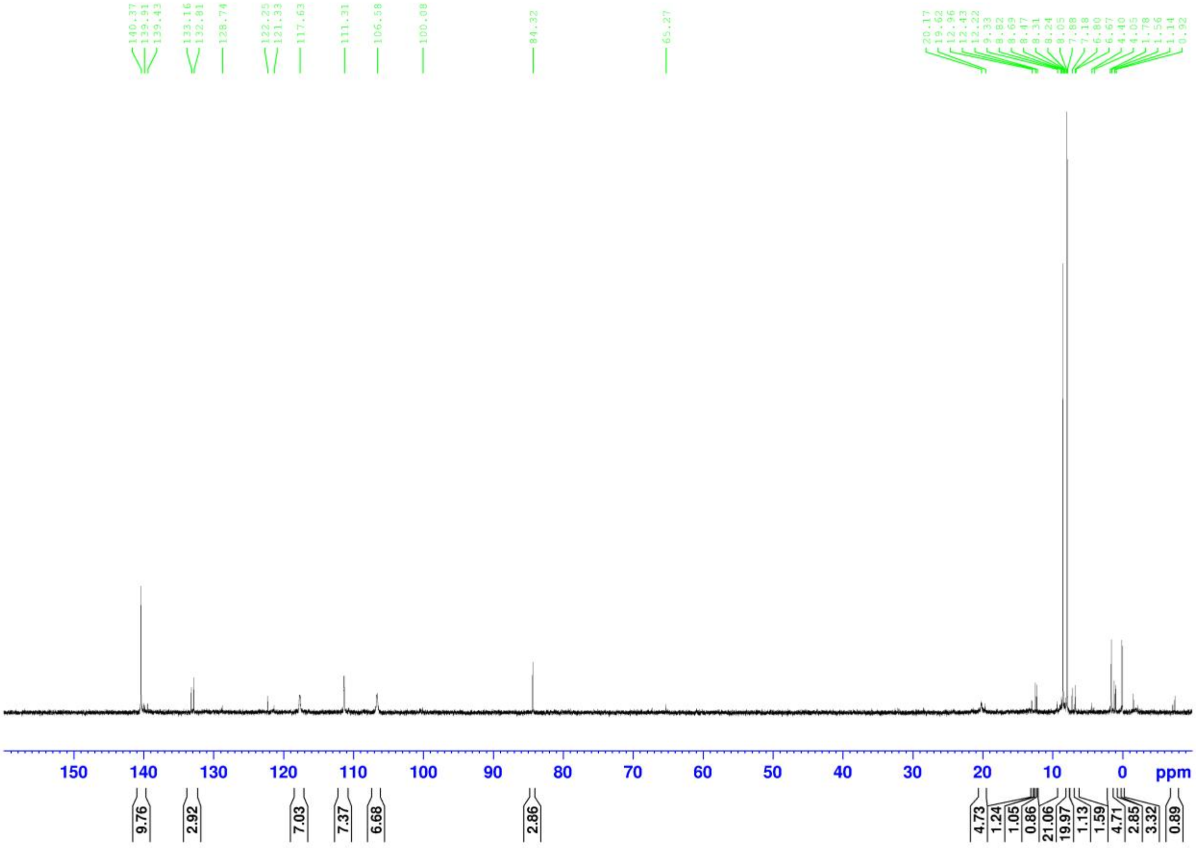

Figure S24 $^{31}P\{^{1}H\}$ NMR spectrum of Entry 6 (202 MHz, in CD_3CN)

$^{31}P\{^{1}H\}$ NMR spectra of Scheme 3

Scheme 3

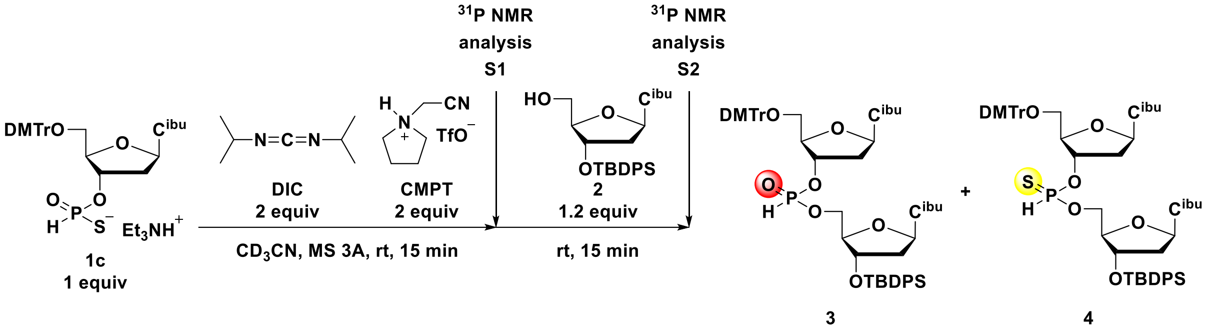

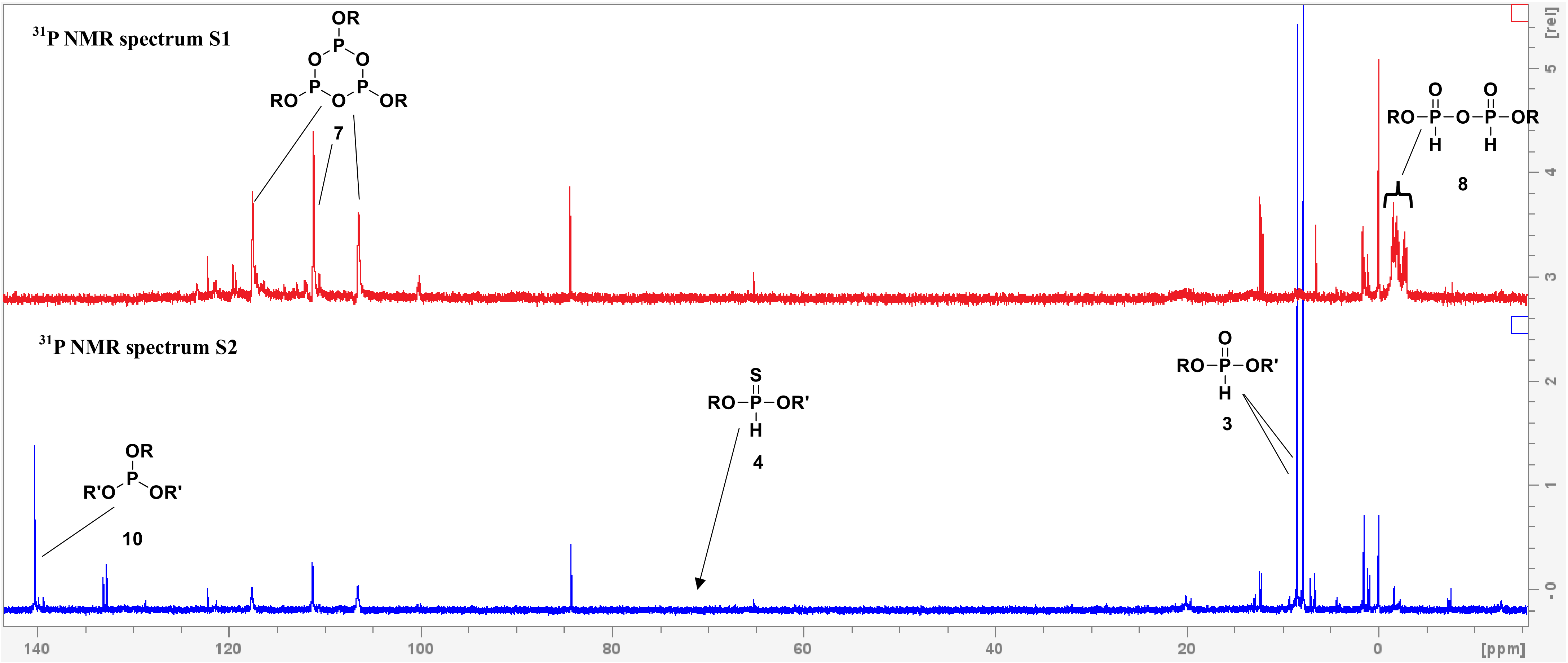

Figure S25 $^{31}P\{^{1}H\}$ NMR spectra of **Scheme S3** (202 MHz, in CD_3CN)

Evaluation of *S*-selectivity by using *N,N′*-diisopropylcarbodiimide (DIC)

To elucidate the *S*-selectivity for the formation of the *H*-phosphonate diester **3**, we conducted a reaction monitoring using ^{13}C NMR spectroscopy (**Scheme S4**). First, *H*-phosphonothioate monomer **1c**, compound **2** bearing 5′-hydroxy group, and CMPT were dissolved in dry CD_3CN and dried over MS 3A, followed by the addition of 2 equiv of DIC. After 15 min, the reaction mixture was analyzed by ^{13}C NMR to observe the characteristic signals of DIC (δ 141.1), *N,N′*-diisopropylurea (δ 158.0), and *N,N′*-diisopropylthiourea (δ 181.4). DIC and *N,N′*-diisopropylthiourea were purchased from Tokyo Chemical Industry and *N,N′*-diisopropylurea was purchased from Chem Scene.

Scheme S4

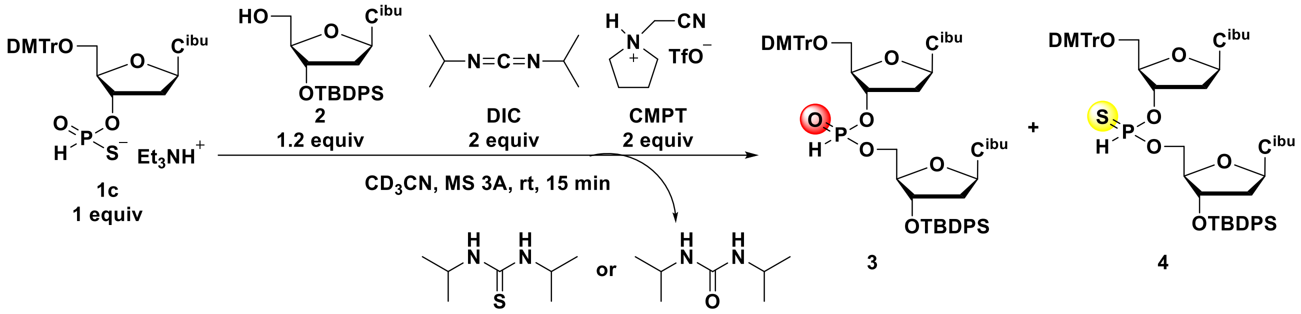

As a result, the signal of the thiocarbonyl group of *N,N′*-diisopropylthiourea was observed at δ 181.4 (**Figures S26–S29**). Therefore, the result indicated that the use of carbodiimide-type condensing reagent resulted in a selective activation of the sulfur atom of an *H*-phosphonothioate monoester.

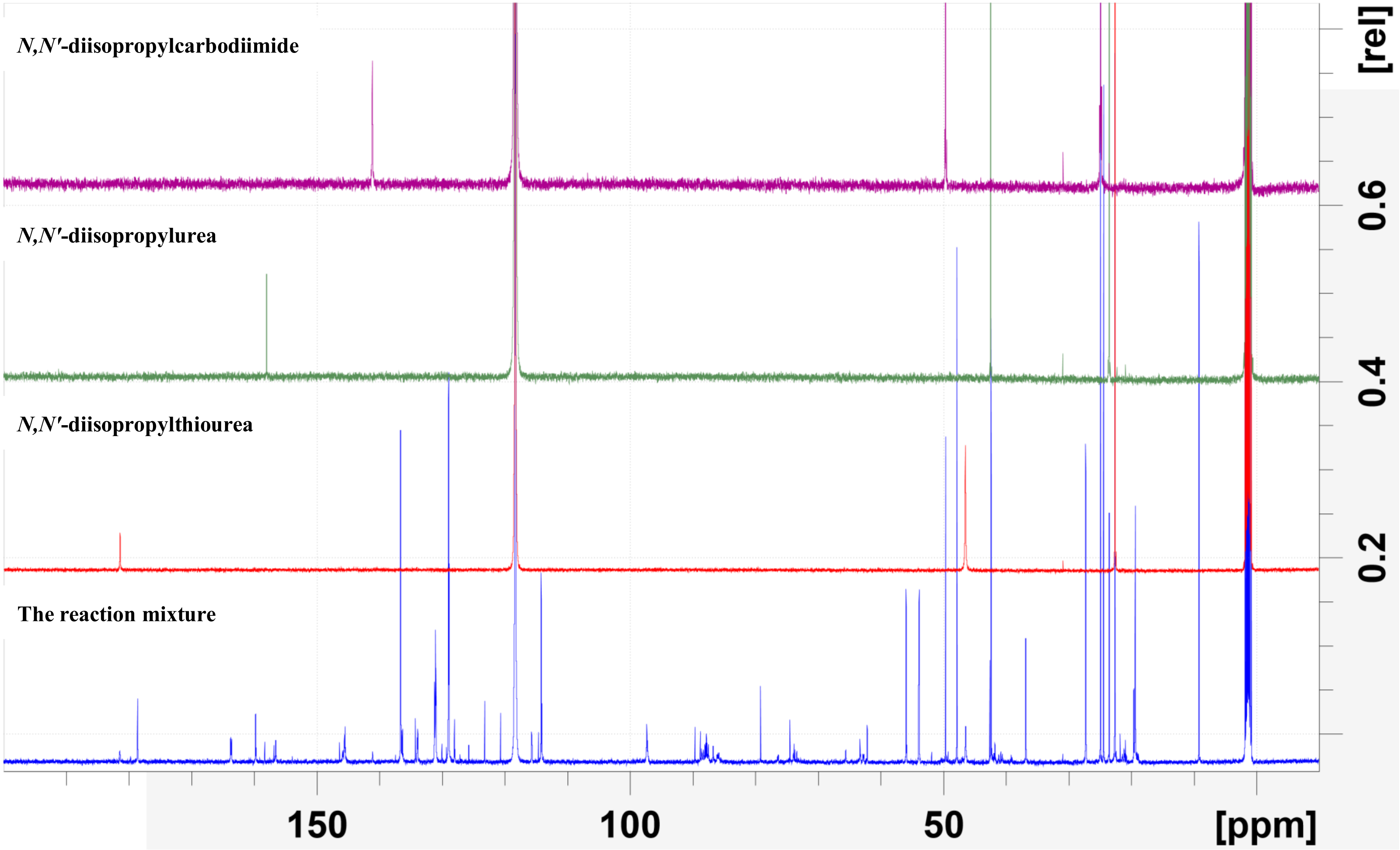

Figure S26 $^{13}C\{^1H\}$ NMR spectra of **Scheme S4**

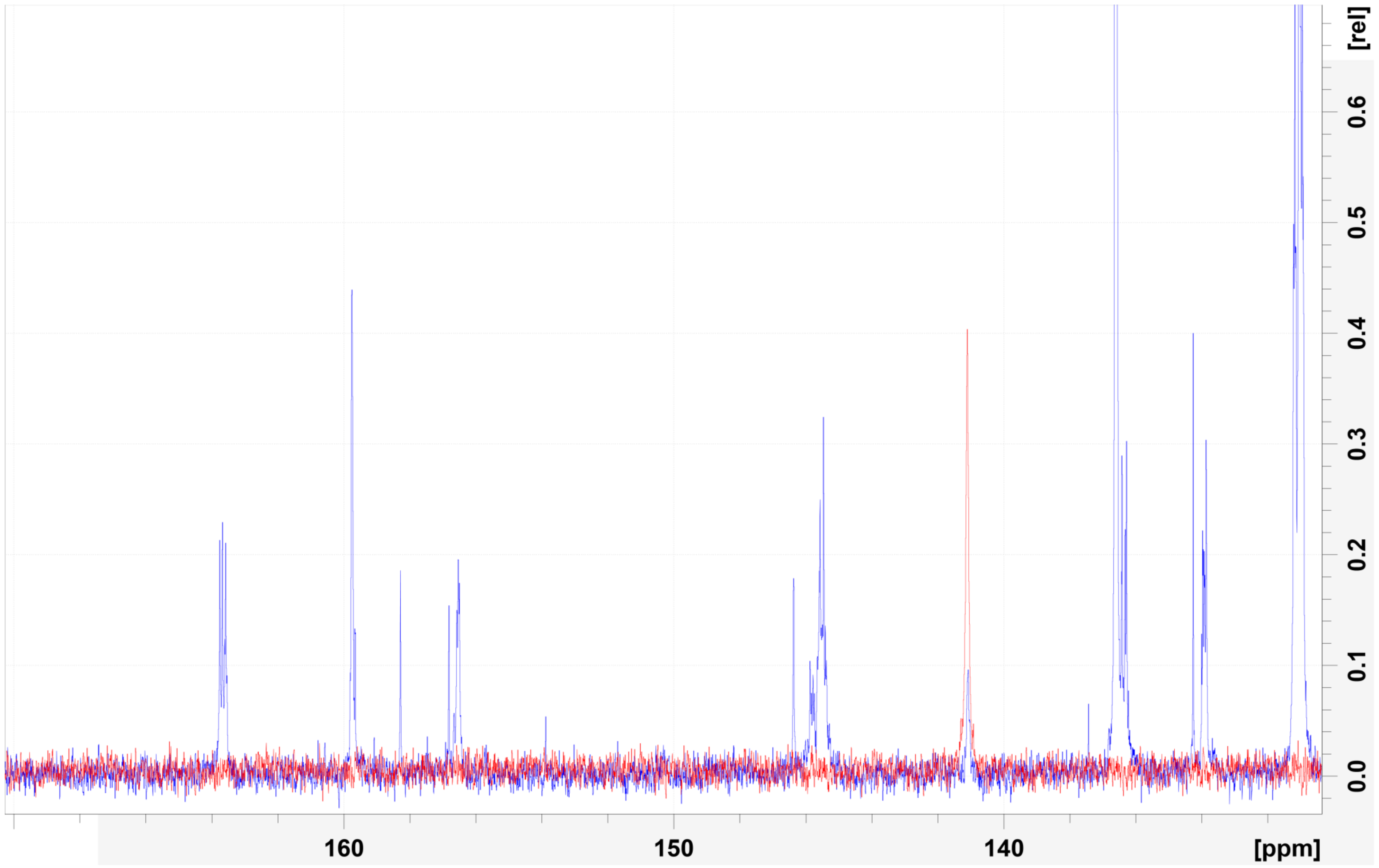

Figure S27 $^{13}C\{^{1}H\}$ NMR spectra of the reaction mixture (blue) and DIC (red)

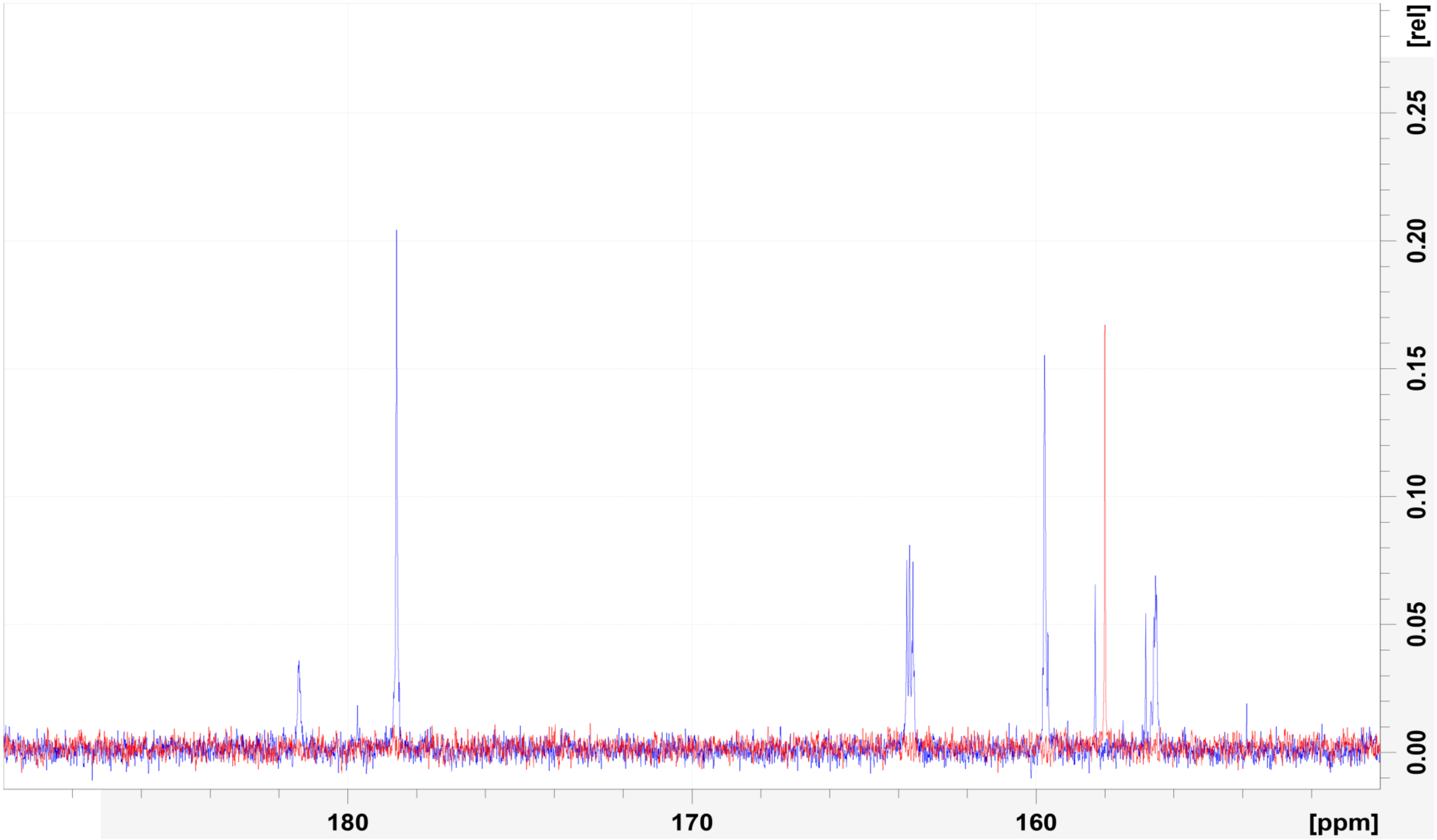

Figure S28 $^{13}C\{^{1}H\}$ NMR spectra of the reaction mixture (blue) and *N,N'*-diisopropylurea (red)

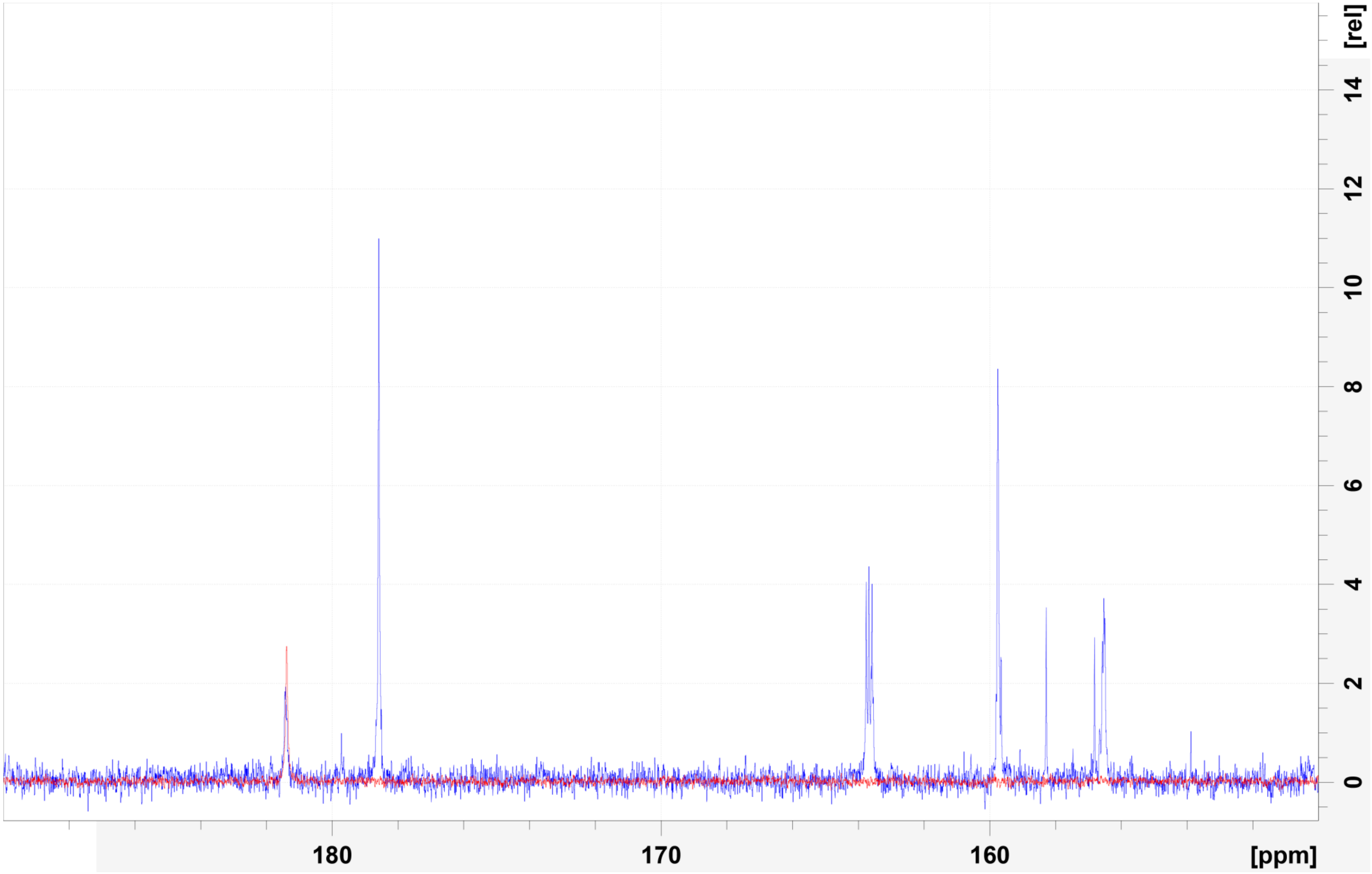

Figure S29 $^{13}C\{^1H\}$ NMR spectra of the reaction mixture (blue) and *N,N'*-diisopropylthiourea (red)

Plausible mechanism for the formation of an *H*-phosphonate diester

In liquid-phase synthesis without the pre-activation protocol (**Scheme S5**), according to the results of **Scheme S4**, this chemoselectivity might be almost complete, and *S*-amidyl intermediate **S2** was generated by nucleophilic attack of a sulfur atom of *H*-phosphonothioate monoester **1** to the carbon atom of DIC. Then, a nucleophilic attack of 5′-hydroxy group to the tautomer **5** would give the desired *H*-phosphonate diester **3**. In addition, the reaction of tautomer **5** and the oxygen atom of the *H*-phosphonothioate monoester would afford unsymmetrical pyrophosphonothioate **6**. The participation of unsymmetrical pyrophosphonothioate **6** as one of the reaction intermediates cannot be excluded. Owing to the presence of two types of phosphorus atoms in pyrophosphonothioate **6**, one bearing a P=O bond and the other a P=S bond, the reaction of this intermediate with a 5′-hydroxy group would afford *H*-phosphonate and *H*-phosphonothioate diesters, deteriorating the chemoselectivity. On the other hand, the reaction of the tautomer **5** and the sulfur atom of *H*-phosphonothioate monoester **1** should afford symmetrical pyrophosphonate **S3**. Since the symmetrical pyrophosphonate **S3** bearing only P=O bond only formed *H*-phosphonate diester by reaction with 5′-hydroxy group, the deteriorating the chemoselectivity might not be cause.

Scheme S5

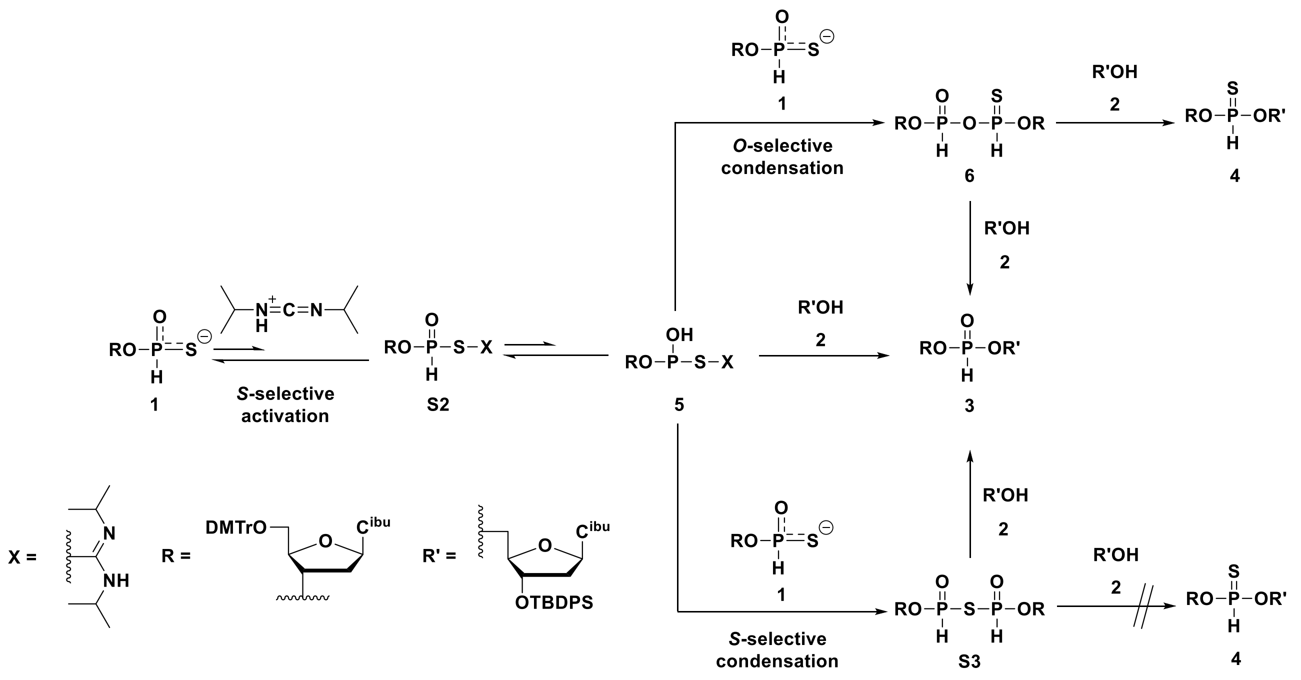

However, in solid-phase synthesis or the pre-activation protocol, the pyrophosphonothioate **6** was re-activated by DIC and formed compound **S4** by nucleophilic attack of an oxygen atom of *H*-phosphonothioate monoester. Subsequently, trimetaphosphite **7** was formed via the re-activation of the sulfur atom by DIC and an intramolecular cyclization (**Scheme S6**). In addition, compounds **8**, **9**, and **S5** might be formed due to hydrolysis or attack of nucleophiles. The reaction of desulfurized derivatives **7**, **8**, or **9** with a 5′-hydroxy group would afford the formation of *H*-phosphonate diester linkages. Additionally, in solid-phase synthesis, due to the immobilization of nucleoside bearing a 5′-hydroxy group on the solid support, the increased distance between nucleosides compared to liquid-phase synthesis is anticipated to suppress the formation of

phosphite triester **10**.

Scheme S6

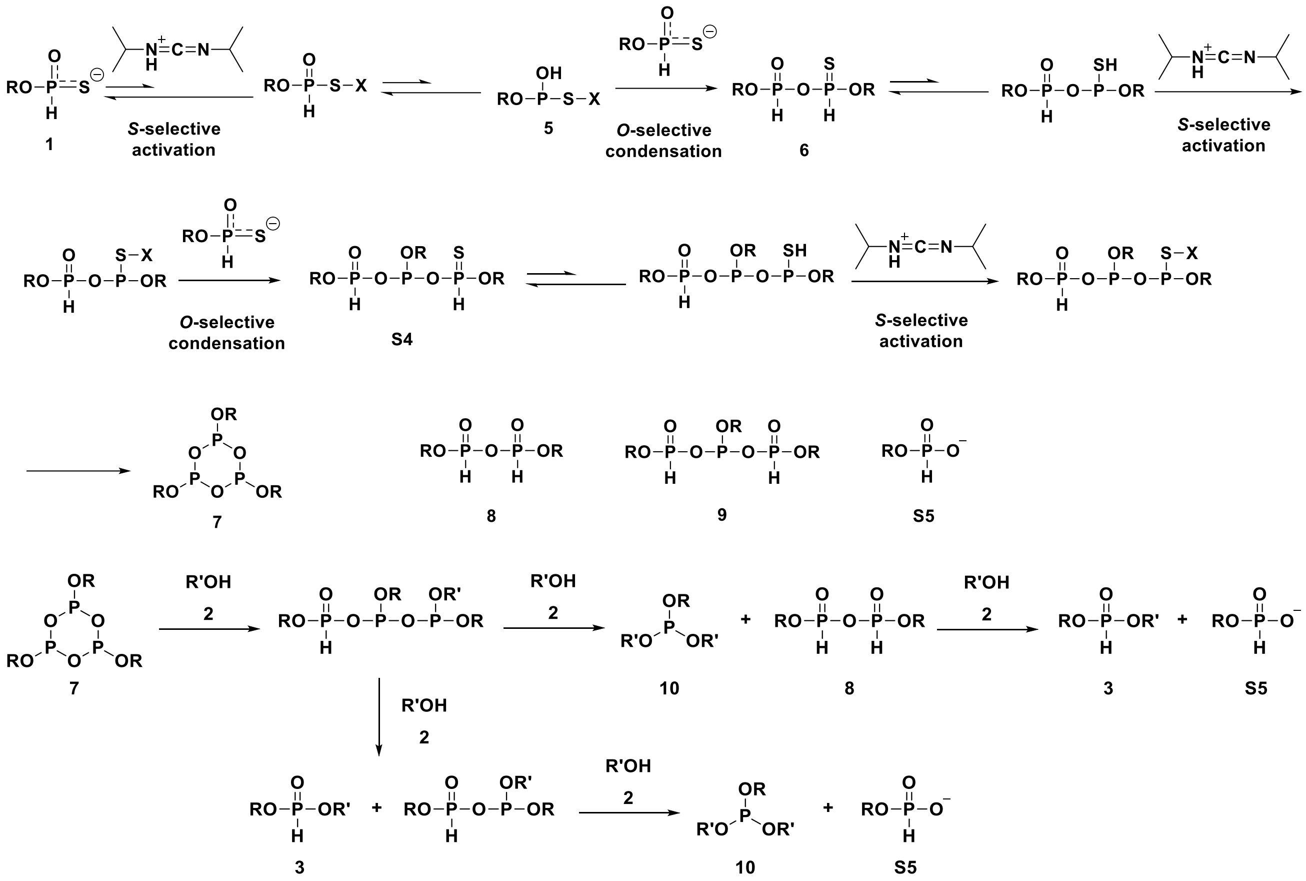

RP-HPLC profiles of N$_{PS}$T dimer

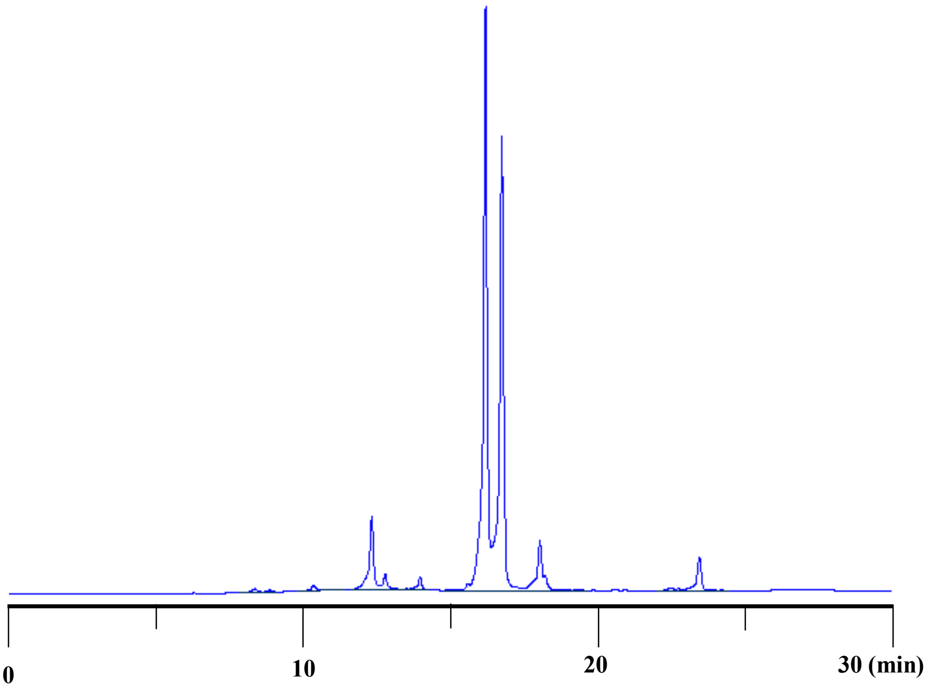

Figure S30 RP-HPLC profiles of dC$_{PS}$T dimer in **Table 2**, Entry 1

RP-HPLC (C18 (5 µm, 100 Å, 3.9 × 150 mm), 0.1 M triethylammonium acetate buffer (pH 7.0)/MeCN = 100/0–70/30 over 30 min, flow rate = 0.5 mL/min, l = 260 nm, t = 30°C) tR = 16.3, 16.9 min (diastereomers)

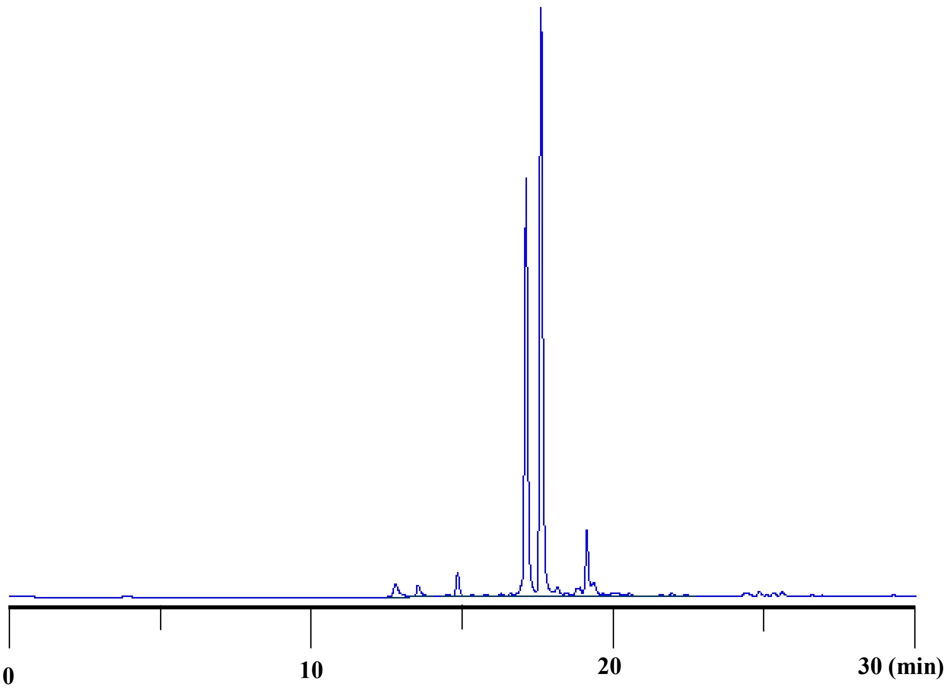

Figure S31 RP-HPLC profiles of dC$_{PS}$T dimer in **Table 2**, Entry 2

RP-HPLC (C18 (5 µm, 100 Å, 3.9 × 150 mm), 0.1 M triethylammonium acetate buffer (pH 7.0)/MeCN = 100/0–70/30 over 30 min, flow rate = 0.5 mL/min, l = 260 nm, t = 30°C) tR = 17.2, 17.6 min (diastereomers)

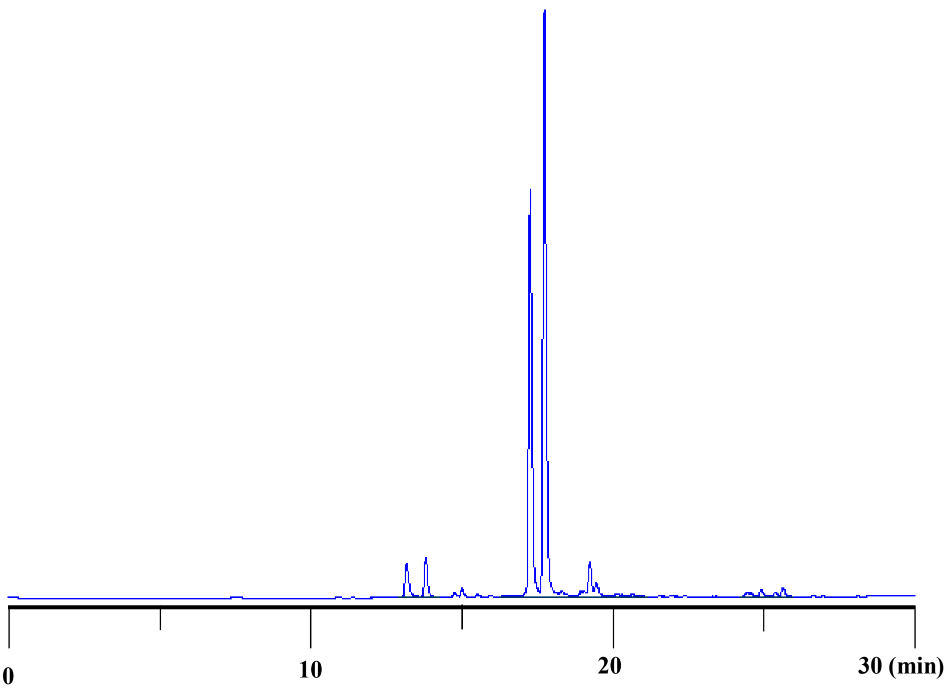

Figure S32 RP-HPLC profiles of dC$_{PS}$T dimer in **Table 2**, Entry 3

RP-HPLC (C18 (5 µm, 100 Å, 3.9 × 150 mm), 0.1 M triethylammonium acetate buffer (pH 7.0)/MeCN = 100/0–70/30 over 30 min, flow rate = 0.5 mL/min, l = 260 nm, t = 30°C) tR = 17.3, 17.8 min (diastereomers)

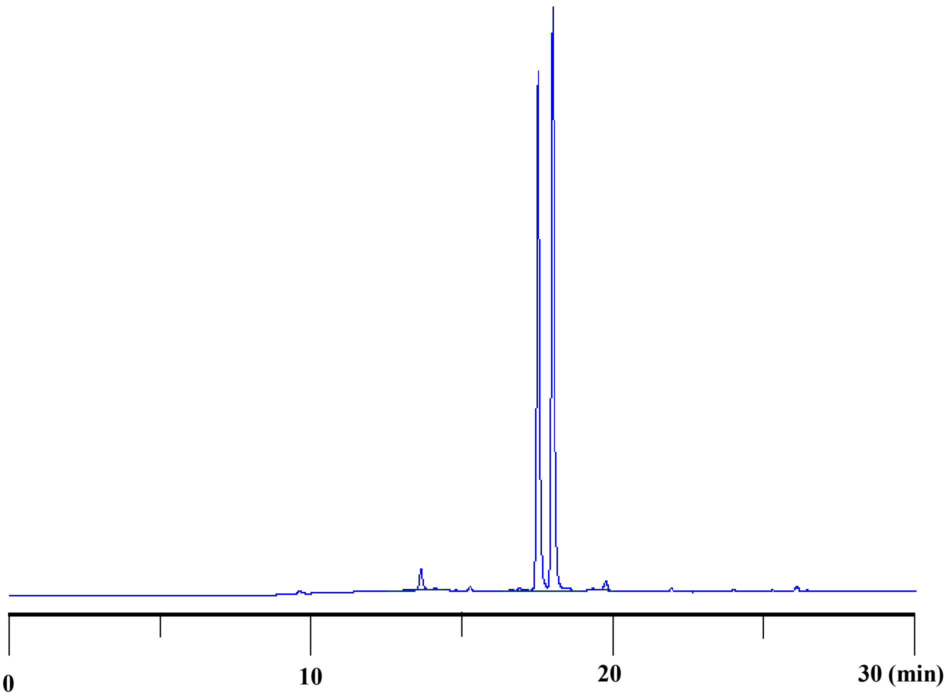

Figure S33 RP-HPLC profiles of dC$_{PS}$T dimer in **Table 2**, Entry 4

RP-HPLC (C18 (5 µm, 100 Å, 3.9 × 150 mm), 0.1 M triethylammonium acetate buffer (pH 7.0)/MeCN = 100/0–70/30 over 30 min, flow rate = 0.5 mL/min, l = 260 nm, t = 30°C) tR = 17.6, 18.1 min (diastereomers)

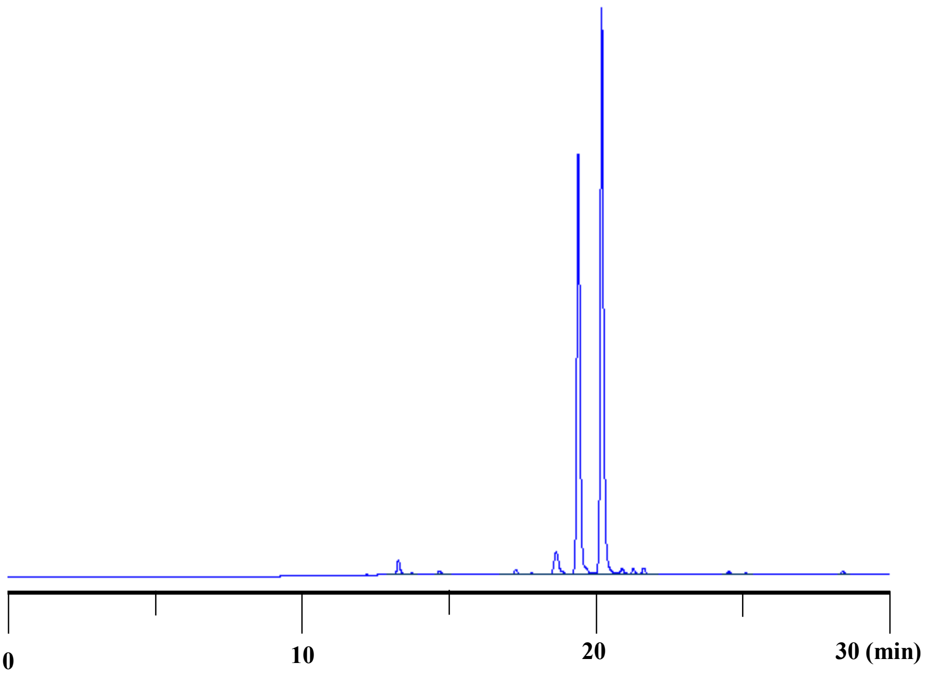

Figure S34 RP-HPLC profiles of dA$_{PS}$T dimer in **Table 2**, Entry 5

RP-HPLC (C18 (5 µm, 100 Å, 3.9 × 150 mm), 0.1 M triethylammonium acetate buffer (pH 7.0)/MeCN = 100/0–70/30 over 30 min, flow rate = 0.5 mL/min, l = 260 nm, t = 30°C) tR = 19.5, 20.3 min (diastereomers)

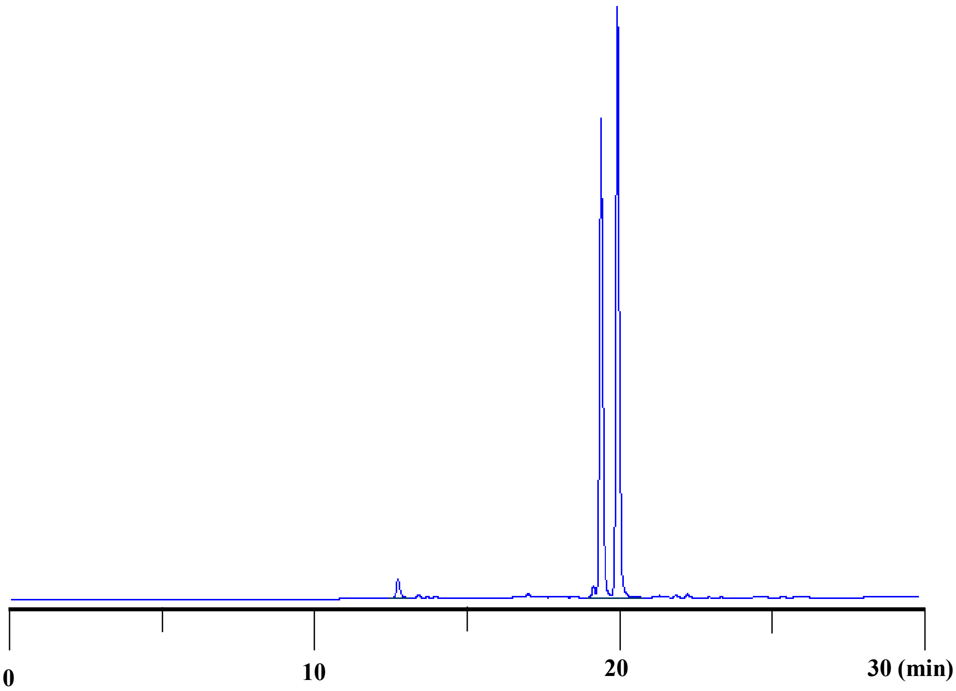

Figure S35 RP-HPLC profiles of T$_{PS}$T dimer in **Table 2**, Entry 6

RP-HPLC (C18 (5 µm, 100 Å, 3.9 × 150 mm), 0.1 M triethylammonium acetate buffer (pH 7.0)/MeCN = 100/0–70/30 over 30 min, flow rate = 0.5 mL/min, l = 260 nm, t = 30°C) tR = 19.7, 20.2 min (diastereomers)

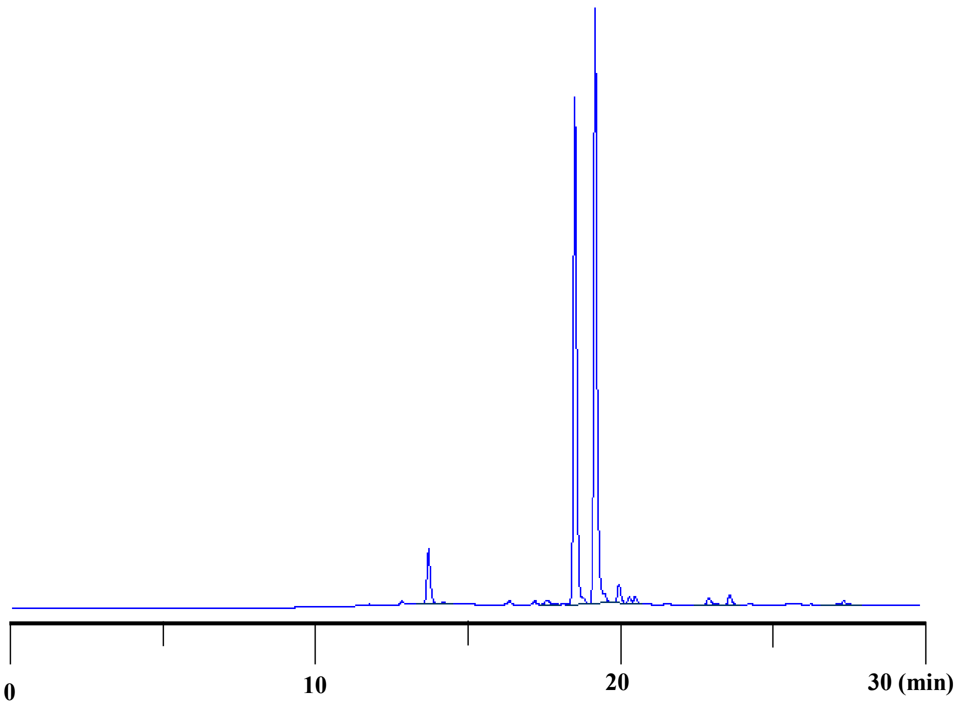

Figure S36 RP-HPLC profiles of dG$_{PS}$T dimer in **Table 2**, Entry 7

RP-HPLC (C18 (5 µm, 100 Å, 3.9 × 150 mm), 0.1 M triethylammonium acetate buffer (pH 7.0)/MeCN = 100/0–70/30 over 30 min, flow rate = 0.5 mL/min, l = 260 nm, t = 30°C) tR = 18.3, 19.0 min (diastereomers)

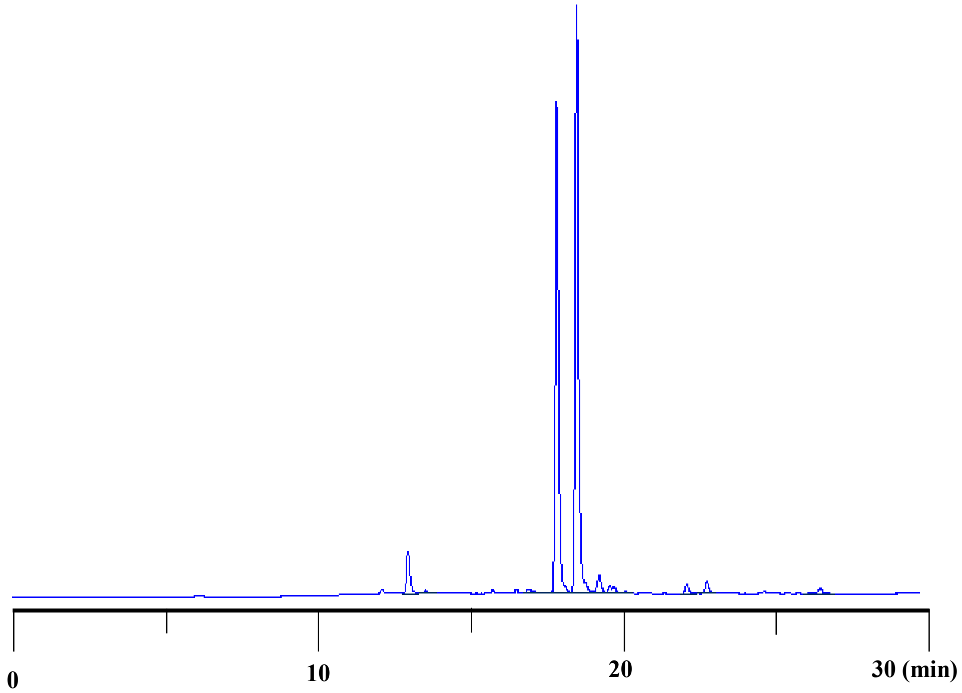

Figure S37 RP-HPLC profiles of dG$_{PS}$T dimer in **Table 2**, Entry 8

RP-HPLC (C18 (5 µm, 100 Å, 3.9 × 150 mm), 0.1 M triethylammonium acetate buffer (pH 7.0)/MeCN = 100/0–70/30 over 30 min, flow rate = 0.5 mL/min, l = 260 nm, t = 30°C) tR = 18.1, 18.8 min (diastereomers)

RP-HPLC profiles of N$_{PS2}$T dimer

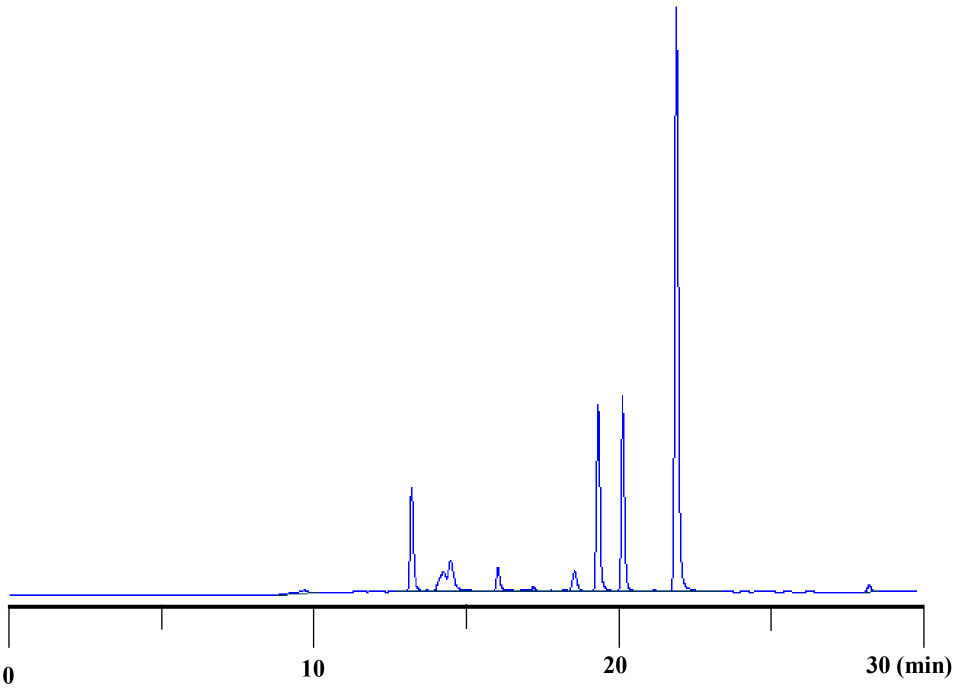

Figure S38 RP-HPLC profiles of dA$_{PS2}$T dimer in **Table 3**, Entry 1

RP-HPLC (C18 (5 µm, 100 Å, 3.9 × 150 mm), 0.1 M triethylammonium acetate buffer (pH 7.0)/MeCN = 100/0–70/30 over 30 min, flow rate = 0.5 mL/min, l = 260 nm, t = 30°C) tR = 22.1 min

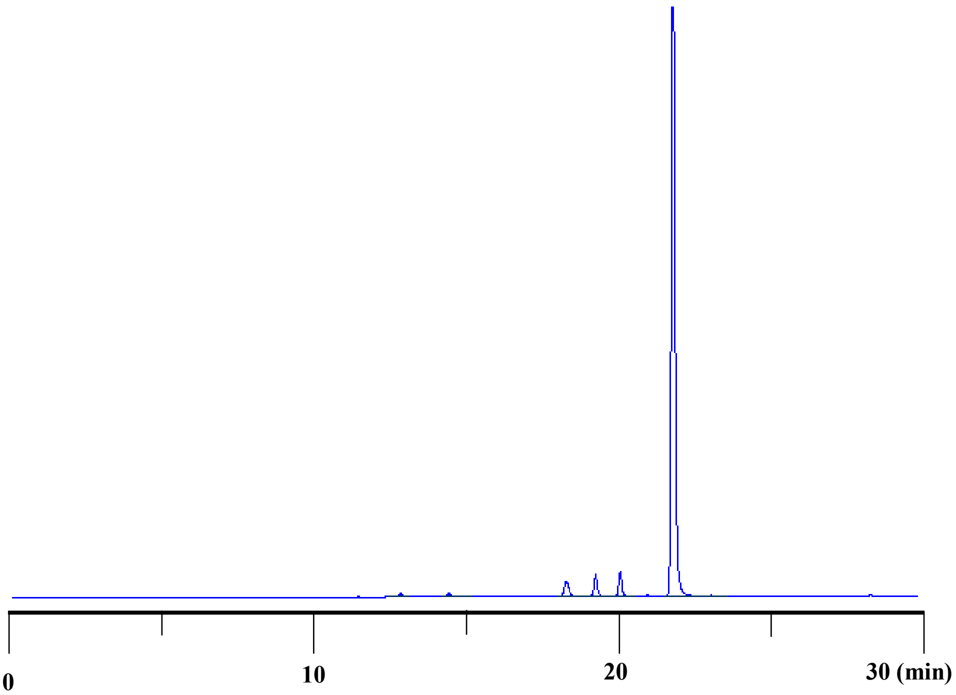

Figure S39 RP-HPLC profiles of dA$_{PS2}$T dimer in **Table 3**, Entry 2

RP-HPLC (C18 (5 µm, 100 Å, 3.9 × 150 mm), 0.1 M triethylammonium acetate buffer (pH 7.0)/MeCN = 100/0–70/30 over 30 min, flow rate = 0.5 mL/min, l = 260 nm, t = 30°C) tR = 22.1 min

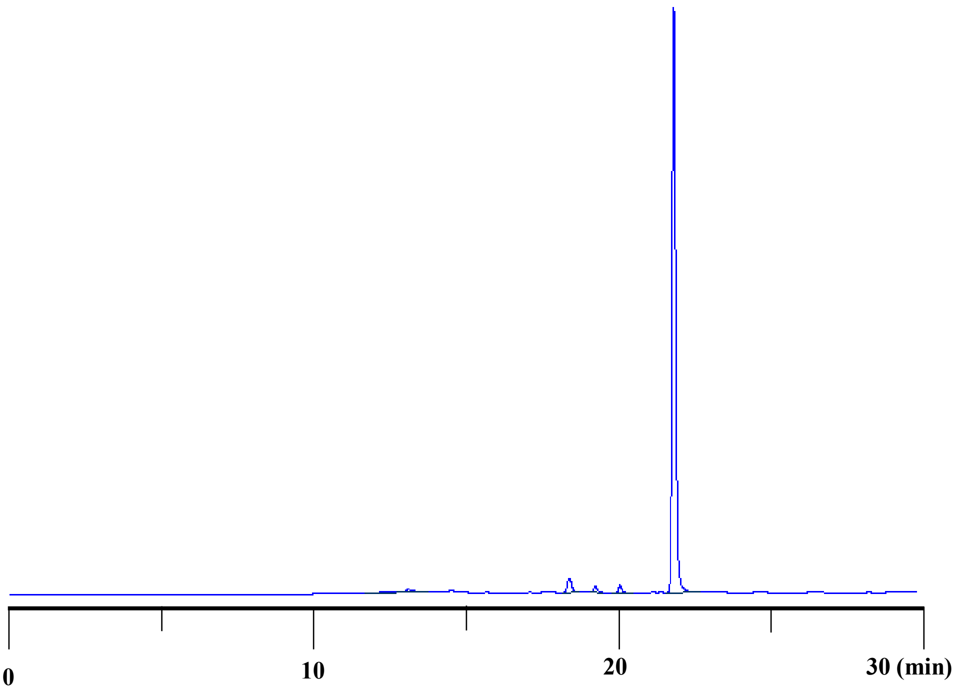

Figure S40 RP-HPLC profiles of dA$_{PS2}$T dimer in **Table 3**, Entry 3

RP-HPLC (C18 (5 µm, 100 Å, 3.9 × 150 mm), 0.1 M triethylammonium acetate buffer (pH 7.0)/MeCN = 100/0–70/30 over 30 min, flow rate = 0.5 mL/min, l = 260 nm, t = 30°C) tR = 22.1 min

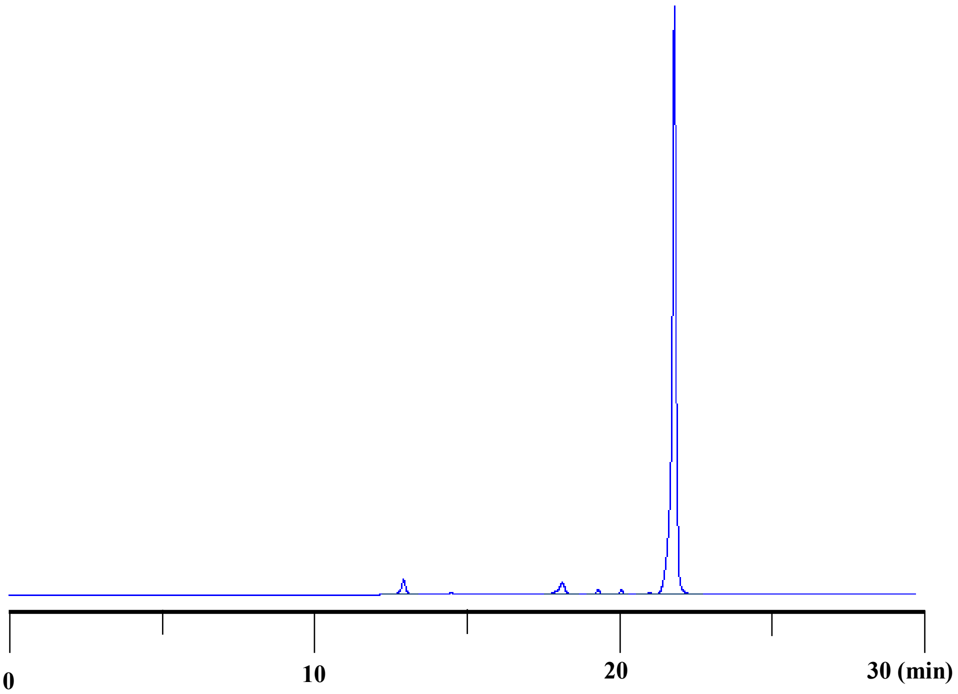

Figure S41 RP-HPLC profiles of dA$_{PS2}$T dimer in **Table 3**, Entry 4

RP-HPLC (C18 (5 µm, 100 Å, 3.9 × 150 mm), 0.1 M triethylammonium acetate buffer (pH 7.0)/MeCN = 100/0–70/30 over 30 min, flow rate = 0.5 mL/min, l = 260 nm, t = 30°C) tR = 22.1 min

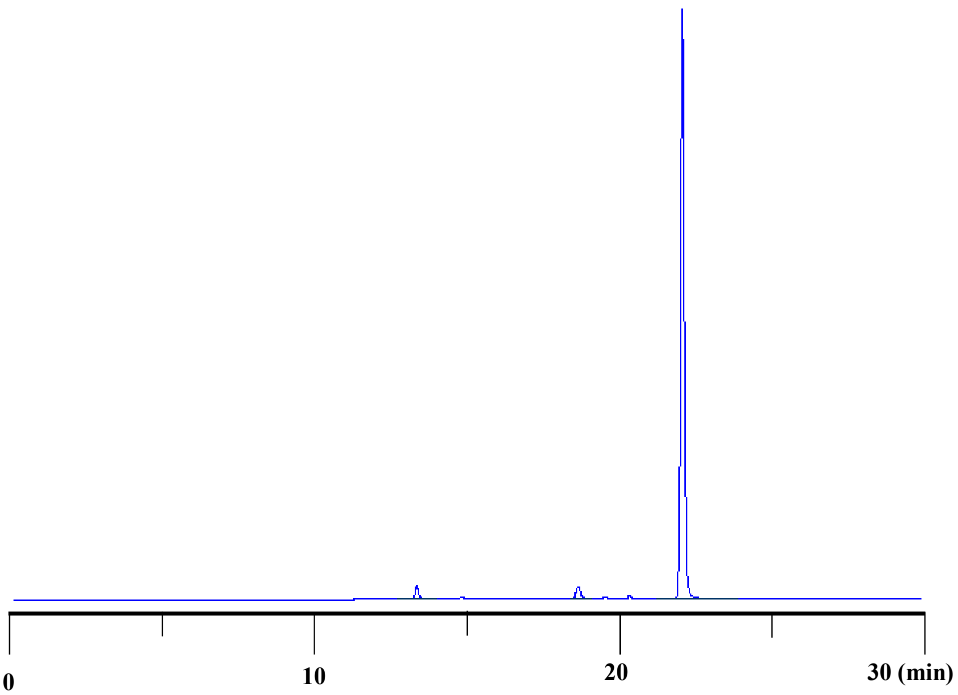

Figure S42 RP-HPLC profiles of dA$_{PS2}$T dimer in **Table 3**, Entry 5

RP-HPLC (C18 (5 µm, 100 Å, 3.9 × 150 mm), 0.1 M triethylammonium acetate buffer (pH 7.0)/MeCN = 100/0–70/30 over 30 min, flow rate = 0.5 mL/min, l = 260 nm, t = 30°C) tR = 22.2 min

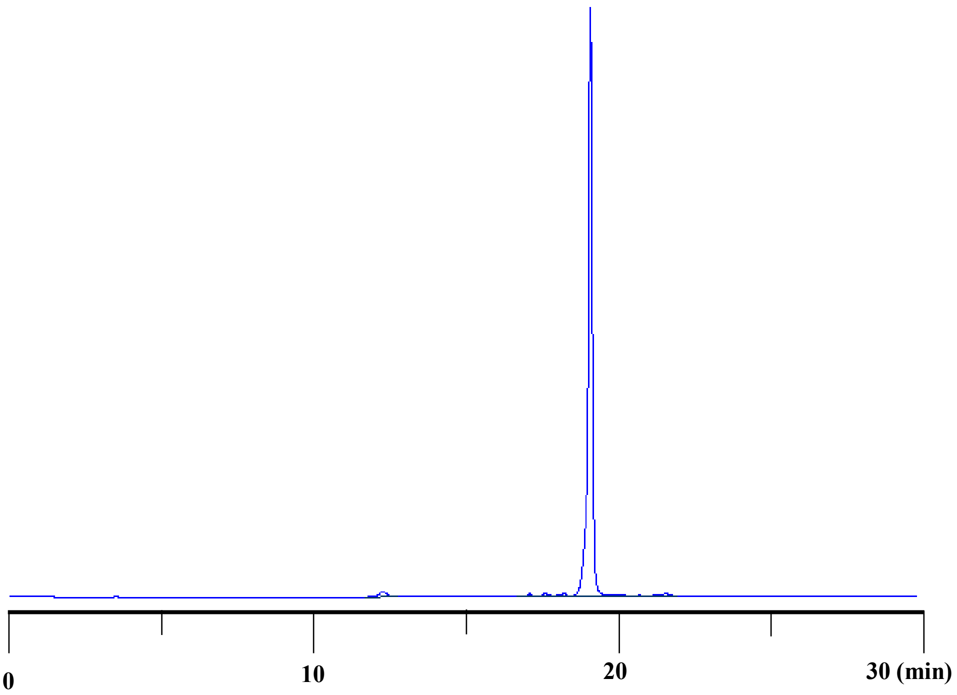

Figure S43 RP-HPLC profiles of dC$_{PS2}$T dimer in **Table 3**, Entry 6

RP-HPLC (C18 (5 µm, 100 Å, 3.9 × 150 mm), 0.1 M triethylammonium acetate buffer (pH 7.0)/MeCN = 100/0–70/30 over 30 min, flow rate = 0.5 mL/min, l = 260 nm, t = 30°C) tR = 19.3 min

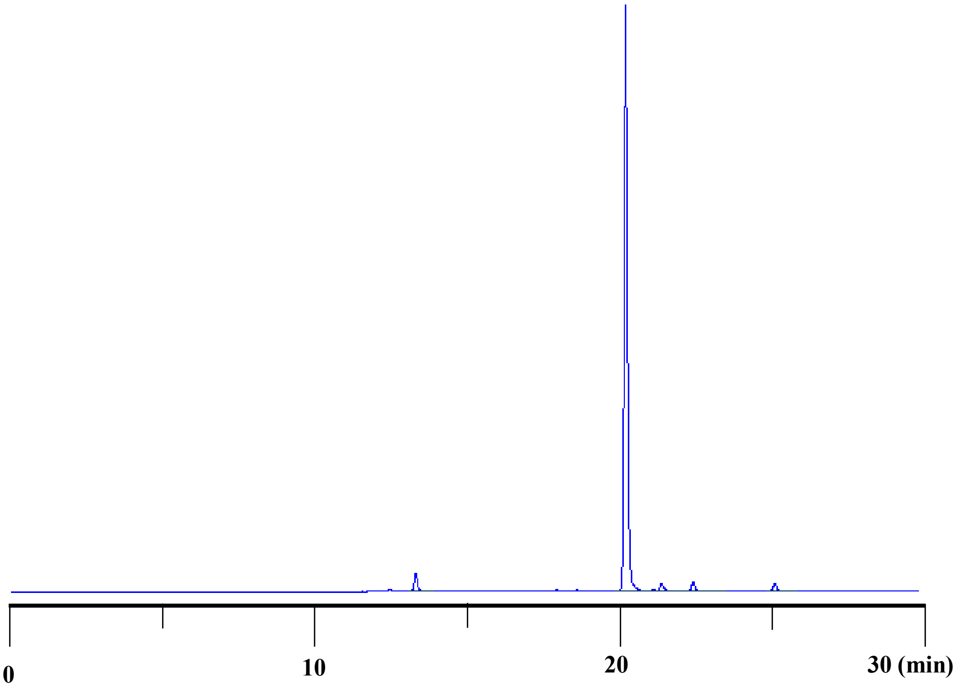

Figure S44 RP-HPLC profiles of $dG_{PS2}T$ dimer in **Table 3**, Entry 7

RP-HPLC (C18 (5 µm, 100 Å, 3.9 × 150 mm), 0.1 M triethylammonium acetate buffer (pH 7.0)/MeCN = 100/0–70/30 over 30 min, flow rate = 0.5 mL/min, l = 260 nm, t = 30°C) tR = 20.4 min

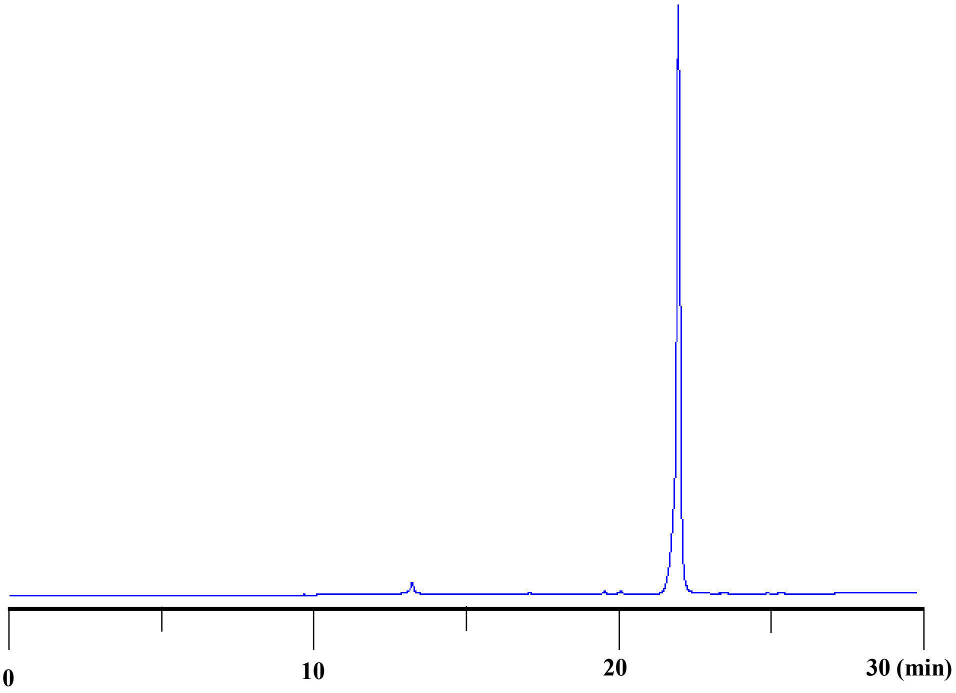

Figure S45 RP-HPLC profiles of $T_{PS2}T$ dimer in **Table 3**, Entry 8

RP-HPLC (C18 (5 µm, 100 Å, 3.9 × 150 mm), 0.1 M triethylammonium acetate buffer (pH 7.0)/MeCN = 100/0–70/30 over 30 min, flow rate = 0.5 mL/min, l = 260 nm, t = 30°C) tR = 22.2 min

Desulfurization by PyNTP in the solid-phase synthesis

The results presented in **Table 3**, Entry 1 indicated a notable decrease in chemoselectivity when PyNTP was employed on NittoPhase^{TM} HL. However, this finding appears inconsistent with our previous report, where *H*-phosphonothioate diester linkages were obtained with high chemoselectivity on HCP. According to the data from **Table 2**, the situation of using NittoPhase^{TM} HL as a solid support might be closer to the pre-activation protocol than using HCP. Thus, we hypothesized that the pre-activation of an *H*-phosphonothioate monoester by PyNTP caused desulfurization, resulting the formation of *H*-phosphonate diester linkages. Consequently, to prove the hypothesis, we examined the influence of the pre-activation protocol with PyNTP in liquid-phase synthesis.

Scheme S7

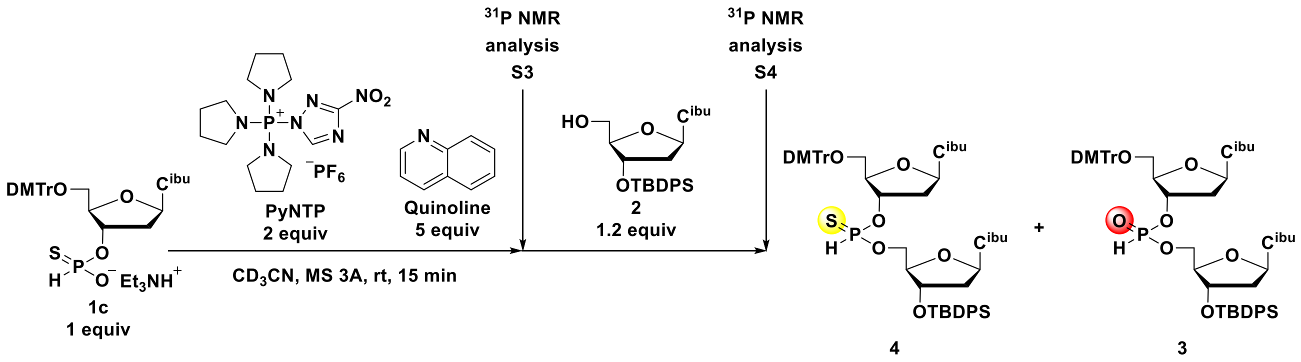

A pre-activation protocol with PyNTP was investigated as follows: Deoxycytidine 3′-*H*-phosphonothioate monoester derivative **1c** and 5 equiv of quinoline as a base were dissolved in dry CD_3CN and thoroughly dried over MS 3A, followed by the addition of 2 equiv of PyNTP. After 15 min, the reaction mixture was analyzed by ^{31}P NMR (**Figure S46, ^{31}P NMR spectrum S3**). Subsequently, 1.2 equiv of nucleoside **2** bearing a free 5′-hydroxy group was added and after 15 min, the mixture was analyzed by ^{31}P NMR (**Figure S46, ^{31}P NMR spectrum S4**). The NMR yield was calculated as the integral ratio of the signals corresponding to the desired *H*-phosphonothioate diester **4** (δ 71–73) and all signals, and the chemoselectivity was estimated on the basis of the integral ratio of the desired *H*-phosphonothioate diester **4** to the undesired *H*-phosphonate diester **3** (δ 8–10).

As a result, the signals corresponded to desired *H*-phosphonothioate diester **4** and the undesired *H*-phosphonate diester **3** were observed in ^{31}P NMR (NMR yield of **4** 6%, **3**:**4** = 37:63). The chemoselectivity was similar to that of solid-phase synthesis (**Table 3**, Entry 1, **11**:**12** = 38:62). Thus, consistent with our initial hypothesis, the pre-activation protocol with PyNTP is similar to the situation of solid-phase synthesis on NittoPhase^{TM} HL and these situations caused desulfurization and the formation of the undesired *H*-phosphonate diester **3**.

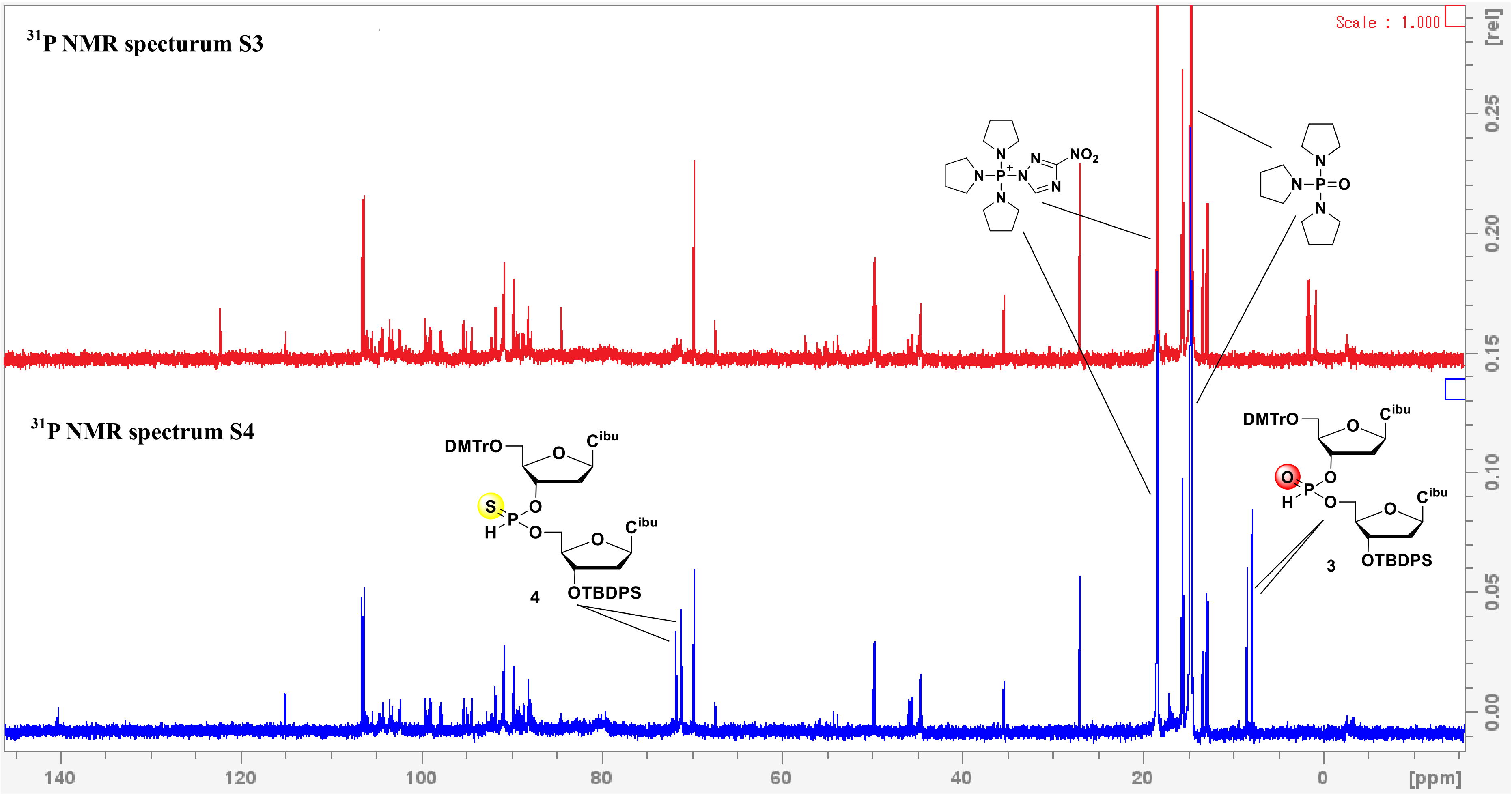

Figure S46 $^{31}P\{^{1}H\}$ NMR spectra of **Scheme S7** (202 MHz, in CD_3CN)

RP-HPLC profiles of purified PS_2/PS chimeric pentamer (13)

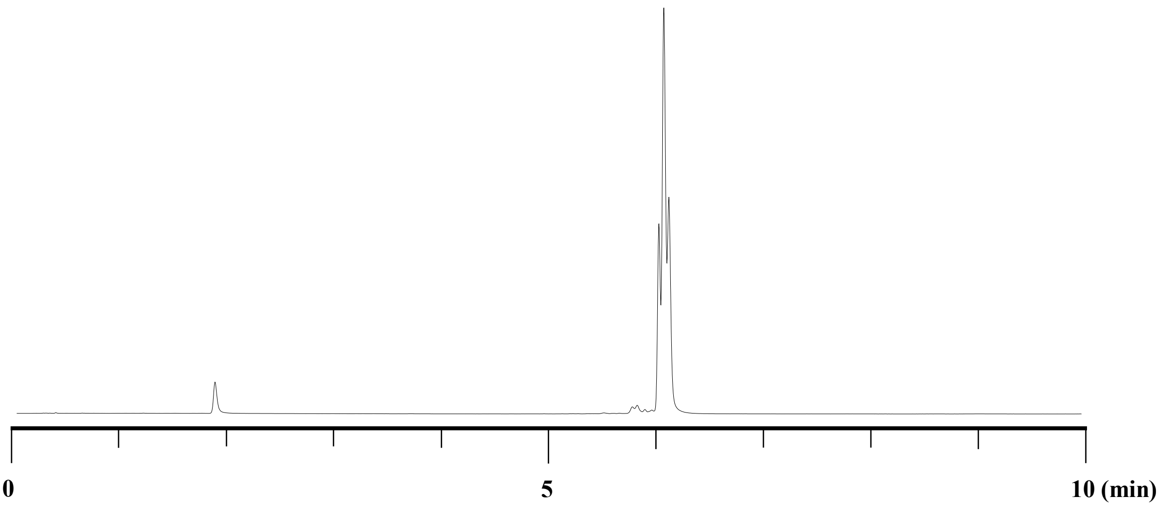

Figure S47 RP-UPLC profiles of purified PS_2/PS chimeric pentamer (d($T_{PS}C_{PS2}A_{PS}G_{PS2}T$)) (**13**)

RP-UPLC (C18 (1.7 µm, 130 Å, 2.1 × 50 mm), solution A/solution B = 99/1–65/35 over 10 min, flow rate = 0.6 mL/min, l = 260 nm, t = 45°C) tR = 6.08, 6.12, 6.17 min (diastereomers)

Solution A: 50 mM hexafluoroisopropanol (HFIP), 5 mM hexylamine (HA) aq

Solution B: Solution A–MeCN (1:1, v/v)

RP-HPLC profiles of purified PS_2/PS chimeric dodecamer (14)

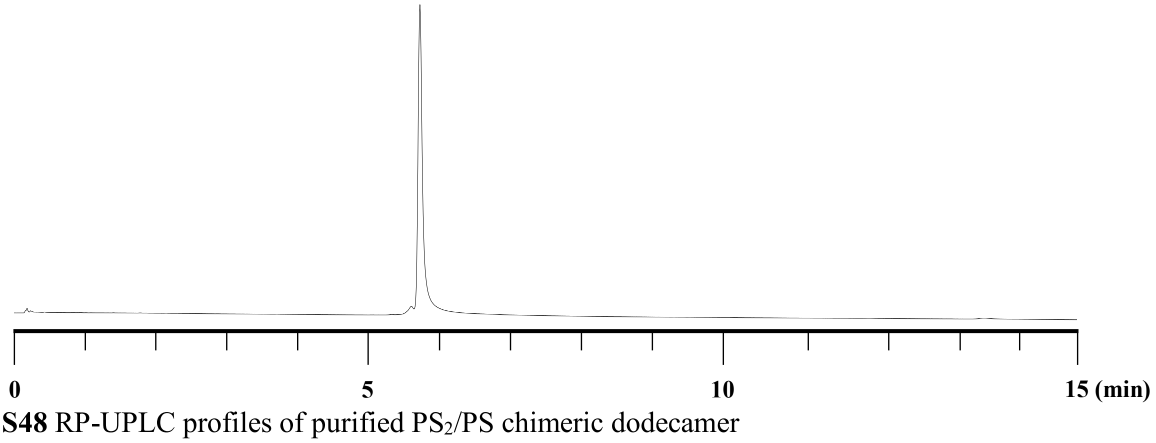

Figure S48 RP-UPLC profiles of purified PS_2/PS chimeric dodecamer (d($C_{PS2}A_{PS}G_{PS2}T_{PS}C_{PS}A_{PS2}G_{PS}T_{PS2}C_{PS2}A_{PS}G_{PS2}T$)) (**14**)

RP-UPLC (C18 (1.7 µm, 130 Å, 2.1 × 50 mm), solution C/solution D = 95/5–75/25 over 15 min, flow rate = 0.6 mL/min, l = 260 nm, t = 60°C) tR = 5.78 min

Solution C: 100 mM hexafluoroisopropanol (HFIP) and 8 mM triethylamine aq

Solution D: Solution C–MeCN (1:1, v/v)

Analysis of PS_2/PS chimeric pentamer and dodecamer by LC-MS/MS

Although these synthesized oligonucleotides were indicated that have same *m/z* values as the desired oligonucleotides by RP-UPLC-MS analysis, it was not proved that these modifications were successfully introduced at the intended position. Thus, we confirmed the modifications of the oligonucleotides by LC-MS/MS and CONFIRM Sequence App analysis.

For the pentamer, information from *m/z* values of fragment ions indicates that both the nucleobase sequence and the types of internucleotidic linkages of the pentamer were identical to the theoretical ones (**Figures S49–S52**).

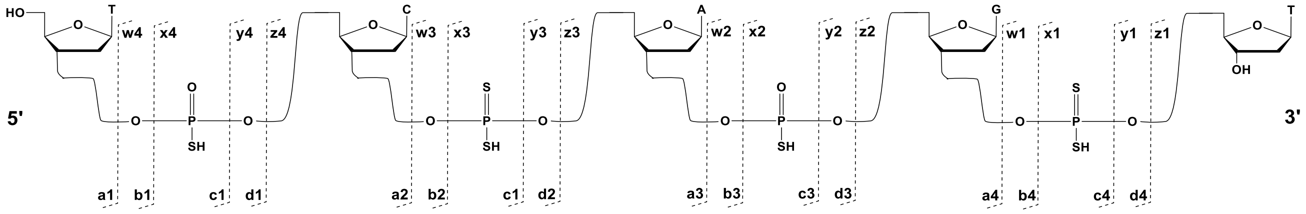

Figure S49 Structure of fragment ions

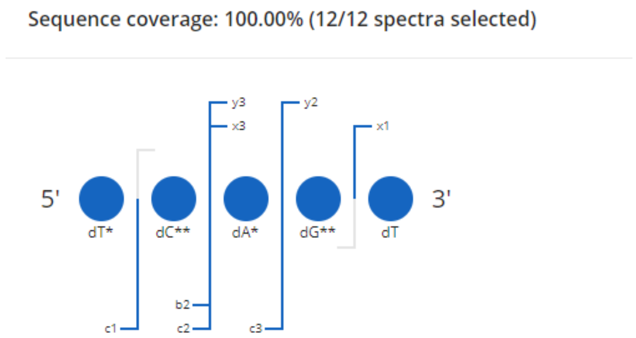

Figure S50 The list of fragment ions (* denotes PS linkages, ** denotes PS_2 linkages)

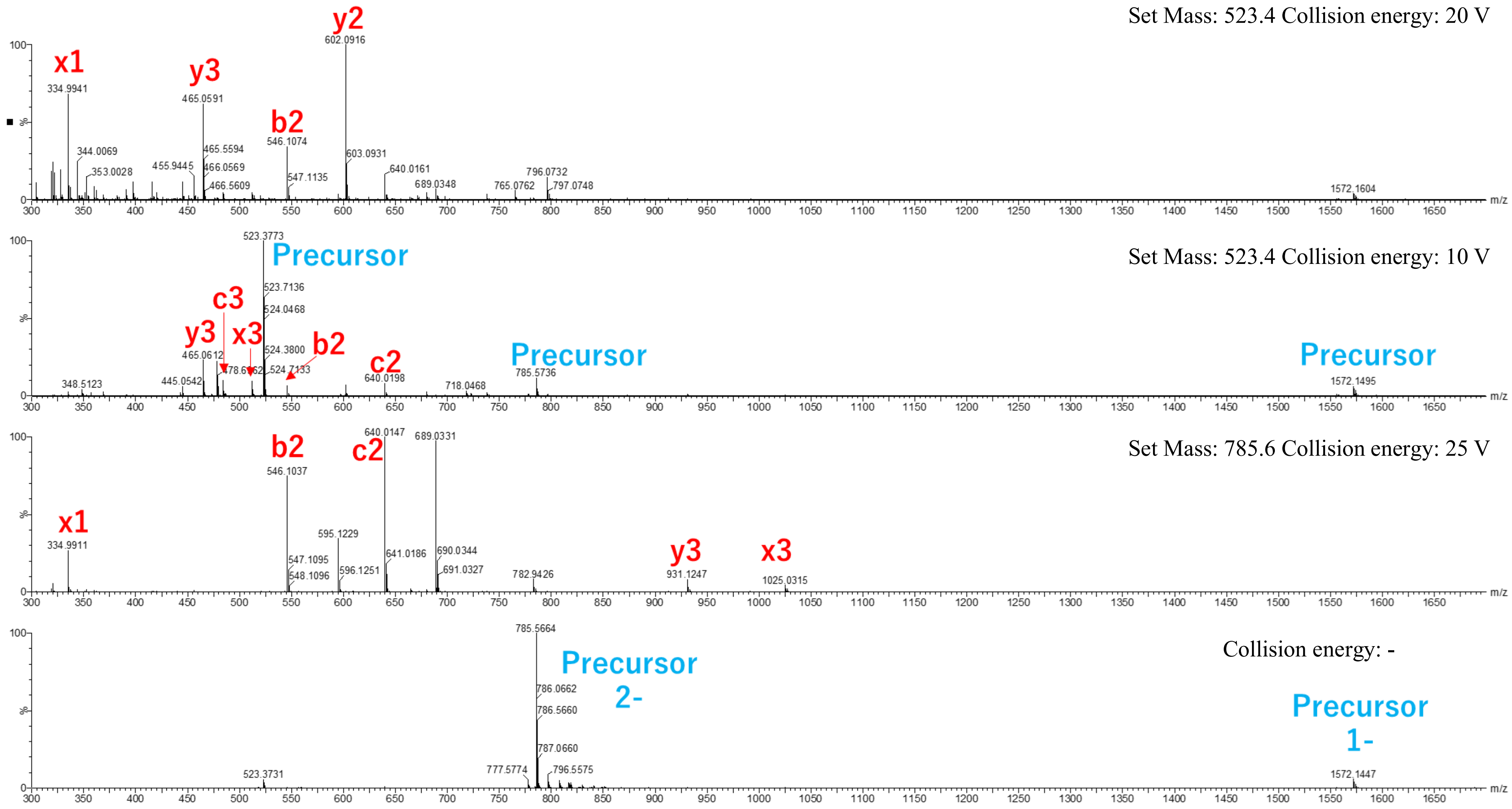

Figure S51 MS/MS spectra of the PS_2/PS chimeric pentamer

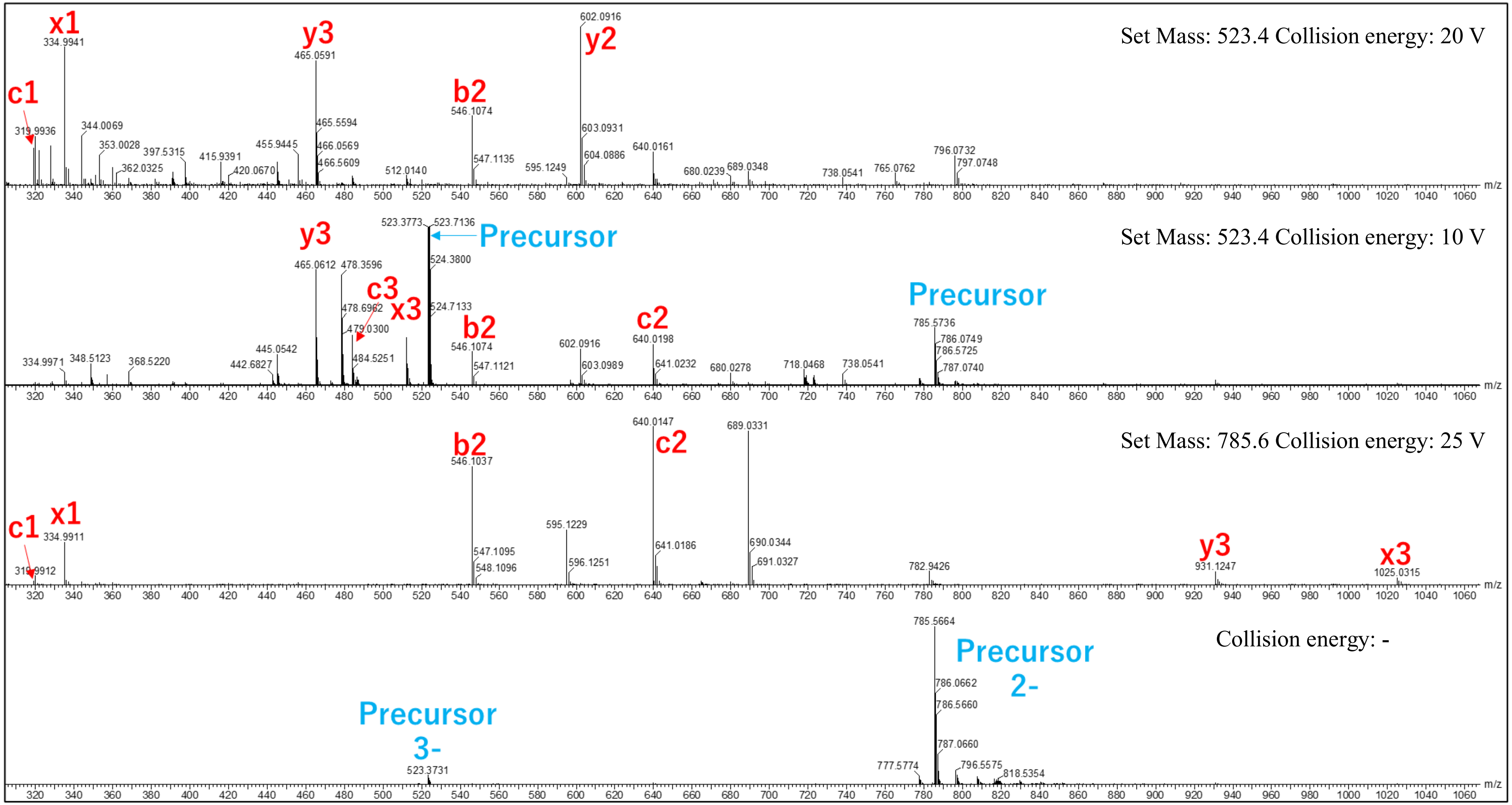

Figure S52 MS/MS spectra of the PS_2/PS chimeric pentamer (expansion)

Similarly, for the dodecamer, we successfully observed the *m/z* value corresponding to the target structure. This suggested that nucleobases and phosphorodithioate (PS_2) and phosphorothioate (PS) linkages were introduced with the anticipated numbers. However, the sequence of the central region of the oligonucleotide could not be unambiguously determined.

Assuming the nucleobase sequence was correct, the following analysis results indicated that the desired internucleotidic modifications were introduced at the intended positions (**Figures S53–S57**): First, the sequence of the nucleobases, and the position of the internucleotidic linkages, are definitively confirmed for the three bases from the 5'-end and the four bases from the 3'-end (indicated by blue circles). Next, the *m/z* value of the x4 fragment indicates that the fourth internucleotidic linkage from the 3′-end is a PS_2 modification. This, in turn, reveals that the fifth internucleotidic linkage from the 3′-end is a PS modification, based on the *m/z* value of the y6 fragment. Furthermore, the difference of *m/z* values of the b6 and c6 fragments confirms that the sixth internucleotidic linkage from the 3′-end is a PS_2 modification. Then, the presence of the x9 fragment suggests that the seventh and eighth internucleotidic linkages from the 3′-end are PS modifications. Thus, we can unambiguously confirm that the synthesized oligonucleotide possesses the desired modifications at intended positions.

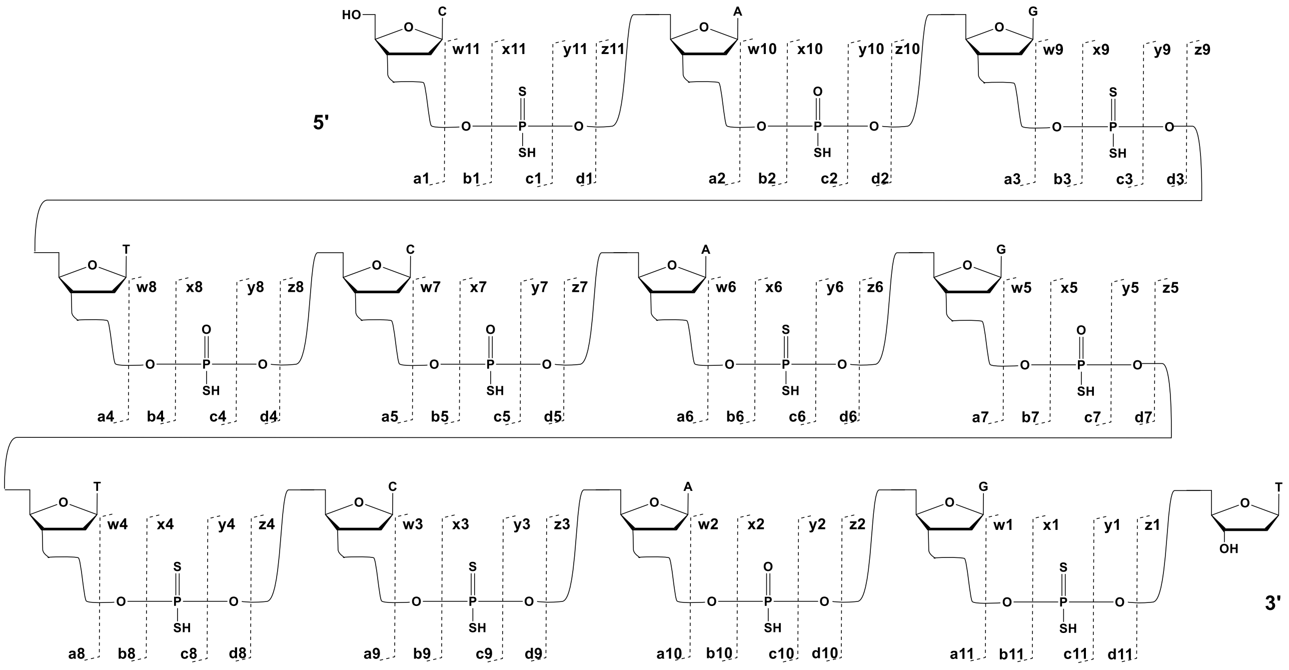

Figure S53 Structure of fragment ions

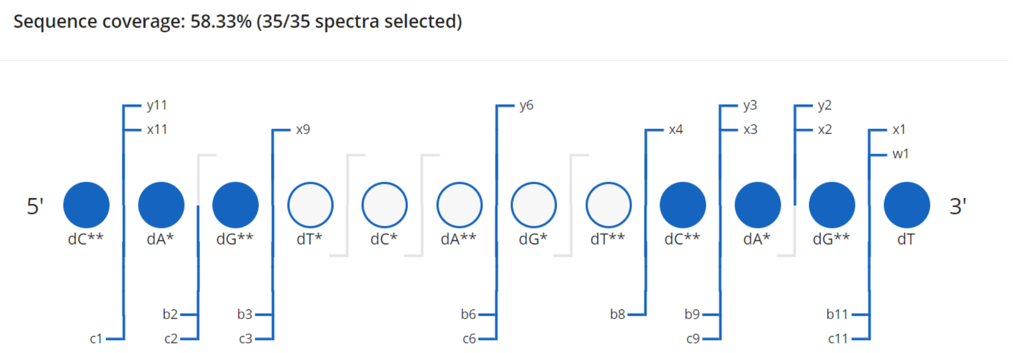

Figure S54 The list of fragment ions (* denotes PS linkages, ** denotes PS_2 linkages)

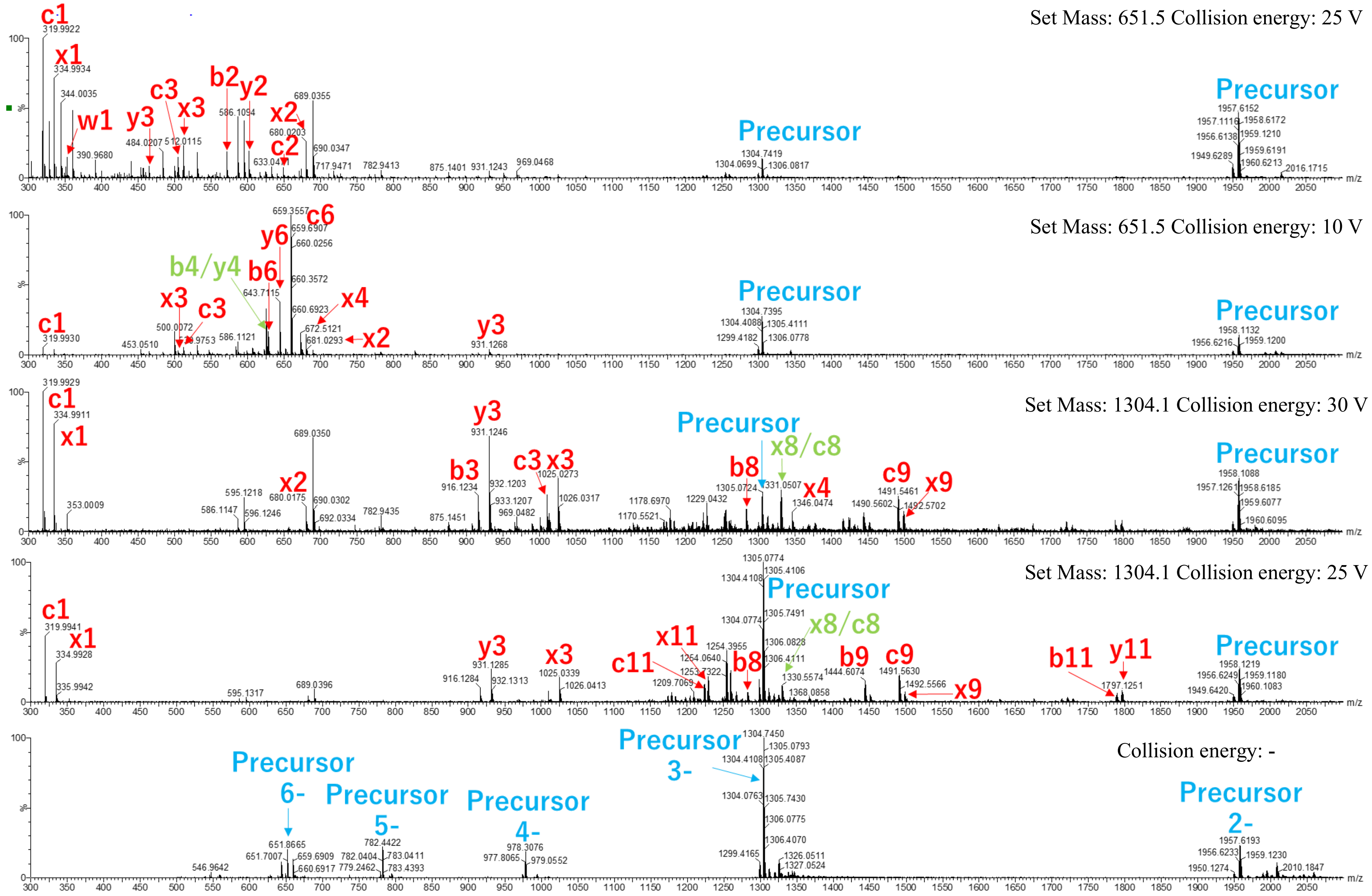

Figure S55 MS/MS spectra of the PS_2/PS chimeric dodecamer

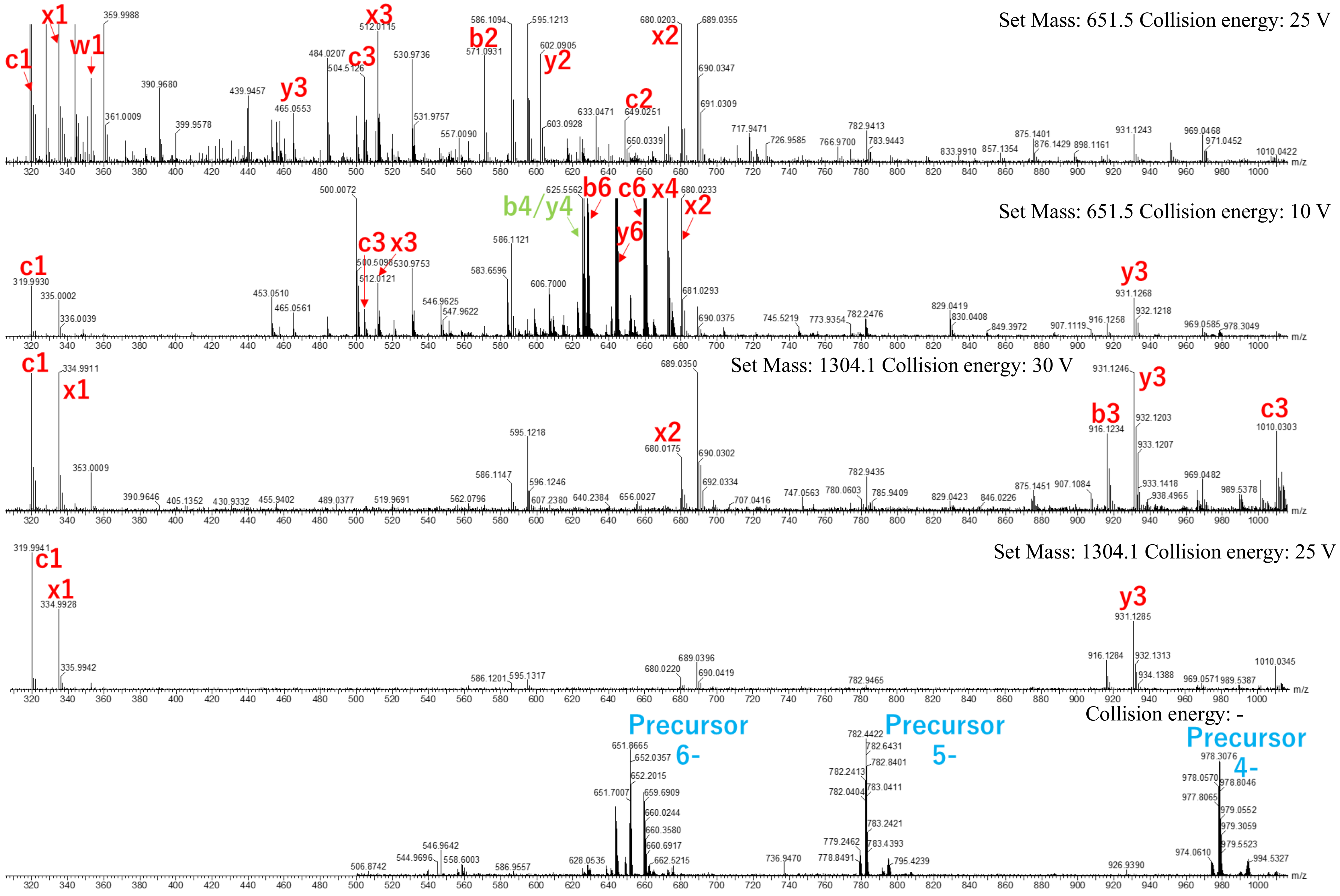

Figure S56 MS/MS spectra of the PS_2/PS chimeric dodecamer (expansion)

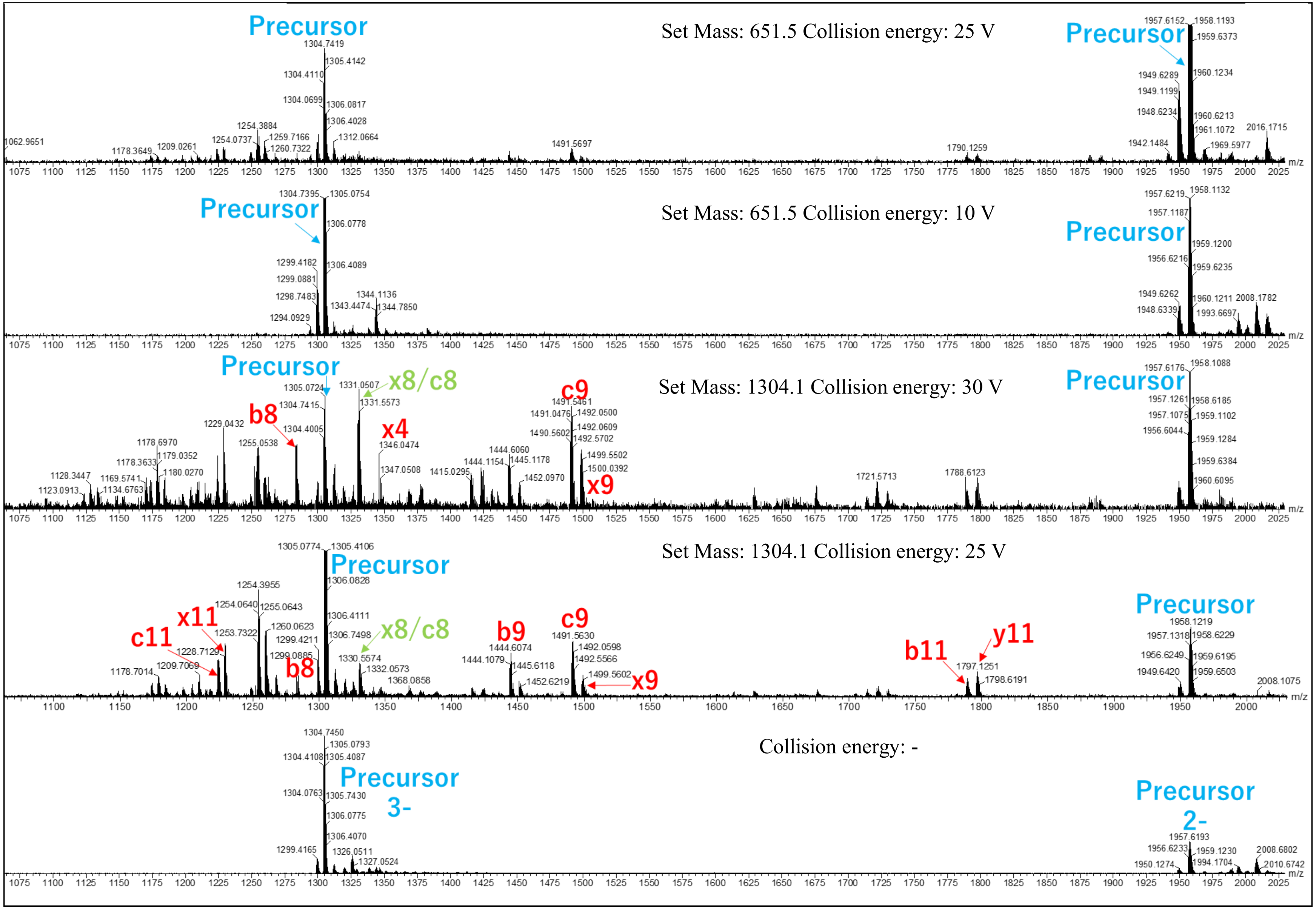

Figure S57 MS/MS spectra of the PS_2/PS chimeric dodecamer (expansion)

Investigation of synthesis of PSN and PN dimers

To investigate optimal reaction conditions on NittoPhase™ HL, the synthesis of dinucleosides PSN and PN was conducted as follows: Deoxycytidine *H*-phosphonothioate monomer **1c** was reacted with a 5′-hydroxy group of thymidine loaded on NittoPhase™ HL under the conditions of forming *H*-phosphonate or *H*-phosphonothioate diesters. Then, the DMTr group on the 5′-end was removed and the internucleotidic linkage was oxidatively aminated by the conditions (**Table S3**). After treatment with concentrated aqueous NH_3–EtOH (3:1, v/v; 5 mL) for cleavage of the linker and deprotection of nucleobases, the reaction mixture was analyzed by RP-HPLC (**Scheme S8**), and HPLC yield and chemoselectivity were determined by the area ratios of the dinucleosides phosphoramidate **S6** and phosphorothioamidate **S7**.

Scheme S8

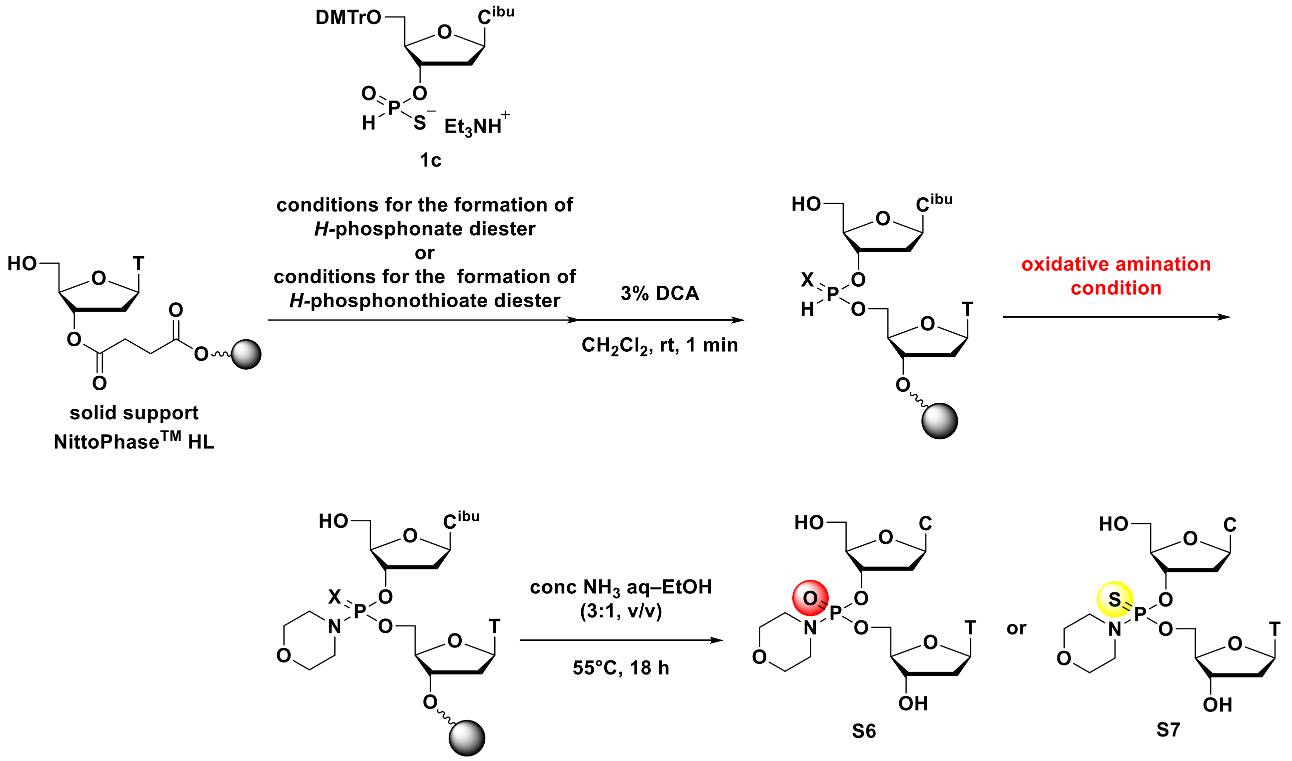

Condensation conditions for the formation of the *H*-phosphonate diester: 0.1 M deoxycytidine-3′-*H*-phosphonothioate monomer **1c**, 0.2 M DIC, 0.2 M CMPT, MeCN, rt, 15 min (pre-activation protocol)

Condensation conditions for the formation of the *H*-phosphonothioate diester: 0.1 M deoxycytidine-3′-*H*-phosphonothioate monomer **1c**, 0.15 M DIPC, MeCN (containing 10% v/v pyridine), rt, 5 min

Table S3

Entry	Product	Conditions	HPLC yield (%) a)	Chemoselectivity **S6**:**S7** b)
1	dC$_{\mathrm{PN}}$T (**S6**)	0.05 M I_2<br>pyridine–morpholine (92:8, v/v), rt, 5 min	55	>99:1
2	dC$_{\mathrm{PN}}$T (**S6**)	1.0 M CBr_4<br>MeCN–morpholine (92:8, v/v), rt, 5 min	62	>99:1
3	dC$_{\mathrm{PN}}$T	CCl_4–MeCN–morpholine (50:414:36, v/v/v)	78	>99:1

	(**S6**)	rt, 5 min		
4	dC$_{PSN}$T (**S7**)	CCl_4–MeCN–morpholine (50:414:36, v/v/v) rt, 5 min	97	1:>99
5	dC$_{PN}$T (**S6**)	CCl_4–MeCN–morpholine (4.5:4.5:1, v/v/v) rt, 5 min	84	>99:1
6	dC$_{PSN}$T (**S7**)	CCl_4–MeCN–morpholine (4.5:4.5:1, v/v/v) rt, 5 min	98	1:>99

[a] Determined by the crude RP-HPLC area ratio of dC$_{PSN}$T or dC$_{PN}$T.

[b] Determined by the crude RP-HPLC area ratios dC$_{PN}$T (**S6**):dC$_{PSN}$T (**S7**).

In **Table S3**, Entries 1–3, halogenation reagents were investigated. The use of I_2 (Entry 1) or CBr_4 (Entry 2) indicated insufficient conversion, giving the targeted phosphoramidate linkage in moderate HPLC yield (55% and 62%, Entries 1 and 2, respectively). Next, the use of 10% v/v of CCl_4 afforded the highest conversion and the desired phosphoramidate dimer was obtained in 78% HPLC yield with complete chemoselectivity (Entry 3). Additionally, in the case of the using 45% v/v of CCl_4, the desired phosphoramidate (Entry 5) and phosphorothioamidate (Entry 6) dimers were successfully obtained in 84% and 98% HPLC yield, respectively, with complete chemoselectivity. The HPLC profiles of the investigations were shown below (**Figure S58–S63**).

HRMS (ESI/Q-TOF) *m/z*: $[M+H]^+$ Calcd for $C_{23}H_{34}N_6O_{11}P^+$ (**S6**) 601.2018; Found 601.2023. $[M+H]^+$ Calcd for $C_{23}H_{34}N_6O_{10}PS^+$ (**S7**) 617.1789; Found 617.1796.

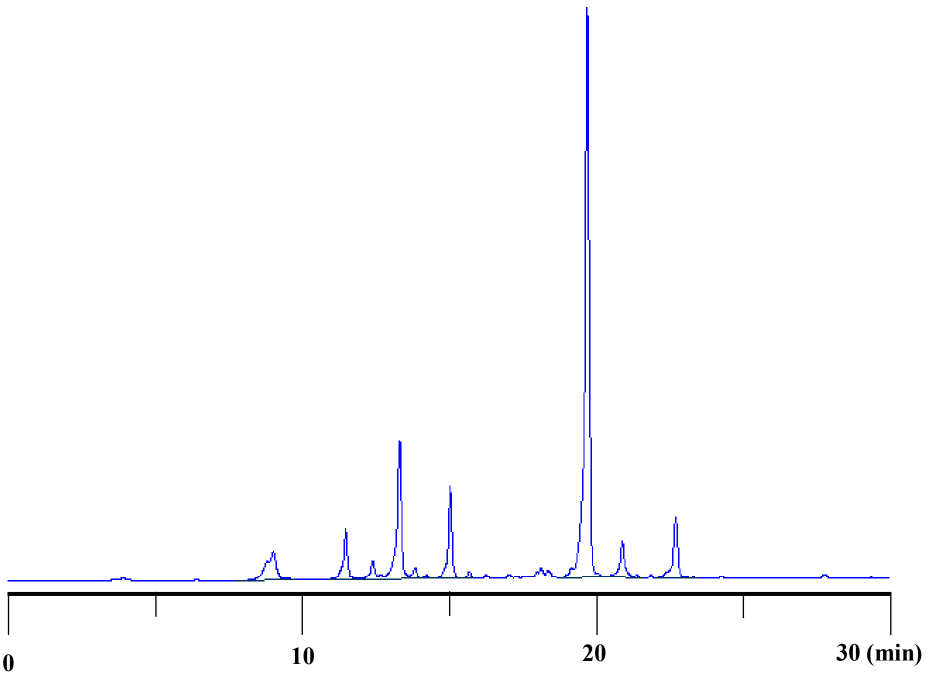

Figure S58 RP-HPLC profiles of dC$_{PN}$T dimer in **Table S3,** Entry 1

RP-HPLC (C18 (5 μm, 100 Å, 3.9 × 150 mm), 0.1 M triethylammonium acetate buffer (pH 7.0)/MeCN = 100/0–70/30 over 30 min, flow rate = 0.5 mL/min, l = 260 nm, t = 30°C) tR = 19.8 min

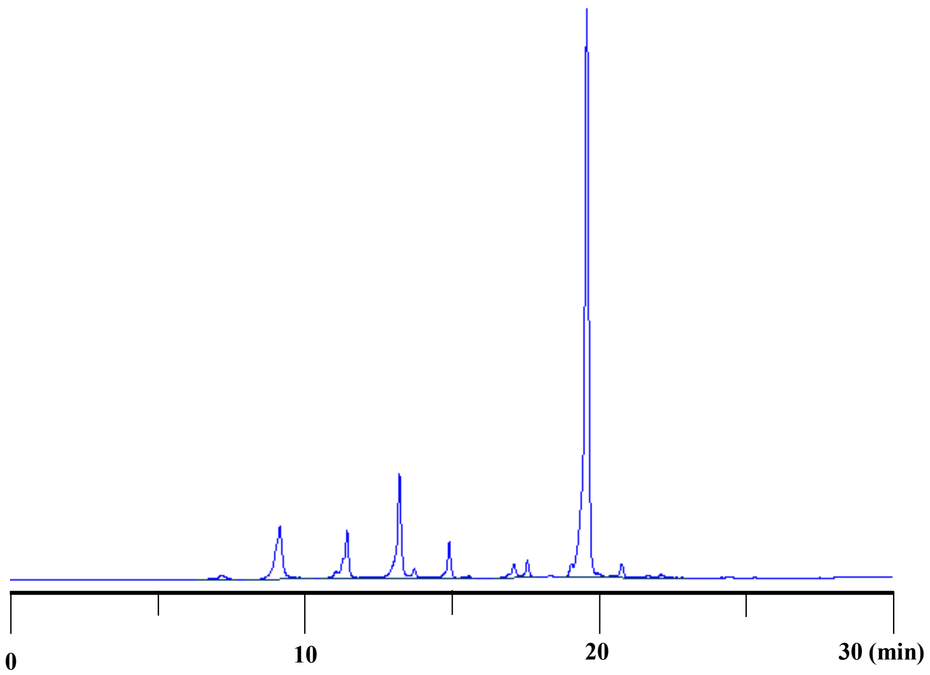

Figure S59 RP-HPLC profiles of dC$_{PN}$T dimer in **Table S3**, Entry 2

RP-HPLC (C18 (5 µm, 100 Å, 3.9 × 150 mm), 0.1 M triethylammonium acetate buffer (pH 7.0)/MeCN = 100/0–70/30 over 30 min, flow rate = 0.5 mL/min, l = 260 nm, t = 30°C) tR = 19.7 min

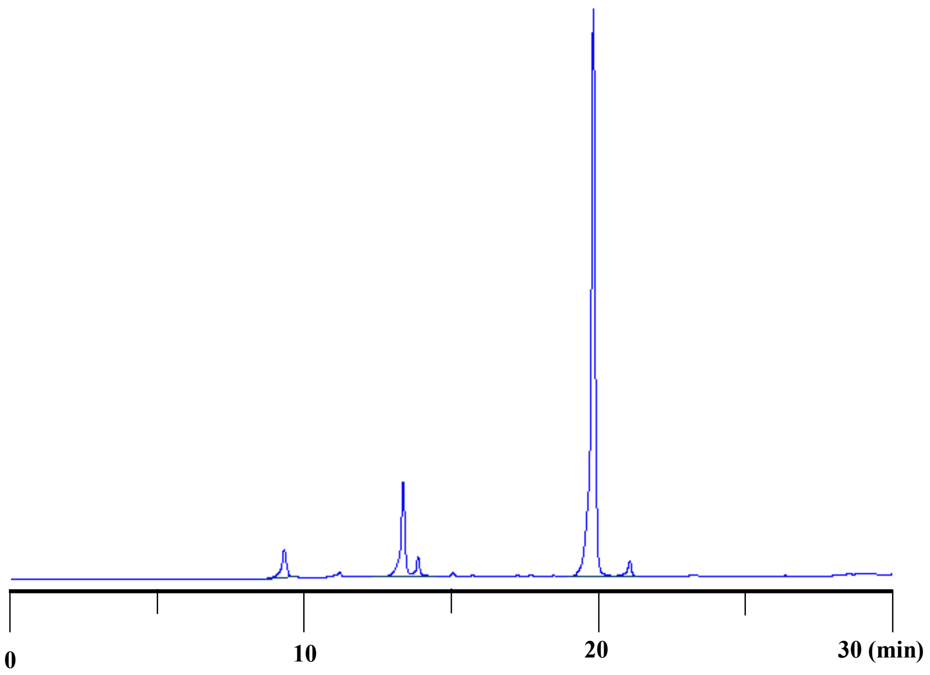

Figure S60 RP-HPLC profiles of dC$_{PN}$T dimer in **Table S3**, Entry 3

RP-HPLC (C18 (5 µm, 100 Å, 3.9 × 150 mm), 0.1 M triethylammonium acetate buffer (pH 7.0)/MeCN = 100/0–70/30 over 30 min, flow rate = 0.5 mL/min, l = 260 nm, t = 30°C) tR = 20.0 min

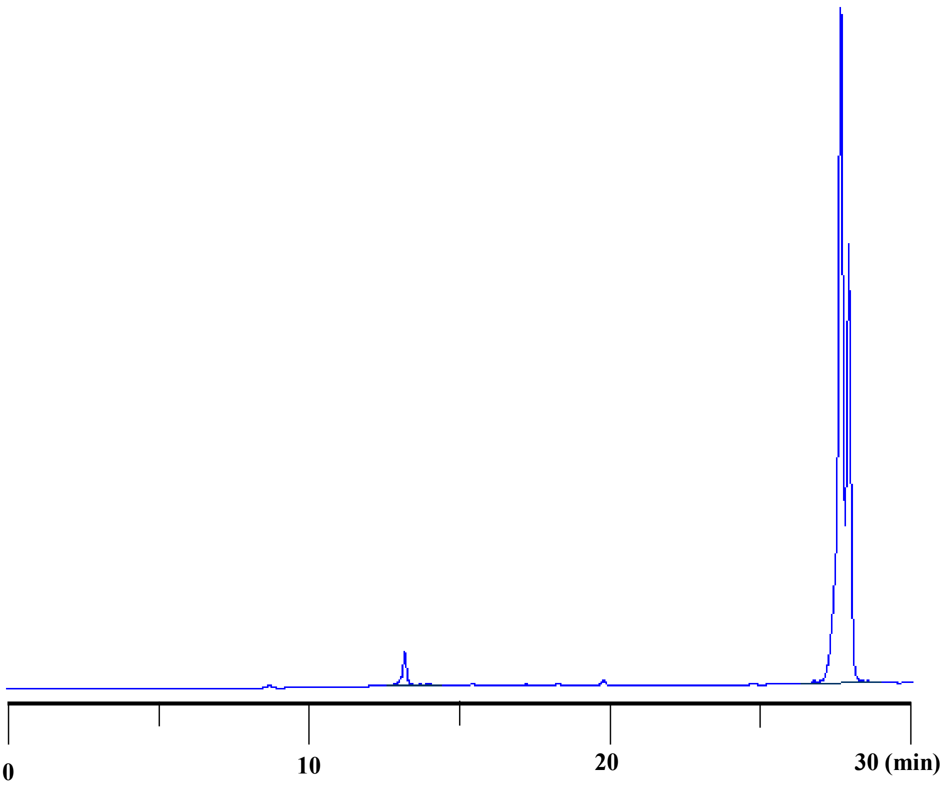

Figure S61 RP-HPLC profiles of dC$_{PSN}$T dimer in **Table S3**, Entry 4

RP-HPLC (C18 (5 μm, 100 Å, 3.9 × 150 mm), 0.1 M triethylammonium acetate buffer (pH 7.0)/MeCN = 100/0–70/30 over 30 min, flow rate = 0.5 mL/min, l = 260 nm, t = 30°C) tR = 27.9, 28.2 min (diastereomers)

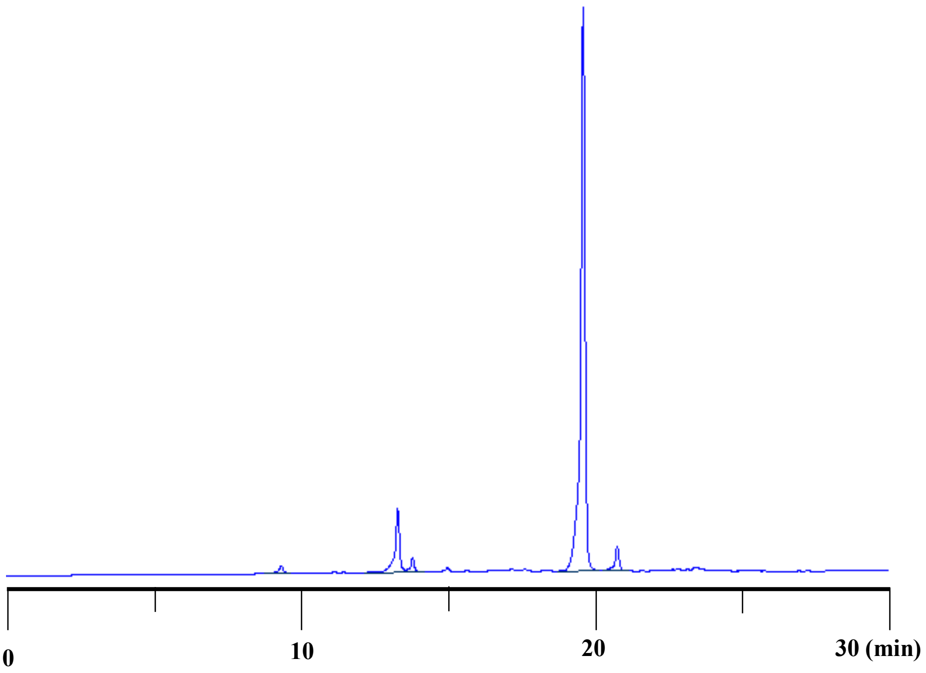

Figure S62 RP-HPLC profiles of dC$_{PN}$T dimer in **Table S3**, Entry 5

RP-HPLC (C18 (5 μm, 100 Å, 3.9 × 150 mm), 0.1 M triethylammonium acetate buffer (pH 7.0)/MeCN = 100/0–70/30 over 30 min, flow rate = 0.5 mL/min, l = 260 nm, t = 30°C) tR = 19.8 min

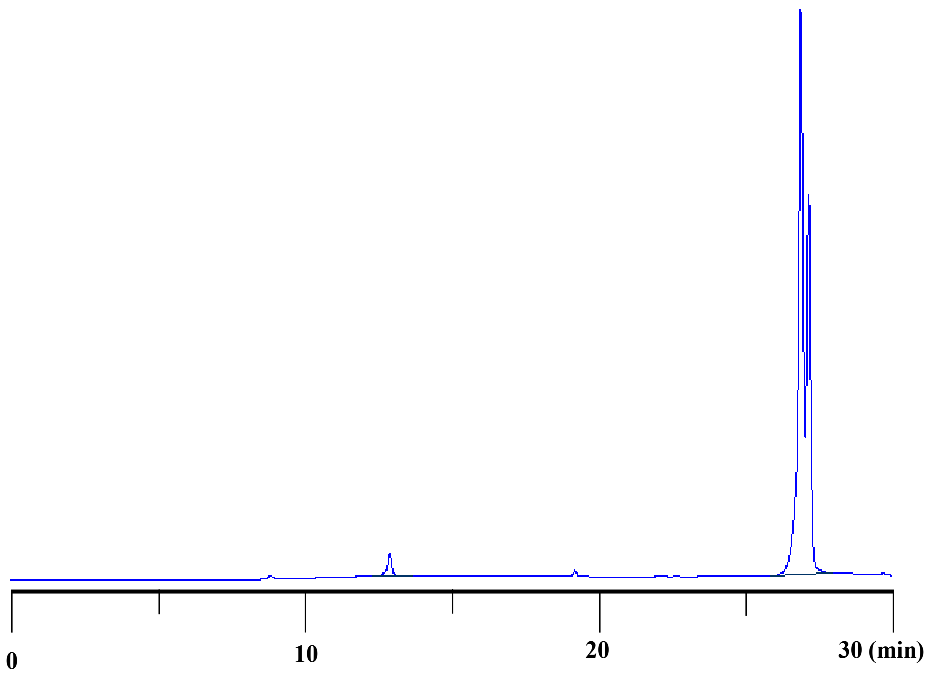

Figure S63 RP-HPLC profiles of dC$_{\mathrm{PSN}}$T dimer in **Table S3**, Entry 6

RP-HPLC (C18 (5 µm, 100 Å, 3.9 × 150 mm), 0.1 M triethylammonium acetate buffer (pH 7.0)/MeCN = 100/0–70/30 over 30 min, flow rate = 0.5 mL/min, l = 260 nm, t = 30°C) tR = 27.0, 27.3 min (diastereomers)

RP-UPLC profiles of crude PSN/PN chimeric pentamer (15)

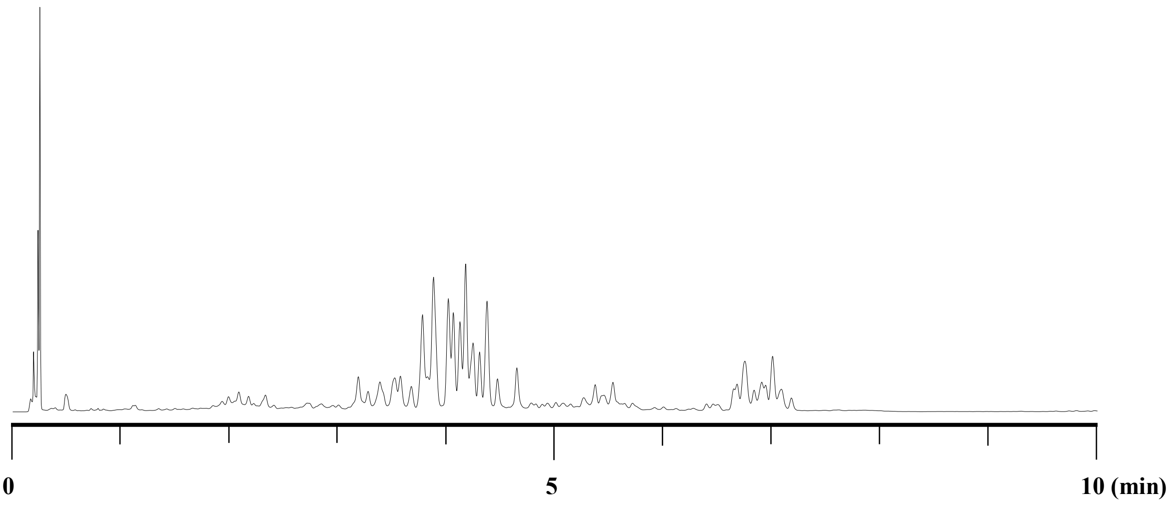

Figure S64 RP-UPLC profiles of crude d(T$_{\mathrm{PN}}$C$_{\mathrm{PSN}}$A$_{\mathrm{PN}}$G$_{\mathrm{PSN}}$T) (**15**) pentamer

RP-UPLC (C18 (1.7 µm, 130 Å, 2.1 × 50 mm), solution E/solution F = 80/20–30/70 over 10 min, flow rate = 0.6 mL/min, l = 260 nm, t = 45°C) tR = 3.79, 3.89, 4.02, 4.07, 4.13, 4.18, 4.25, 4.31, 4.38, 4.65 min (diastereomers)

Solution E: 50 mM HFIP and 5 mM TEA aq
Solution F: solution C–MeCN (1:1, v/v)

RP-HPLC profiles of pure PSN/PN chimeric pentamer (15)

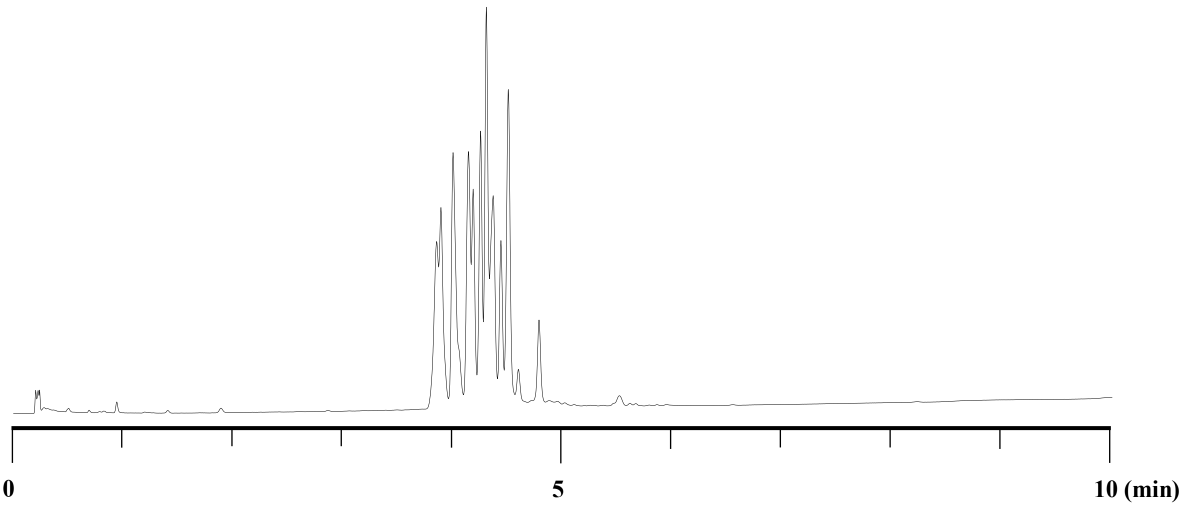

Figure S65 RP-UPLC profiles of purified PSN/PN chimeric pentamer (d($T_{PN}C_{PSN}A_{PN}G_{PSN}T$)) (**15**)

RP-UPLC (C18 (1.7 µm, 130 Å, 2.1 × 50 mm), solution E/solution F = 80/20–30/70 over 10 min, flow rate = 0.6 mL/min, l = 260 nm, t = 45°C) tR = 3.86, 3.90, 4.00, 4.14, 4.19, 4.25, 4.31, 4.37, 4.44, 4.50, 4.78 min (diastereomers)

Analysis of PSN/PN chimeric pentamer by LC-MS/MS

LC-MS/MS analysis of the PSN/PN chimeric pentamer was performed, similar to the analysis of PS_2/PS chimeric oligonucleotides. The detection of relevant fragment ions confirmed the sequence and types of internucleotidic linkages are identical to the desired ones (**Figures S66–S68**).

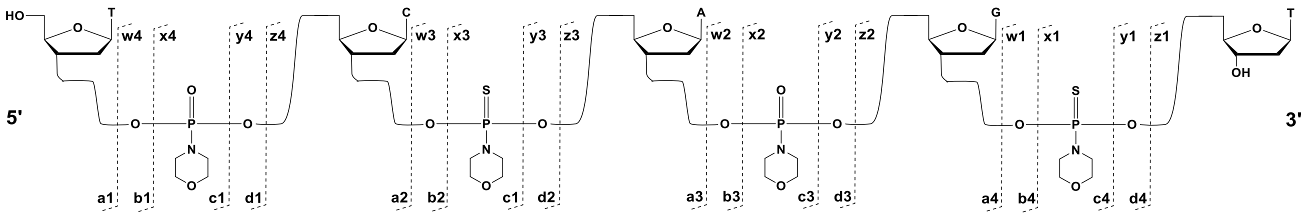

Figure S66 Structure of fragment ions

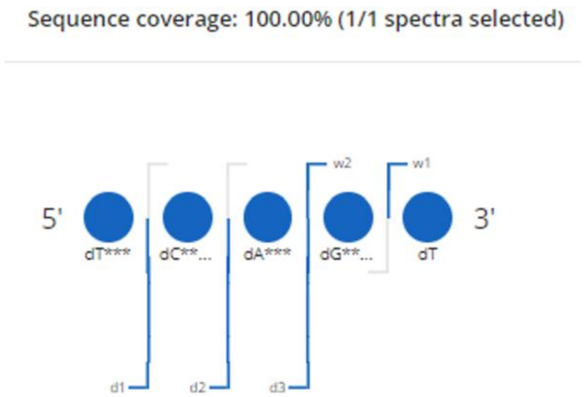

Figure S67 The list of fragment ions (*** denotes PS linkages, **** denotes PS_2 linkages)

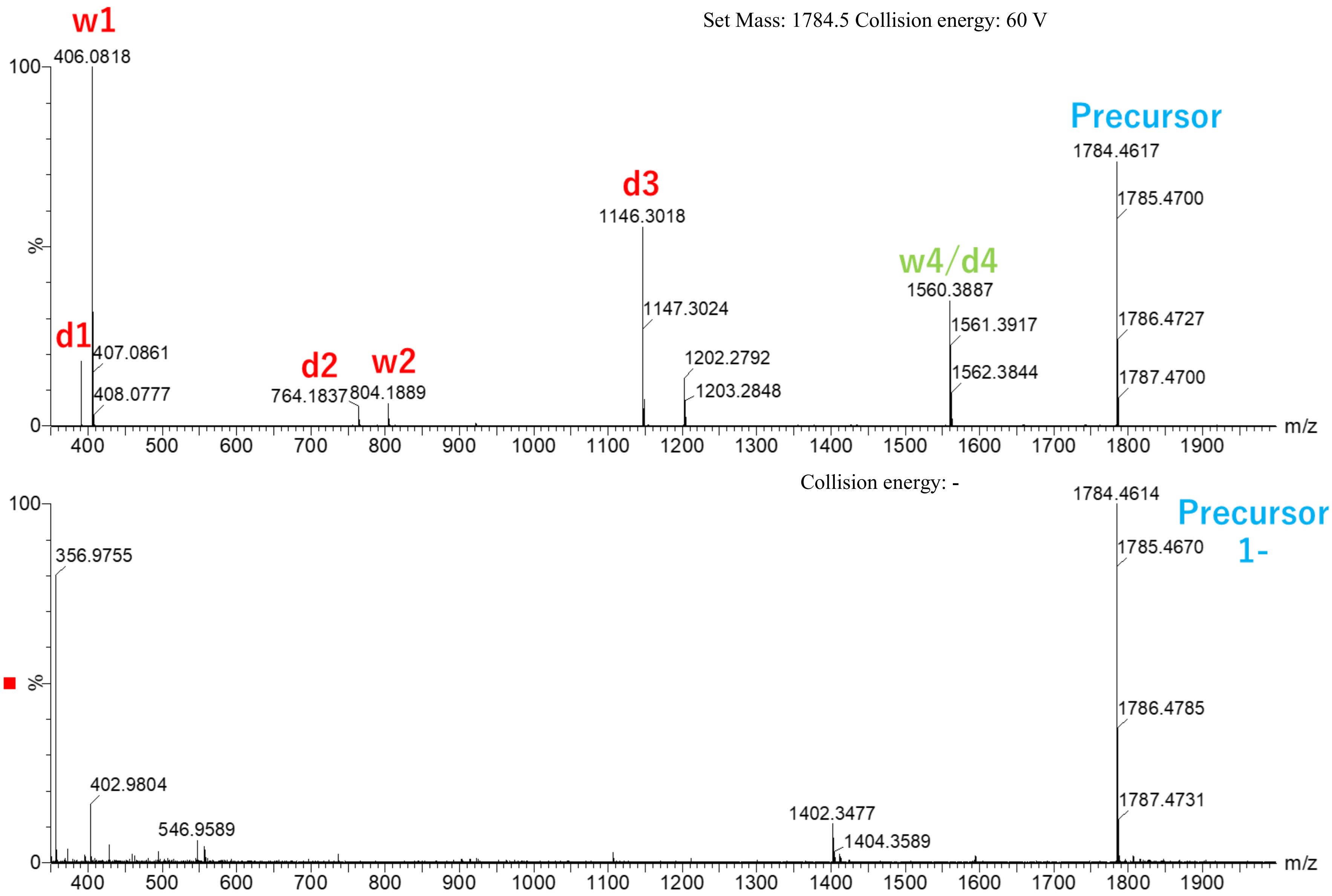

Figure S68 MS/MS spectra of the PSN/PN chimeric pentamer

Reference

(1) Cieslak, J.; Jankowska, J.; Stawinski, J.; Kraszewski, A. Aryl H-Phosphonates. 12. Synthetic and 31P NMR Studies on the Preparation of Nucleoside H-Phosphonothioate and Nucleoside H-Phosphonodithioate Monoesters. *J. Org. Chem.* **2000**, *65*, 7049–7054. https://doi.org/10.1021/jo000729q.

(2) Takahashi, Y.; Sato, K.; Wada, T. Solid-Phase Synthesis of Boranophosphate/Phosphorothioate/Phosphate Chimeric Oligonucleotides and Their Potential as Antisense Oligonucleotides. *J. Org. Chem.* **2022**, *87*, 3895–3909. https://doi.org/10.1021/acs.joc.1c01812.
